# Cosmopolitan Gene Families With Known Functions Are Hotspots for the Evolution of Novel Genes in Stony Corals

**DOI:** 10.1093/gbe/evag072

**Published:** 2026-03-24

**Authors:** Timothy G Stephens, Arkadiusz W Kulczyk, Debashish Bhattacharya

**Affiliations:** Department of Biochemistry and Microbiology, Rutgers University, New Brunswick, NJ 08901, USA; Department of Biochemistry and Microbiology, Rutgers University, New Brunswick, NJ 08901, USA; Institute for Quantitative Biomedicine, Rutgers University, Piscataway, NJ 08854, USA; Department of Biochemistry and Microbiology, Rutgers University, New Brunswick, NJ 08901, USA

**Keywords:** unknown function, genes, corals, dark genes

## Abstract

Climate change has accelerated research on biodiverse coral reef ecosystems. However, this area of investigation is limited by our understanding of the biology of these organisms, with many of the genes identified as important for stress response in corals being “dark,” that is, with no ascribable biological function. To aid reverse genetic efforts, and help explore dark gene evolution in this lineage, we analyzed available genomic and transcriptomic data from corals with the goal of identifying well conserved (often lineage specific) dark gene families and interrogating their putative roles in coral biology using available multi-omics data and bioinformatic approaches. Many of the well conserved dark gene families are stress responsive, enriched in specific cell types, or have predicted 3D protein structures with significant similarity to known proteins that may be adaptive in corals. We demonstrate that dark genes form cosmopolitan (broadly shared) families which originated *via* bursts of lineage specific duplication, often involving genes with known functions. Analysis of single cell gene expression data suggests that dark gene provenance may have precipitated or been concomitant with the origin of novel coral functions such as biomineralization. Our results open a new window into coral evolution that integrates knowledge from known and dark genes to elucidate how these species achieved their remarkable success in diverse marine environments. The dark gene families we identified also provide a significant resource for future studies into the role of novel genes in coral biology and adaptation to climate change.

Significance Many of the genes identified as important for stress response in corals are “dark,” that is, they have no ascribable biological function. To aid characterization of these dark genes, our study analyzed available coral genomic and transcriptomic data, identifying well conserved (often lineage specific) dark gene families that are stress responsive or enriched in specific cell types. We suggest that dark genes originated *via* bursts of lineage specific duplication, often from genes with known functions. These results open a new window into coral evolution, with the dark gene families identified by this study providing a significant resource for future research into the function and role of dark genes in coral biology and adaptation to climate change.

## Background

The significant threat posed by anthropogenic climate change has spurred conservation research (Cheng et al. 2023; Orr et al. 2005). Reefs mainly formed by stony corals (Scleractinia) are one of the highest value threatened ecosystems, hosting about one-quarter of all marine biodiversity (Hughes et al. 2017). Reference genome and transcriptome datasets, as well as differential gene expression, single cell RNA-seq, and other multi-omics data exist from different stony coral species (Bhattacharya et al. 2016; Levy et al. 2021; Shinzato et al. 2021; Stephens et al. 2022; Williams et al. 2023). These data have advanced understanding of the coral stress response, however, very little is known about novel pathways and processes that evolved in Scleractinia. Genes of unknown function, also known as “dark” genes, which are often taxonomically restricted to particular lineages (also known as orphan genes [ORFans] or taxonomically restricted genes) (Fakhar et al. 2023; Fischer and Eisenberg 1999; Stephens et al. 2018), may represent a significant proportion of the nuclear gene inventory. In the rice coral, *Montipora capitata*, 11.7% of predicted protein-coding genes do not have significant hits (BLAST *e*-value cut-off ≤1e^−10^) to proteins in the nonredundant NCBI database, and are considered dark (Shumaker et al. 2019). In dinoflagellates, that includes Symbiodiniaceae (algal photosymbionts of corals), 36.6% of genes do not share significant sequence similarity to proteins in UniProt (Stephens et al. 2018). Even in well-studied systems such as *Drosophila* or humans, many genes have unknown or putative functions despite presenting clear phenotypes when knocked out (Rocha et al. 2023). More broadly, 30% to 50% of environmental proteins from prokaryotes, eukaryotes, and viruses cannot be annotated with a function (Almeida et al. 2021; Carradec et al. 2018; Price et al. 2018). The role of dark genes and the mechanisms which generate them (ie de novo gene evolution or rapid neofunctionalization after gene duplication) (Tautz and Domazet-Lošo 2011; Weisman and Eddy 2017; Weisman et al. 2020; Weisman 2022), are poorly understood. This is a critical issue because highly conserved gene families with canonical, known functions such as in the cell cycle, central metabolism, and the cytoskeleton are potentially less likely to play pivotal roles in lineage-specific adaptations that distinguish phyla. Yet, these genes are the “bread and butter” of many comparative analyses in non-model systems due to the lack of genetic tools to study dark genes.

To address this shortfall, we used transcriptome and genome data to investigate dark gene evolution across Cnidaria, with a particular focus on stony corals (Scleractinia). We identified a suite of well conserved dark gene families, some of which show intriguing patterns of stress and cell type-specific expression, and others encoding proteins that may have played important roles in coral evolution, and whose structures can be predicted with high confidence. Many of the predicted models share significant structural similarity with proteins of known functions whose structures have been determined experimentally. In addition, we demonstrate that, despite being restricted to a narrower lineage breadth, dark genes contain comparable phylogenetic signal to annotated (“light” [with a putative known function]) genes. That is, the phylogenies constructed from dark gene families closely mirror those of light gene families. Lastly, through an all-versus-all gene family analysis using remote homology detection, we identified bursts of lineage-specific dark gene origination, putatively from more cosmopolitan (broadly shared; often light) gene families. Our analysis supports the theory that many dark genes originate from the duplication and rapid mutation (and putative neofunctionalization) of existing broadly distributed gene families. Many of these bursts of dark gene origination may have coincided with major evolutionary transitions, promoting the evolution of novel functions, cell types, or regulatory processes. Our results provide an in-depth view into the content and evolution of the cnidarian dark proteome and provide a wealth of targets for follow-up functional studies and protein structure determination, reverse-genetics, or other functional assays to explore dark gene families that form pillars of coral evolution.

## Results

### Orthogroup Analysis and Dark Gene Identification

**Fig. 1.**
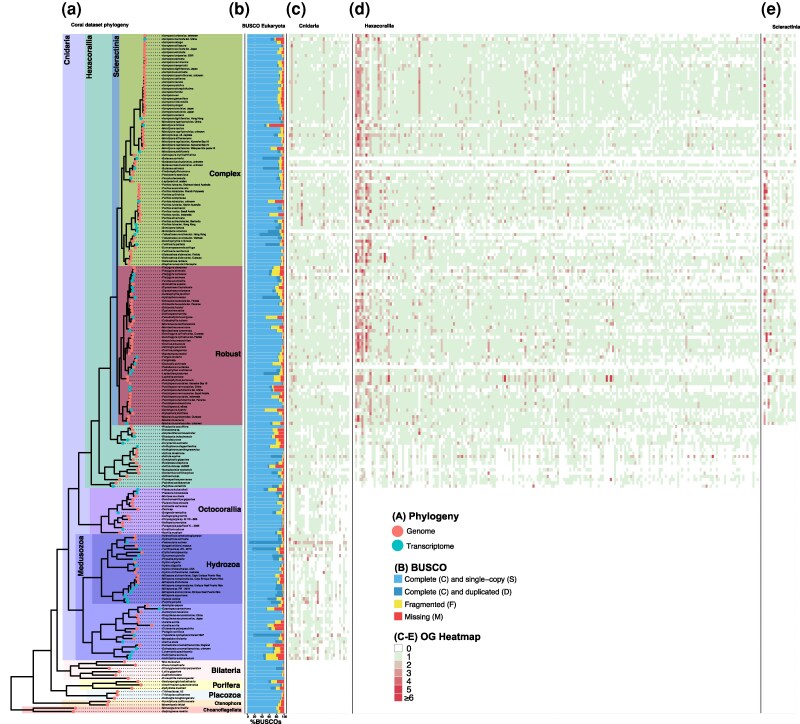
Summary of OG designations and distribution of sequences in conserved dark OGs. a) Phylogeny constructed by STAG using OGs with sequences from each of the 205 datasets with < 50% missing BUSCO genes. Red and blue circles at the tips of the phylogeny denote genome and transcriptome datasets, respectively. Major taxonomic groups in the phylogeny are denoted with colored squares and text labels. b) The BUSCO (Eukaryota) completeness of proteins in each genome and transcriptome dataset. (c–e) The heatmap describes the number of proteins from each dataset in each of the conserved dark OGs that are highly conserved across c) Cnidaria, d) Hexacorallia, and e) Scleractinia. The colors used in the bar charts and heatmap (b–e) panels are described on the bottom right of the figure.

A total of 228 genome and transcriptome datasets were retrieved for publicly available databases. After filtering to remove incomplete (ie >50% missing genes in the Benchmarking Universal Single-Copy Orthologs [BUSCO] data set (Manni et al. 2021)) or redundant genomes and transcriptomes, 205 datasets comprised 189 Cnidaria (118 from Scleractinia and 71 from other non-Scleractinia cnidarians) and 16 outgroups (3 Placozoa, 4 Porifera, 2 Ctenophora, 2 Choanoflagellata, and 5 Bilateria) remained for use in this study (Fig. 1; Tables S1 to S3). The 16 outgroup datasets were used primarily to root the constructed phylogenies and were not intended to encompass the full diversity of species outside Cnidaria. The selected datasets comprised 7,137,414 proteins that were clustered into 793,054 orthogroups (OGs). Our annotation approach (which considers all proteins in an OG as annotated if one member has a hit to the NCBI nr database with a described putative function; see Methods and the section “Orthogroup analysis and dark gene classification” in the Supplementary Information for details) classified 11.4% of proteins as dark (unannotated).

### Expression Patterns of Dark Genes Across Cnidaria

The expression patterns of dark versus light genes were assessed using three RNA-seq (two from *Montipora capitata* and one from *Pocillopora acuta*) (Stephens et al. 2023; Williams et al. 2021) and six single-cell (sc)RNA-seq (*Stylophora pistillata* adult, polyp, and larva life history stages, *Nematostella vectensis*, *Xenia* sp., and *Hydra vulgaris*) (Hu et al. 2020; Levy et al. 2021; Sebé-Pedrós et al. 2018; Siebert et al. 2019) datasets. The null hypothesis in this analysis was that the proportion of dark genes significantly expressed in each condition or cell type in each dataset would roughly match that of light genes. That is, no bias exists in the distribution of dark gene expression and any observed differences are randomly distributed among the data.

The proportion of light and dark genes with significant differential expression in one or multiple RNA-seq (FC > 0.5 and adjusted *P*-value < 0.05) and scRNA-seq (FC > 2) datasets was higher for light than dark genes (Dataset S1), with the sole exception being the *H. vulgaris* scRNA-seq dataset (Dataset S1, Page S11). This suggests that light genes are more likely to be involved in the transcriptional response to the analyzed treatment conditions (lowered pH and heat stress) and in the differentiation of the different cell types analyzed. The proportion of dark and light genes that were significantly differentially expressed in the different treatments and cell types was roughly equal: ie treatments or cell types with a high proportion of light genes also tended to have a high proportion of dark genes (out of all light and dark genes with significant differential expression).

In the scRNA-seq datasets, calicoblast cells (which generate the calcareous coral exoskeleton) were only present in the *S. pistillata* adult and polyp datasets, but in both cases had higher proportions of differentially expressed dark genes at all levels of cell type granularity (Dataset S1, Pages S6&S7; “Broadcell” is the lowest granularity, followed by “Cell,” then “Metacell,” which is the highest granularity and below the level of cell type). Similar trends were observed in other cell types, such as gastrodermis and epidermis (see section “Expression patterns of dark genes across Cnidaria” in the Supplementary Information). In each of the examples described above, exceptions occur at more granular levels. For example, there is a higher proportion of dark genes with significant expression in the *H. vulgaris* cnidocyte “broad” cell type (Dataset S1, Page S11 top panel). In the “cell” and “metacell” types some of the identified groups of cells have higher proportions of light genes, with the majority however, having higher proportions of dark genes (middle and bottom panels).

### Selection of Dark OGs With Strong Conservation Across Key Lineages

**Fig. 2.**
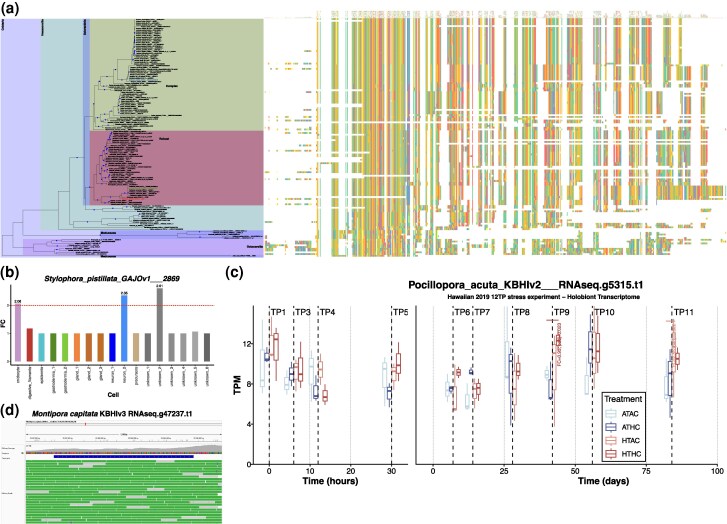
Phylogeny, structure, and expression of dark genes in cnidarian dark orthogroup OG000011274. a) ML phylogeny of the protein sequences in this OG, with associated alignment shown on the right. Nodes with >95% bootstrap support are annotated with blue circles. The sequence chosen to represent the OG is highlighted with red text, sequences from *M. capitata* KBHIv3 with blue text, *P. acuta* KBHIv2 with green text, and *S. pistillata* GAJOv1 in purple text. b) Fold change (FC) of the one *S. pistillata* gene in the OG, at a meta cell level, showing that the gene is expressed above the >2.0 FC cutoff (dotted horizontal red line) used to signify significant cell type specific expression in this study. c) Differential gene expression results for the one *P. acuta* gene in the OG. d) IGV visualization of RNA-Seq reads mapped against the *M. capitata* KBHIv3 reference genome, showing concordance between the exon structure of *M. capitata* KBHIv3 and the transcriptome data used for this analysis.

Dark OGs comprised of genes from ≥50% of the Hexacorallia, Scleractinia, or Cnidaria datasets were considered highly conserved and selected for downstream analysis (Fig. 1). Each of these conserved dark OGs were used as input for phylogenetic, protein structure, Hidden Markov Model (HMM)-based functional, and gene expression analyses to aid their characterization in corals and to identify potential hot spots for functional innovation. There were 244 dark OGs which satisfied the above criteria (Table S2), 30 for Cnidaria, 198 for Hexacorallia, and 16 for Scleractinia. Overall, 138/244 conserved dark OGs (56.56%) had perfect, or near perfect congruence (agreement) across at least one (if multiple were present) of the predicted gene models in *M. capitata* (KBHIv3) and the aligned RNA-seq data (ie the aligned RNA-seq data matched the exon/intron pattern of each gene model), demonstrating that they are unlikely to be artifacts from ab initio gene prediction (see section Selection of conserved dark OGs” in the Supplementary Information). The phylogeny inferred for each of the conserved dark OGs (eg Figure 2) share high similarity with the species tree (Fig. 1), with much of the observed variation arising from incomplete or mis-predicted sequences (eg Figure 2a, *Alatina alata* in the bottom of the phylogeny).

Each of the conserved dark OGs were analyzed using abSENSE, a tool designed to predict if the absence of proteins from a particular taxonomic group (outside the lowest common ancestor [LCA] of that OG) is due to homology detection failure (HDF; resulting from a high rate of sequence evolution) or if it is truly missing from that lineage. Of the conserved dark OGs, 201 have ≥ 50% of the taxa outside their LCA with evidence of HDF, and only 19 have ≥ 50% of taxa outside their LCA with evidence against HDF (Figs S1E, S1F; Table S2). Thus, the majority (82.4%; 201/244) of conserved dark OGs show evidence of HDF, suggesting these gene families may have evolved from other, possibly light (annotated), gene families and subsequently underwent rapid sequence evolution, making them dark. Conversely, only 7.8% (19/244) of the conserved dark OGs show weak evidence for HDF, suggesting that these gene families may have arisen *via* de novo gene origination.

### 3D Protein Structure of Conserved Dark OGs

**Fig. 3.**
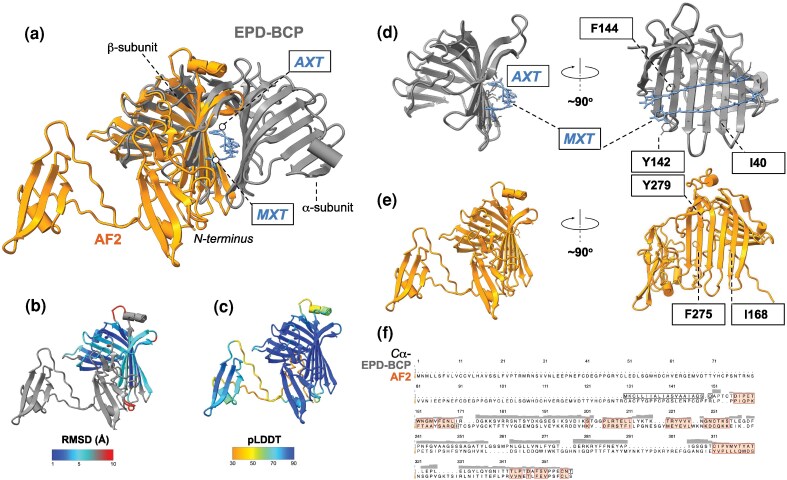
Predicted structure of protein encoded by OG000001644 and model of dark gene family evolution. a) The X-ray structure of EPD-BCP (PDB ID: 8i34) (Kawasaki et al. 2023) showing the heterodimeric protein composed of two, α and β, subunits (both displayed in gray) with two carotenoids (astaxanthin, AXT and mytiloxanthin, MXT, shown in blue) bound at the inter-subunit interface superposed onto the monomeric AF2 model (OG000001644, shown in orange) composed of two domains connected by a linker. b) The AF2 model and the β subunit of EPD-BCP reveal a significant structural homology with root mean square deviations (RMSDs) ranging from 1 Å to 5 Å across the entire homologous parts of the proteins including the ligand-binding surface in the β subunit a), and 23% sequence identity (f). c) The accuracy of AF2 prediction is reflected by high predicted local distance difference test (pLDDT) values (ie >90) across the entire model except an inter-domain linker and a short α-helix in the C-terminal domain. (d, e) Structural details of EPD-BCP and the AF2 model are displayed, respectively. AXT and MXT (displayed in blue) engage in specific interactions with the residues lining the outer face of the β-sheet in the β subunit of EPD-BCP, for example I40, Y142, and F144. The AF2 model displays a similar arrangement of residues (eg I168, F275 and Y279, respectively) located in analogous positions. The above structural analysis collectively suggests that the coral protein may also function as a dimer (e). f) Sequence alignment of EPD-BCP and the AF2 model (OG000001644). The top row in the figure indicates similarities in the C_α_ alignment.

AlphaFold v2.3.1 (AF2) was used to predict the 3D structure of each conserved OG representative sequence, producing 33 models (13.5% of the 244 conserved dark OGs) with high (>70%) predicted local distance difference test (pLDDT) values, with the remaining structures modeled with lower confidence (see Methods for pLDDT ranges). Five well resolved AF2 structures (see the section “Selection of AF2 Structures for Manual Analysis” in the Supplementary Information for details on how these structures were selected) were compared against crystal and cryo-EM structures in the RCSB Protein Data Bank (PDB) (Dataset S2). This analysis identified conserved folds with significant structural homology, but low sequence similarity (<23%), in each of the five cases, with putative functions proposed to be an ependymin-related blue carotenoprotein (EPD-BCP; OG000001644; see below), Nitrophorin 4 (Np4; OG000010602; Dataset S2, Page S1), Chondroitin B lyase (ChonB; OG000009717; Dataset S2, Page S2), Superkiller 8 (Ski8; OG000008812; Dataset S2, Page S5), and large subunit of the mitoribosome (ml115; OG000011843; Dataset S2, Page6). The EPD-BCP structure (see below in Figs. 3a-f) is of particular interest because corals are renowned for their ability to exhibit a wide range of colors (eg *via* green and red fluorescence proteins) (Alieva et al. 2008; Ben-Zvi et al. 2022), therefore OG000001644 may represent a novel blue carotenoprotein (Kawasaki et al. 2023) in this lineage that is derived from the ancestral ependymin encoding gene family. These results suggest that dark OGs may have diverse functions, such as fluorescence, regulation, enzymatic cleavage, and nitrate transport. While functional and structural validation is required to confirm these putative assignments, our work demonstrates the potential of large-scale gene family analysis coupled with protein structure inference to identify novel protein classes that function in key cellular processes critical to organismal biology. Given the overall poor performance of AF2 on the conserved dark OGs (86.5% of proteins having low or very low pLDDT values), the performance of AF2 with highly conserved, broadly distributed light coral proteins was assessed against the PDB database to ascertain the validity of the predicted models. Of the 243 well conserved light coral proteins used to assess the performance of AF2 (see section “3D protein structure of light genes” in the Supplementary Information), all but 26 (10.7%; Table S3) had structures predicted with confidence of very high (pLDDT > 90) or confident (pLDDT > 70). Most structures (91.36%) had top scoring alignments against the PDB database with a TM-score > 0.5, demonstrating that their predicted structure is congruent with verified reference structures. This suggests that the poorly resolved structures of the conserved dark OGs (Table S2) is not an artifact of the diverged lineages being studied and might be due to the existence of novel folds.

### Putative Functional Domains in Conserved Dark OGs

Of the conserved dark OGs, 161 (65.98%) had functional domains assigned by EggNOG-mapper or InterProScan, transmembrane regions identified by TMHMM (part of InterProScan), or 3D structural similarity (predicted by Foldseek) to other proteins in PDB or the AlphaFold-UniProt database (Fig. S2). Of these 161 OGs, 115 (71.43%) are putatively membrane associated and 46 (28.57%) possibly function in other cellular processes such as metabolism or substrate binding. The expression patterns of the conserved dark OGs varied greatly, with many showing differential expression in *M. capitata* or *P. acuta* under thermal or CO_2_ stress conditions, and others showing differential expression in one or multiple cell types in the six analyzed scRNA-seq datasets. Some examples of conserved dark OGs with interesting expression patterns are shown in Dataset S3. The three OGs highlighted in Dataset S3 were chosen because they have significant expression in adult calicoblast cells, and high but sometimes not significant expression in polyps (Dataset S3, panels C-E of each page). These three OGs also showed increased expression under pCO_2_ and thermal stress conditions at later time points in *P. acuta* (Dataset S3, panel F of each page). One of these OGs shows significant expression in some *N. vectensis* neuron and muscle cell types (Dataset S3, Page S2 panel G).

### Network Analysis of Remote Homology Between Orthogroups

**Fig. 4.**
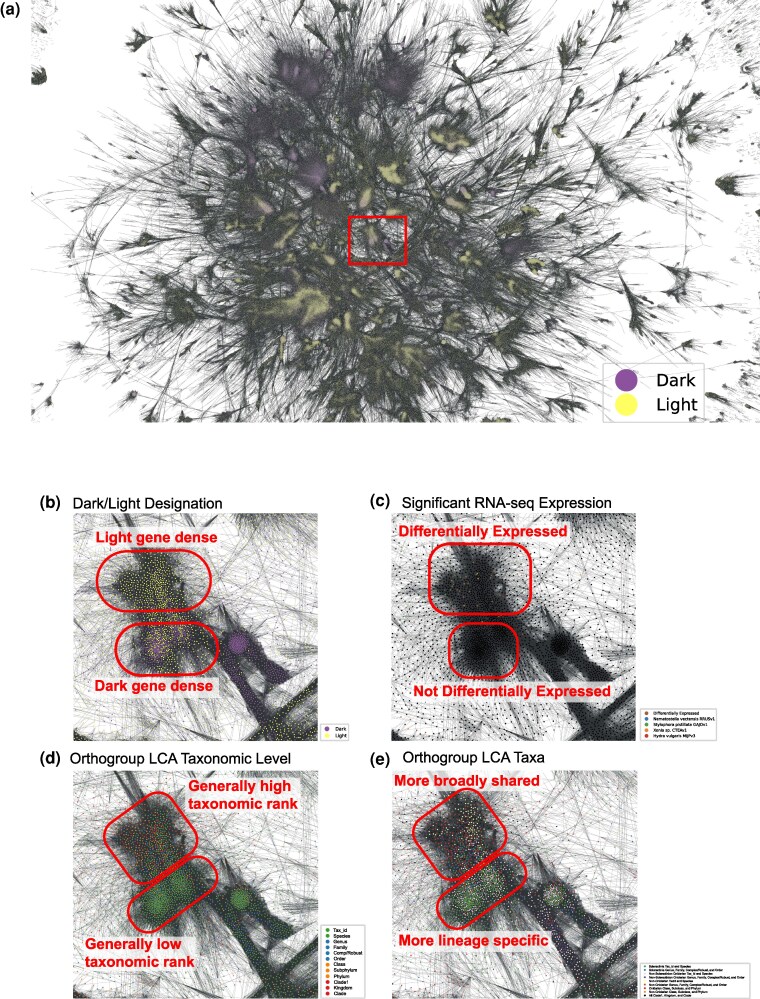
OG remote homology network. OG network, centered around the largest connected components, with peripheral singleton nodes cropped from view. Nodes (OGs) colored based on their designation as either dark (purple) or light (yellow). Edges represent filtered (probability ≥ 90%, query coverage ≥ 60%, and subject coverage ≥ 60%) hhblits hits, connecting nodes with putative (often weak) homology. (b–e) Zoomed-in snapshot of a subregion from the full network (red box in [a]), with nodes colored based on b) their designation as either dark or light, c) the presence of proteins with significant expression across stress and scRNA-Seq datasets, d) the LCA assigned taxonomic level, and e) the LCA assigned taxa. In panel (c), if an OG had proteins that showed significant expression across multiple datasets, then colored segments were used to represent each of the datasets associated with the node. In each panel, the layout was calculated using the SFDP algorithm. A legend describing the color used in each snapshot is shown in the bottom right of each panel. Red ellipses in b–e represent segments of biased node distribution, according to the factors represented by each color scheme.

HMMs were generated for all 793,054 OGs to facilitate a more sensitive comparison of remote homology using the hhblits program and the procedure set forth in the AGNOSTOS workflow (Vanni et al. 2022). After filtering to remove hits between OGs with low (<90%) probability and low (<60% query or subject) sequence coverage, 791,748 remained with connections to at least one other OG for downstream network analysis. The final network had 791,748 nodes (OGs), 32,318,532 edges, and 43,513 connected components, with the largest component comprising 214,209 nodes. There is distinct structure to the full network and subnetworks that comprise predominantly dark or light OGs (Fig. 4a), with regions where the two groups are intermixed. The large, tightly connected subnetworks (an example of which is shown in Fig. 4b-e) also tend to be comprised of single species or single isolate OGs from the same group of taxonomic lineages. An example of this phenomenon is presented in Figs. 4b-3e. It is also interesting to note that these dense subnetworks are interspersed with OGs assigned to higher taxonomic levels, and “higher” taxa that are related to the lower classified OGs. That is, the isolate level OGs tend to be positioned with genus or higher-level OGs from related taxa. For example, there are clearly two subnetworks comprised predominantly of species or isolate level OGs (green nodes in Figs. 4d and green and pink nodes in Fig. 4e), which group alongside phylum or clade level OGs (red and black nodes in Fig. 4e). There is a clear grouping of OGs that comprise genes that have differential expression in one or multiple of the differential expression RNA-seq or scRNA-seq datasets. All the trends highlighted in this figure are observed across the full network presented in Dataset S4 and support the observation that these attributes are not randomly distributed throughout the network.

## Discussion

Cnidarian dark genes form well conserved families (OGs) involved in major stress response or cell type-specific functions. The higher proportion of differential dark gene expression in specific cell types, such as calicoblasts in *S. pistillata* (Dataset S1, Pages S6&S7), suggests that evolution of these genes may be tied to major evolutionary transitions in corals, such as the ability to biomineralize. Our analysis reveals that many classes of ancestral cell types, such as cnidocytes in *H. vulgaris* (Dataset S1, Page S11 bottom panel) have specific sub-cell types that have a higher proportion of differentially expressed dark genes compared to other cell types that show a higher level of differentially expressed light genes. This suggests that even within existing classes of cells, dark genes play a key role in a lineage-specific manner, potentially contributing to the origin of derived cell types. Whereas many of these dark gene families did not yield high confidence 3D models with AF2, a significant subset had structures predicted with high accuracy and apparent homology to PDB reference structures (eg EPD-BCP, Figs. 3a-f). The accuracy of these predictions remains to be validated, nonetheless, our work provides a useful approach for generating testable hypotheses about putative dark gene functions. Many of the conserved dark OGs are predicted to encode proteins with transmembrane domains (TMHMM, Fig. S2), providing additional support for a functional role in corals. The predicted 3D structures of analyzed light genes had much higher average confidence than dark genes, with 89.3% (217/243) of the light genes having confident (>70) or very high (>90; 53/243) average pLDDT scores, compared to 13.5% (33/244) of the dark OGs. This suggests that tools such as AF2 may reliably predict the structure of broadly conserved proteins in these taxa.

### Radiation of Novel Genes From Existing Gene Families

The remote homology network presented in Fig. 4 supports the existence of “hot spots” of gene family evolution, with novel gene families (particularly dark gene families) having evolved from the duplication and neofunctionalization of cosmopolitan (broadly shared) gene families, which had also been suggested in previous research (Domazet-Loso and Tautz 2003; Tautz and Domazet-Lošo 2011). This hypothesis is supported by the structural homology and low (<25%) sequence identity of the conserved dark OGs (Dataset S2) vis-à-vis crystal structures in PDB. The grouping of large numbers of species or isolate-specific OGs with a limited number of (often light) OGs that are present across a large breadth of taxa suggests that the latter may have given rise through duplication and neofunctionalization to the former. Whereas the remote homology network (Fig. 4) could be used to infer annotations to many of the connected dark OGs, it is unknown if such highly derived proteins will retain their ancestral functions. Therefore, further study of these sequences is required to understand the functions consequences of this radiation of dark genes.

### Model of Dark Gene Evolution

**Fig. 5.**
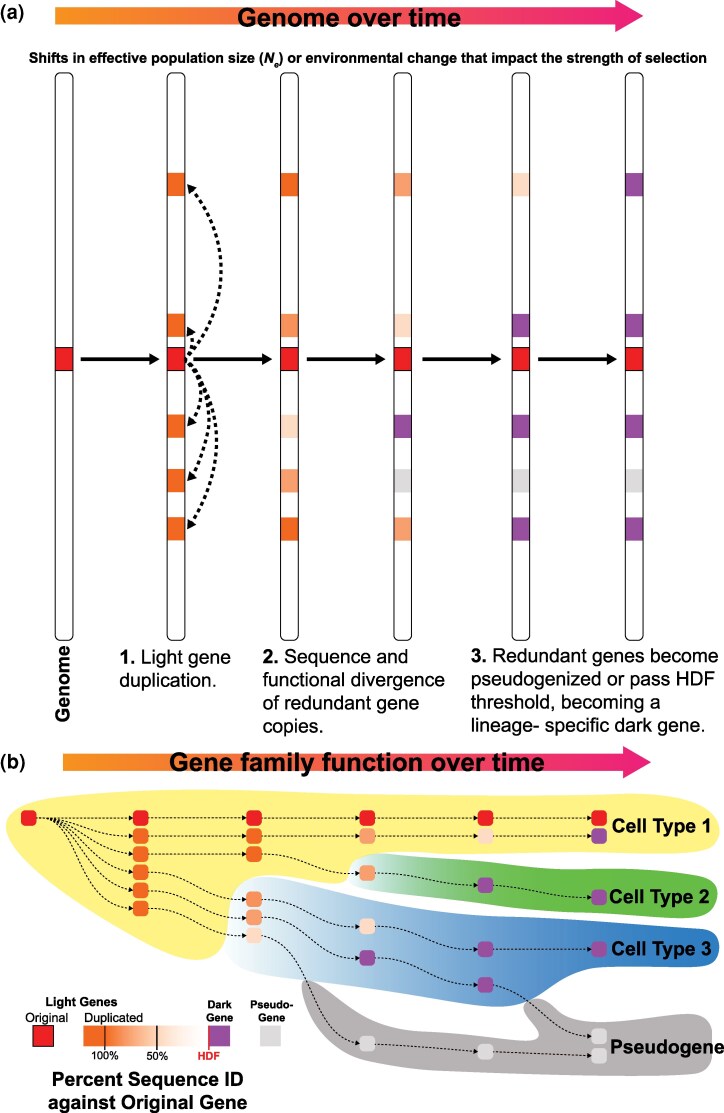
Model of coral dark gene evolution. a) The evolution of a light gene family (red box) in the genome of an organism. In this example, the light gene family starts as a single copy on the left of the figure, is duplicated in the genome (orange boxes), the duplicated copies diverge (lighter orange), either becoming pseudogenes (gray) or dark genes (purple) when they eventually exceed the limit of homology detection (ie Homology Detection Failure [HDF]). b) Functional evolution of the duplicated gene copies (orange circles) shown in (a); here, cell type is used as a proxy for any combination of functional or regulatory pattern. Initially, all duplicated gene copies have the same functional profile (“Cell Type 1”) as the ancestral copy. Over time, the function of these genes may diverge as their sequences diverge, shown here as some genes transitioning to Cell Types 2 and 3. These Cell Types may be novel or a subtype of the parental cell line. Alternatively, the gene copies continue to function within the same parental cell type, taking on alternative functional roles from the ancestral light gene. At any stage, these genes may also become pseudogenes. Legend describing the colors used for the genes in the figure (boxes and circles) is shown in the bottom left.

Based on these results, we propose a model (adapted from the works of (Domazet-Loso and Tautz 2003; Khalturin et al. 2009; Noel et al. 2023; Shinzato and Yoshioka 2024; Tautz and Domazet-Lošo 2011) whereby dark genes are predominantly produced by the duplication and divergence of existing, often functionally important, light genes (Figs. 5a and b). The rapid accumulation of mutations in the duplicated genes (or ultimate loss, *via* pseudogenization) make detection of homology to light genes using sequence-based methods highly challenging. This process is expected to produce many lineage-specific dark genes (and dark gene families) with diverse functions that have arisen from a smaller set of ancestral light genes. The function of the generated dark genes may be related to the ancestral light gene, potentially suggesting that light genes with central roles in key adaptable processes (such as cell differentiation; Fig. 5b) may be hot spots for duplication and diversification into dark genes, however, this remains to be tested. Radiation of dark genes may be precipitated by environmental change or is an inherently dynamic process, with ongoing production at differing rates punctuated by bursts during evolutionary transitions. These processes likely occurred in the ancestor of Scleractinia, producing specialized cell types such as calicoblasts.

Major evolutionary transitions reshape the habitable world with the origin of photosynthesis in eukaryotes *via* primary endosymbiosis and the subsequent provenance of land plants from green algae providing two notable examples (Miyagishima 2023; Rockwell et al. 2014). The origin of stony corals is another example of a massive evolutionary transition for ocean life. Although challenging model systems, corals provide the opportunity to understand the basis of long-lived, reef building lifestyles that have fundamentally altered many near-shore environments. The demonstration of CRISPR/Cas9 knockdown of genes with known function in reef building species such as *Acropora millepora* (Cleves et al. 2018, 2020; Tinoco et al. 2023; Yasuoka et al. 2016) paves the way for its use with the genes of unknown function identified by this research. The study of dark genes will not only reveal many interesting insights into the evolution of life and novel functions, but will also enhance our understanding of coral biology, their response and capacity to adapt to climate change, and potentially open-up new opportunities for conservation and coral health monitoring. Given that many of dark gene families we identified are lineage specific, they may provide novel insights into coral biology, and offer highly promising targets for diagnostic tool development (Chille et al. 2025). The rapid expansion of approaches that predict high-confidence protein structures de novo (Abramson et al. 2024; Yurchenco and Kulczyk 2024) coupled with their experimental validation provides a promising path to advance knowledge of the coral dark proteome.

## Conclusion

Our results demonstrate the key roles played by dark genes in coral evolution, the putative mechanisms that generate dark genes in this group, and how a continual cycle of dark gene origination *via* duplication and neofunctionalization may underpin niche adaptation in corals. This work also provides a multitude of target dark gene families for discovering coral-specific functions using protein structure analysis, reverse genetic methods (eg in the hard coral *Astrangia poculata* [Warner et al. 2023]), and heterologous expression analysis in the starlet sea anemone *Nematostella vectensis* (Babonis et al. 2023).

## Methods

### Protein Dataset Acquisition

We collected all available high-quality genomes and transcriptomes from Cnidaria, and a representative set of datasets from the outgroup taxa Ctenophora, Placozoa, Porifera, choanoflagellates, and Bilateria for use in downstream phylogenetic analysis. In total, 228 datasets, available at the time our analysis were retrieved: 157 genomes (Baumgarten et al. 2015; Bernardi et al. 2024; Bongaerts et al. 2021; Buitrago-López et al. 2020; Celis et al. 2018; Chen et al. 2025; Conn et al. 2025; Cunning et al. 2018; Eitel et al. 2018; Fairclough et al. 2013; Fiesinger et al. 2024; Fuller et al. 2020; Helmkampf et al. 2019; Herrera and Cordes 2023; Hu et al. 2020; Ip et al. 2023; Jeon et al. 2019; Kamm et al. 2018; Kayal et al. 2018; Kenny et al. 2020; Jiang et al. 2019; Kim et al. 2022; King et al. 2008; Khalturin et al. 2019; Law et al. 2023; Ledoux et al. 2020; Liang et al. 2025; Libro et al. 2013; Liew et al. 2020; Locatelli and Baums 2025; Macias-Muñoz et al. 2025; Noel et al. 2023; Ohdera et al. 2019; Pootakham et al. 2021; Radice et al. 2025; Robbins et al. 2019; Ryan et al. 2013; Ryu et al. 2016; Salazar et al. 2022; Schnitzler et al. 2024; Schultz et al. 2021; Shinzato, Takeuchi et al. 2021; Shinzato, Khalturin et al. 2021; Simakov et al. 2022; Srivastava et al. 2008, 2010; Stankiewicz et al. 2025; Stephens et al. 2022; Takeuchi et al. 2025; Tang et al. 2025; The Aquatic Symbiosis Project 2026; Vidal-Dupiol et al. 2020; Voolstra et al. 2015, 2017; Wang et al. 2017; Wilding et al. 2020; Wong and Putnam 2022; Xia et al. 2020; Yang et al. 2014; Yoshioka et al. 2022; Young et al. 2024; Yu et al. 2022; Zimmermann et al. 2022) and 71 transcriptomes (Bhattacharya et al. 2016; Burge et al. 2013; Foox et al. 2015; Han et al. 2022; Hasegawa et al. 2016; Kenkel and Bay 2017; Ohdera et al. 2019; Pelosi et al. 2022; Pratlong et al. 2015; Zhang et al. 2019) (Table S1).

For species with only a genome assembly and predicted genes in “gff” format, the program gffread v0.11.6 (“-S -x *cdsfile* -y *pepfile*”) (Pertea and Pertea 2020) was used to infer the predicted protein and CDS sequences. Transcriptomes without predicted proteins available had ORFs predicted using TransDecoder v5.5.0 (https://github.com/TransDecoder/TransDecoder). Candidate ORFs produced by TransDecoder were searched against the Pfam database (release 33.1; i-Evalue < 0.001) using HMMER v3.1b2, and against the SwissProt database (release 2020_05) using BLASTp (v2.10.1; “-max_target_seqs 1 -evalue 1e-5”), with the resulting homology information used by TransDecoder to guide ORF prediction. In genomes and transcriptomes with multiple isoforms predicted per gene only the longest transcript per gene was retained for downstream analysis.

The *Montipora cactus* and *Montipora efflorescens* genome assemblies available from https://marinegenomics.oist.jp (Shinzato et al. 2021) were filtered, keeping only scaffolds identified by Yoshioka et al. 2022 as not being haplotigs; the updated predicted gene models from these authors for *M. cactus* and *M. efflorescens*, and *Astreopora myriophthalma* were used in place of those available from https://marinegenomics.oist.jp. The *Montipora* sp1 aff. *capitata* genome assembly had genes predicted using BRAKER v2.1.6 (Hoff et al. 2016), run using RNA-seq evidence from *M. capitata* aligned by STAR v2.7.8a (Dobin et al. 2013) (see https://zenodo.org/records/14871721 for data and the list of RNA-seq samples used). For the Indonesian *P. acuta* isolate (Vidal-Dupiol et al. 2020), the RNA-seq based “experimental” gene models were used in place of the “ab initio” gene models due to the incompleteness of the latter gene set (as reported by Vidal-Dupiol et al. 2020). The “experimental” genes did not have open reading frames (ORFs) predicted; the longest transcript associated with each gene was extracted (to prevent the prediction of multiple proteins from genes with alternative transcript isoforms) and ORFs were predicted using TransDecoder (following the workflow described previously). Single-exon genes in the Indonesian *P. acuta* isolate without any strand information were assumed to be from the forward/positive strand (TransDecoder will adjust the strand of single-exon genes based on the results of ORF prediction). The full list of genomes and transcriptomes retrieved (as well as the location and version of the data that was downloaded, and any additional processing performed to prepare the data for downstream analysis) is presented in Table S1.

### Quality Assessment of Genome Assemblies and Predicted Genes

The quality of each coral genome assembly and associated predicted genes was assessed using multiple approaches. (i) Quality metrics for the genome assemblies and CDS of the transcriptomes (eg total size, N50, number of sequences, etc.) were calculated using the *stats.sh* script from bbmaps v38.87 (https://sourceforge.net/projects/bbmap/). (ii) Quality metrics of the predicted genes (eg total number, average number of introns/exons, average length, etc.) were calculated by parsing the “CDS” features in the “gff” files available for each set of predicted genes in each genome. Bedtools v2.29.2 (Quinlan and Hall 2010) was used to merge (“-d 1000000000”) and then subtract the extracted “CDS” features from each other, this generates gene (exon + intron) and intron features (respectively) that were used for metric calculation. (iii) BUSCO v5.0.0 (Manni et al. 2021) was run on the genome assemblies (“--offline --mode genome”) and predicted genes (both from the genomes and transcriptome; “--offline --mode protein”) using both the metazoan (metazoa_odb10.2021-02-24) and eukaryota (eukaryota_odb10.2020-09-10) datasets. The full quality metrics from each approach are shown in Tables S1 and summarized in Fig. 1.

The *Acropora hyacinthus, Alatina alata, Craterolophus convolvulus, Favia* sp.*, Henneguya salminicola, Montastraea faveolate, Montipora aequituberculata, Myxobolus cerebralis, Myxobolus pendula, Myxobolus squamalis, Pachycerianthus borealis, Porites astreoides, Renilla reniformis, Stylissa carteri*, and *Thelohanellus kitauei* datasets were excluded from downstream analysis because their protein sets had >50% missing BUSCO genes in the eukaryota_odb10 and metazoa_odb10 datasets. *Pachycerianthus borealis* was included in the removed datasets because it almost had >50% missing BUSCO genes in both datasets (49.8% and 50.1%, respectively). Additionally, one of the two *Cassiopea xamachana* datasets (both derived from the same isolate using different ORF prediction approaches) were discarded as it appeared to have a significant number of proteins originating from redundant isoforms, and one of the two *Calvadosia cruxmelitensis* datasets (both derived from the same isolate; one set derived from the available transcriptome assembly and the other from the available genome assembly) was discarded in favor of the protein set derived from the genome assembly. Additionally, after orthogroup analysis, the *Galaxea fascicularis, Astreopora* sp.*, Montipora cf. grisea, Polypodium hydriforme,* and *Kudoa iwatai* datasets were removed from further analysis by this study (and the constructed orthogroups) due to phylogenetic incongruence (ie they were positioned incorrectly within the constructed phylogeny, suggesting they may have sequence quality issues or have been taxonomically misclassified). A few datasets from the Aquatic Symbiosis Project Data Portal had predicted isoforms that were removed from the orthogroups upon discovery and excluded from further analysis. After quality and phylogenetic filtering, 205 datasets remained for subsequent analysis. Dataset references, quality metric, and taxonomic information are presented in Tables S1, with the removed datasets highlighted red in each table.

### Orthogroup Analysis

Orthogroups (OGs) were constructed from the datasets listed in Table S1 using SonicParanoid2 v2.0.9 (“-m sensitive”). OGs with at least one sequence from each of the included datasets that their protein sequences extracted, aligned using MAFFT v7.453 (“--auto --anysymbol”), trimmed using TrimAL v1.4.1 (“-automated1”), and had phylogenies constructed using IQ-TREE v3.0.1 (“--fast -m WAG”). The constructed phylogenies were used by STAG v1.0.0 (default parameters) to build a consensus species tree with each of the datasets. The species tree produced by STAG was plotted along with associated quality metrics to assist with identification of low quality datasets, using the ggtree v2.6.2 (Yu et al. 2017) and ggplot2 v3.4.2 (Wickham 2016) R v4.2.3 (R Core Team 2021) packages. Phylogenetically incongruent datasets were removed from the constructed OGs and the phylogenetic analysis was rerun to assess the effects of their removal. The final corpus of 205 datasets were used to construct the phylogeny used for all downstream analysis (Fig. 1).

### Protein Functional and Taxonomic Annotation

Functional annotations were assigned to each protein in each dataset using InterProScan v5.53-87.0 (“-dp --goterms”) (Jones et al. 2014), eggnog-mapper v2.1.6 (database retrieved 2021-12-09; “--pfam_realign denovo --report_orthologs --no_file_comments”) (Cantalapiedra et al. 2021; Huerta-Cepas et al. 2019), and a search against NCBI's nr database (version 2022_07) using DIAMOND BLASTP v2.0.15 (“--ultra-sensitive --max-target-seqs 0 --evalue 1e-5 --outfmt 6 qseqid sseqid evalue bitscore staxids”) (Buchfink et al. 2021). It is notable that the DIAMOND search against nr was exhaustive, that is, all hits (not just the top *n* hits, as is the default behavior for all homology search tools) were retained for downstream analysis. This approach allows for analysis of the full sequence similarity information of a given query, which is particularly critical for proteins that are part of large gene families that are shared across many datasets available in NCBI.

A protein was considered to have a putative function if one or more of its DIAMOND hits against the nr database (below the 1e-5 *e*-value threshold) did not have a description containing one of the following (functionally ambiguous) phrases: “uncharacterized protein”, “hypothetical protein”, “predicted protein”, “expressed protein”, or “unnamed protein product”. That is, a protein was considered to have a putative function if at least one of its hits from the exhaustive search against nr had a non-ambiguous description, whereas a protein was considered to have an unknown function if all hits had ambiguous descriptions.

### Orthogroup Classifications

#### Functional and Taxonomic Classification

The OGs produced by SonicParanoid2 were used to identify functionally uncharacterized (“dark”) gene families. An OG was considered functionally uncharacterized if all the proteins that compose it are considered functionally uncharacterized according to the exhaustive DIAMOND search criteria described previously. Conversely, an OG was considered functionally characterized if any of its constituent proteins had a putative function assigned. The taxonomic distribution of each OG within Cnidaria and the outgroups was assessed using a lowest common ancestor approach (LCA, eg if the lowest taxonomic level that captures all proteins in an OG was Scleractinia, then the OG was considered to be Scleractinia-specific). The LCA analysis was performed using the lineage information produced by ncbitax2lin, considering just the following taxonomic ranks from NCBI's Taxonomy Database: tax_id, isolate, species, genus, family, order, class, subphylum, phylum, clade1, kingdom, clade, superkingdom, “no rank.” A custom suborder was manually added with the “Complex” or “Robust” classifications for each dataset from Scleractinia. This analysis was performed using the taxonomic lineage information produced using ncbitax2lin v2.3.2 (https://github.com/zyxue/ncbitax2lin) and the NCBI's “taxdump” file (https://ftp.ncbi.nlm.nih.gov/pub/taxonomy/taxdump.tar.gz) retrieved 8th June 2022.

#### Representative Sequence Selection

To support downstream analysis a single representative sequence was identified for each OG. The representative sequence was identified by re-clustering the proteins in each OG using DIAMOND CLUSTER v2.1.7 (“--evalue 0.001 --approx-id 0 --member-cover 0”), counting the number of times each sequence was identified as a representative in the output (some OGs have multiple “representative” sequences due to the differences in the clustering algorithms used by DIAMOND and SonicParanoid2), with the sequence with the highest count considered the best representative for that OG. If multiple sequences were identified with the same count, then the sequence with its name lexicographically first was chosen and an annotation was added to the OG detailing the multiple possible representative sequences (Table S2; only one of the conserved dark OGs had multiple possible representative sequences).

#### Homology Detection Failure (HDF) Prediction

DIAMOND REALIGN (“--approx-id 0”) was run using the re-clustered proteins to produce a matrix of bitscores between the proteins within each OG. For each OG, the highest bitscores between the representative sequence and each of the datasets in each OG was collected, that is, for each dataset (a genome or transcriptome from a given species) the sequence with the highest bitscore against the representative sequence was selected and used as a representative for that dataset, with the top hit between the OG and dataset representatives used as the bitscore to represent that dataset for downstream analysis. For each OG, the collection of per dataset best bitscores against the representative sequence, along with the length of each dataset's representative sequence, and the phylogenetic distance between each dataset (computed by the APE v5.7-1 (Paradis and Schliep 2019) R v4.3.1 package using the species tree produced by STAG), was used by abSENSE (“--Eval 0 --predall True”; retrieved 18th August 2022) (Weisman et al. 2020) to evaluate if a gene family has arisen via de novo gene origination or accelerated evolution from an ancestral gene family (ie is a dataset missing from the OG due to homology detection failure [HDF] or because a homologous sequence was never present in this dataset). abSENSE predicts, for each dataset present in the phylogenetic distance data, the probability that a homolog from that dataset would be undetected at the specified *e*-value by a homology search tool, even if the homolog was present (ie the proteins in this gene family have/had a fast rate of evolution and thus have potentially diverged in some of the distantly related datasets beyond the point of detection using standard homology detection approaches). The taxonomic classification of each OG (derived from the LCA analysis described previously), was used to group the abSENSE predictions from datasets absent from the OG that are outside the LCA (where we would expect HDF to explain these datasets absence from the OG), and those that are within the LCA (where we would not expect HDF to explain these datasets absence from the OG; possibly absent due to these datasets being incomplete). abSENSE probabilities are scaled between 0 to 1, with a probability of <0.05 (< 5% confidence) considered in our analysis as not supporting HDF as the explanation for the absence of the dataset from the OG, and a probability of > 0.95 (> 95% confidence) considered as supporting HDF as the explanation for the absence of the dataset from the OG. Datasets with abSENSE probabilities ≥0.05 and ≤0.95 were considered ambiguous. The number of non-LCA datasets with support for, against, and ambiguous HDF was counted for each OG and used to support downstream interpretation (Table S2).

### Dark Orthogroup Analysis

#### Selection

Dark OGs that comprised just proteins from the Hexacorallia, Scleractinia, or Cnidaria datasets used in this study, and which contained proteins from ≥50% of the datasets from these groups (eg a Hexacorallia-specific OG needed to contain proteins from ≥50% of the Hexacorallia datasets used in our analysis), were extracted for downstream analysis (Table S2; Fig. 1). The 50% cutoff was applied to exclude OGs that are shared by very few datasets in a taxonomic group (which is challenging to interpret and less informative for the purposes of this research) while still allowing for sufficient flexibility to account for the partial nature of many of the datasets used (ie while many of the datasets had <5% missing BUSCO genes, some had >30% missing genes; Fig. 1). The distribution of genes in each conserved dark OG was visualized (Fig. 1) using ggtree v3.8.2 (Yu et al. 2017), aplot v0.2.2, and ggplot2 v3.4.4 (Wickham 2016).

#### Phylogenetic Analysis

For each conserved dark OG, its proteins were aligned using mafft-linsi v7.453 (Katoh and Standley 2013) and a maximum-likelihood phylogeny constructed using IQ-TREE v1.6.12 (“-m MFP -bb 2000”; that is, using automatic model selection and 2,000 ultrafast bootstraps to assess node support) (Hoang et al. 2018; Kalyaanamoorthy et al. 2017; Nguyen et al. 2015). The resulting maximum-likelihood phylogeny was visualized using ggtree v3.8.2 (Yu et al. 2017).

#### Gene Structure in the Genome

For each conserved dark OG, the expression and structure of the Kāne‘ohe Bay (Hawai‘i) *Montipora capitata* gene models (sample prefix: Montipora_capitata_KBHIv3) were assessed using the chromosome-level genome assembly that is available for this species (Stephens et al. 2022) (Version 3; http://cyanophora.rutgers.edu/montipora/) and the RNA-seq data available from NCBI BioProject PRJNA694677 (Williams et al. 2021). The RNA-seq data from each sample of the BioProject was aligned to the genome using HISAT2 v2.2.1 (“--phred33 --dta --rf --very-sensitive”) (Kim et al. 2019), coordinate sorted by samtools v1.11 (Danecek et al. 2021), and merged by samtools into a single combined BAM file which was visualized with the reference genome and predicted gene models (Version 3; http://cyanophora.rutgers.edu/montipora/) using IGV v2.16.0 (Robinson et al. 2011). The visualization of each gene was used to check that the predicted gene models are expressed and conform to the exon structure of the available expression data, validating that these gene models are in fact real genes in at least one species and not prediction artifacts.

#### HMM-based Homology Search

EggNOG-mapper v2.1.6 (“--pfam_realign denovo --report_orthologs”) (Cantalapiedra et al. 2021; Huerta-Cepas et al. 2019) and InterProScan v5.53-87.0 (“-dp --goterms”) (Blum et al. 2021; Jones et al. 2014) were run on the proteins from each dataset, with the annotations assigned to each protein from our conserved dark OGs extracted to assess if HMM-based prediction tools can provide insight into the putative structure or function of these sequences. For the proteins in each conserved dark OG, the EggNOG-mapper predictions were parsed to remove annotation with blank “Description,” “GOs,” and “KEGG_ko” columns (ie annotations without any described functional detail), keeping just the annotations with some form of description. For the proteins in each conserved dark OG, the InterProScan predictions were parsed to separate “structural” (those from the “MobiDBLite,” “Coils,” “Phobius,” and “TMHMM” tools) and “functional” (all other tools) annotations. The functional annotations were merged into putative functional regions using bedtools (v2.29.2; the “sort” and “merge” commands). The TMHMM predictions were merged and subtracted from the functional regions (“bedtools subtract”), keeping only the regions that remained >30 bp in length. This produced a set of putatively functional regions that are not predicted to also be transmembrane spanning.

#### Prediction of Protein 3D Structure and Comparison to Available Databases

The 3D structure of each representative protein from each conserved dark OG was predicted using AlphaFold v2.3.1 (“--db_preset = full_dbs --nouse_gpu_relax --max_template_date = 2023-05-14 --model_preset = monomer --db_preset = full_dbs”; databases retrieved 12th May 2023) (Jumper et al. 2021), with the resulting structure visualized using the r3dmol v0.2.0 R package (Rego and Koes 2015). The resulting structures were classified as having either very low (pLDDT < 50), low (≥50 and <70), confident/high (≥70 and <90), or very high (≥90) prediction confidence based on the average pLDDT quality values across the whole protein.

Each of the representative predicted structures were compared against the available structures from PDB (2023-05-10) and Alphafold-UniProt50 (v4) by Foldseek (retrieved from https://github.com/steineggerlab/foldseek on 23/5/15; “easy-search --format-output query,target,fident,alnlen,mismatch,gapopen,qstart,qend,tstart,tend,evalue,bits,qlen,tlen,theader,taxid,taxname,taxlineage”) in order to identify putative remote homology. Hits were also removed if they had an *e*-value ≥ 0.01. Structural alignments and analysis were carried out using UCSF ChimeraX.

#### Thermal and pH Stress Expression Analysis

An initial analysis of thermal and pH stress responsiveness of the *Montipora capitata* (sample name: Montipora_capitata_KBHIv3) and *Pocillopora acuta* (Pocillopora_acuta_KBHIv2) genes in each of the conserved dark OGs was conducted using the RNA-seq data from the SRA BioProjects PRJNA694677 (Williams et al. 2021) (*M. capitata*) and PRJNA731596 (Stephens et al. 2023) (*M. capitata* and *P. acuta*), and the proteomic data from MassIVE ID MSV000088443 (Williams et al. 2023) (*M. capitata*). This resulted in a total of three transcriptome (two from *M. capitata* and one from *P. acuta*) and one proteomic (*M. capitata*) datasets which were used to identify if a conserved dark ortholog may have a stress response function. Expression analysis of Montipora_capitata_KBHIv3 was conducted using a sequence database consisting of (i) predicted genes from the Montipora_capitata_KBHIv3 nuclear genome assemblies (Stephens et al. 2022); (ii) predicted genes from the *Cladocopium goreaui* nuclear genome assembly (Chen et al. 2022); (iii) predicted genes from the *Durusdinium trenchii* CCMP2556 nuclear genome assembly (Dougan et al. 2022); (iv) predicted genes from the *Montipora capitata* (OX421459.1) mitochondrial genome assembly; (v) predicted genes from the *Breviolum minutum* mitochondrial genome assembly (Shoguchi et al. 2015); and (vi) predicted genes from the *Cladocopium* C3 plastid genome assembly (Barbrook et al. 2014). Expression analysis of Pocillopora_acuta_KBHIv2 was conducted using a database which consisted of (i) predicted genes from the Pocillopora_acuta_KBHIv2 nuclear genome assemblies (Stephens et al. 2022); (ii) predicted genes from the *Cladocopium goreaui* nuclear genome assembly (Chen et al. 2022); (iii) predicted genes from the *Durusdinium trenchii* CCMP2556 nuclear genome assembly (Dougan et al. 2022); (iv) predicted genes from the *Pocillopora damicornis* (Flot and Tillier 2007) (EF526302) mitochondrial genome assembly; (v) predicted genes from the *Breviolum minutum* mitochondrial genome assembly (Shoguchi et al. 2015); and (vi) predicted genes from the *Cladocopium* C3 plastid genome assembly (Barbrook et al. 2014). For both datasets, a database of predicted protein sequences and a database of predicted CDS sequences was produced to support the proteomic and transcriptomic analyses. The “metatranscriptomic” and “metaproteomic” datasets were used to ensure that data from the algal symbionts were being correctly accounted for in the analysis, that is, to ensure that the algal data was not being erroneously attributed to coral transcripts or proteins.

The *Montipora capitata* RNA-seq samples from PRJNA694677 were trimmed for low quality bases and adapters using Trimmomatic v0.38 (Bolger et al. 2014), with gene expression quantified by Salmon v1.8.0 (“--validateMappings --seqBias --gcBias”) (Patro et al. 2017) using just the read pairs where both mates survived. The *Montipora capitata* and *Pocillopora acuta* RNA-seq samples from PRJNA731596 were trimmed for adapters and low-quality regions using Cutadapt v2.9 (Martin 2011) (“--nextseq-trim 10 --minimum-length 25 -a AGATCGGAAGAGCACACGTCTGAACTCCAGTCAC -A AGATCGGAAGAGCGTCGTGTAGGGAAAGAGTGTA”). A second round of trimming with Cutadapt, using the output from the first round, was used to remove poly-G regions from the 5′ ends of the second read in each pair (“-G G{20} -e 0.0 -n 10 --minimum-length 25”). Gene expression was quantified for each sample across the two species datasets using Salmon v1.8.0 (“--validateMappings --seqBias --gcBias”). For each of the three transcriptomic datasets, transcripts with low or zero expression were removed from our analysis using the following filtering criteria: only transcripts with an aligned (by Salmon) read count >10 in > 10% of samples (performed using the *pOverA* function from the genefilter v1.76 R package) were kept for differentially expression analysis using the DESeq2 v1.34.0 (Love et al. 2014) R package. In each dataset, DESeq2 was used to identify differentially expressed genes (DEGs; adjusted *P*-value < 0.05 and an absolute log_2_ fold-change [FC] > 0.5) between the temperature treatments at each time point; between time point comparisons were not considered for this analysis due to the high number of possible combinations of comparisons that this would produce. The transcripts per million normalized expression values produced by Salmon were used for downstream visualization (Dataset S1).

For each RNA-seq dataset, the proportion of dark and light genes with significant FC (> 0.5) and adjusted *P*-values (<0.05) in each pair of conditions analyzed, out of the total number of each group of genes with significance across any pair of conditions, were identified and used to assess if either group is over represented in a particular set of comparisons (Dataset S1).

The proteomic data from MassIVE ID MSV000088443 was reanalyzed using Proteome Discover v2.4 (identical parameters as Williams et al. 2023) and the *M. capitata* metaproteomic dataset. Base R (v4.3.2) and the MixOmics (v6.3.2) R packages were used to calculate FC and *P*-values (Welch *t* test) between the control and treatment samples at each time point using the normalized protein intensity values. Differentially accumulated proteins were defined as having an absolute FC > 0.5 and a *P*-value < 0.05.

#### Cell Type-specific Expression Analysis

The cell type specific expression patterns of the genes in each conserved dark OG were assessed using the *Stylophora pistillata* (Levy et al. 2021), *Xenia* sp. (Hu et al. 2020), *Nematostella vectensis* (Sebé-Pedrós et al. 2018), and *Hydra vulgaris* (Siebert et al. 2019) single-cell RNA-seq datasets. For each species, the data used for this analysis were retrieved from Levy et al. 2021 (Levy et al. 2021), not the original publication. The *Xenia* sp. scRNA-seq data uses the same gene IDs as our dataset, enabling its direct integration into our analysis. The other three species with available scRNA-seq data use older or custom versions of the species reference data, and thus, the gene IDs that were used need to be linked with their versions in the updated datasets which we used in our analysis. For each of these three species, the genes used for the scRNA analysis were retrieved and compared, using a reciprocal BLASTP v2.13.0 analysis, against the proteins from the datasets used in our analysis. For each species, only protein pairs that represent reciprocal best BLAST hits between the old and new datasets, and which had >60% query or subject coverage, were used to transfer the scRNA-seq results between the old and new datasets. Genes were considered to have significant expression in a given cell type if they had a reported FC > 2.0. For each scRNA-seq dataset, at each level of cell type granularity (that is, “metacell,” “cell,” or “broadcell,” if available), the proportion of dark and light genes with significant FC (> 2.0) in each cell type, out of the total number of each group of genes with significance in any cell type, were identified and used to assess if either group is over represented in a particular cell type (Dataset S1). The “metacell,” “cell,” and “broadcell” types were predicted and assigned putative titles by their respective studies; “broadcell” types define single cells at their most high-level classification (eg neurons vs. muscle cells), “cell” types define single cells at a more granular level potentially tissue-subtype level (eg muscle cell vs. muscle stem cell), and “metacell” define single cells at the most granular level (without assigning them names, just groups under a broadcell type).

### Orthogroup Remote Homology

For each OG, sequences were extracted, had terminal stop codons (“*” characters) removed, and internal stop codons replaced with “X” characters. The cleaned proteins from each OG were aligned using mafft v7.453 (“--auto --anysymbol”). Note, OGs with two or more sequences were aligned using mafft, whereas sequences with just a single sequence were included as is. HH-suite v3.3.0 (commit: 4c0cd66434ce0b83ccd247053f57989fdd53d82b; following https://github.com/soedinglab/hh-suite/wiki#building-customized-databases) (Steinegger et al. 2019) was used to combine the alignment into a database using the ffindex_build (default params) tool, had consensus sequences calculated using the hhconsensus (“-M 50 -maxres 200000”) tool, had HMM's built using the hhmake (“-maxres 200000”) tool, had context states calculated for prefiltering using the cstranslate (“-f -x 0.3 -c 4 -I a3m”) tool, and were sorted using the ffindex_order (default params) tool. The final HH-Suite database, which included all OGs, was compared against itself in an all-versus-all manner using the hhblits (“-n 2 -Z 1000000000 -B 1000000000 -e 1 -v 1 -maxres 200000 -maxfilt 1000000000”) tool. The hhblits results were filtered, keeping just hits with probability ≥ 50%, query coverage ≥ 60%, and subject coverage ≥ 60%, with a normalized length score computed for each hit by dividing the alignment score by the alignment length. Self-hits were removed; for each pair of OGs, the best hit with the highest length normalized score was retained, removing lower scoring hits or redundant hits (that is, hits from query to source and source to query that cover the same region). The final set of filtered, non-redundant hits were loaded into a network, treating the OGs as nodes and hits as edges, using graph-tool v2.55 (https://graph-tool.skewed.de/) (Peixoto 2014). Graph-tool was used to load the edges into a network, compute the connected components, compute the SFDP layout (default parameters), and plot the resulting network layout with nodes (OGs) colored according to their Dark/Light designation, presence of genes with significant expression in the stress or scRNA-seq datasets, LCA assigned taxonomic level, or LCA assigned taxa (Fig. 4; scripts available from https://github.com/TimothyStephens/Network_Analysis_Scripts).

### Light Gene Structure Comparison

The structure of selected light genes was used to assess the accuracy and performance of AF2 on sequences from the diverse taxa included in this study. Genes which were classified as “complete” by the previously described BUSCO eukaryota_odb10 analysis of the *M. capitata* KBHIv3 predicted proteins were extracted and used as our representative set of light sequences. *M. capitata* KBHIv3 dataset was selected as the basis for this analysis because it has a well assembled and highly complete reference genome and predicted gene set. Complete BUSCO genes identified using the eukaryota_odb10 lineage dataset were used for this analysis (over, for example, representative light OGs selected manually or using some other metrics) as they are the epitome of well conserved, clearly define gene families, which will undoubtably have verified structures available in PDB. The selected *M. capitata* KBHIv3 proteins had their structures predicted using ColabFold v1.5.5 (Mirdita et al. 2022) (“colabfold_search” [default params] and “colabfold_batch” [“--amber”]). The resulting structures were compared against PDB (retrieved 2023-05-12) using FoldSeek v8.ef4e960 (van Kempen et al. 2024) (“easy-search --format-mode 4 --format-output query,target,fident,alnlen,mismatch,gapopen,qstart,qend,tstart,tend,evalue,bits,qlen,tlen,qtmscore,ttmscore,alntmscore,rmsd,prob,taxid,taxname,taxlineage”), with just the top structural alignment retained per query (Table S3). The function of each of the selected light proteins was inferred using a DIAMOND BLASTP v2.1.8 search (“--ultra-sensitive --evalue 1e-5 --max-target-seqs 200 --outfmt 6 qseqid sseqid pident length mismatch gapopen qstart qend sstart send evalue bitscore qlen slen stitle”) against the SwissProt (version 2022_02) database, retaining just the top hit per query (Table S3).

## Supplementary Material

evag072_Supplementary_Data

## Data Availability

Add data required for the interpretation of this research is available in the Supplemental Information, including a list of databases and database IDs used during retrieval of the genome and transcriptome data used in this study. The code for network analysis using graph-tools is available from https://github.com/TimothyStephens/Network_Analysis_Scripts. The code for genome and transcriptome processing is available from https://github.com/TimothyStephens/0031_Coral_genomes and the code for the general analysis performed by this study is available from https://github.com/TimothyStephens/0032_Coral_genomes_analysis. The final orthogroups constructed in this study (annotated as dark or light with associated taxonomic and other relevant information) are available from https://doi.org/10.5281/zenodo.14871721.
