# Supplementary material for: Cosmopolitan Gene Families With Known Functions Are Hotspots for the Evolution of Novel Genes in Stony Corals
Source: Genome Biol Evol. 2026 Mar 24;18(4):evag072. doi: 10.1093/gbe/evag072 (PMC13044578; doi:10.1093/gbe/evag072)
Supplement: evag072_Supplementary_Data [file evag072_supplementary_data.zip › Stephens_etal_2026_Supplemental_Text_R3.docx]

**Supplemental text**

**Orthogroup analysis and dark gene classification**

A total of 7,137,414 proteins, from the 205 datasets, were clustered into 793,054 orthogroups (OGs). Of these, 186,624 (23.53%) comprised multiple sequences and 606,430 (76.47%) were singletons. Of the multi-sequence OGs, 185,960 (99.64%) comprised sequences from multiple datasets and 664 (0.36%) were from a single dataset. Of the 793,054 constructed OGs, 502,047 (63.3%) were classified as Dark (functionally uncharacterized; comprising 813,610 proteins [11.4%]) and 291,007 (36.7%) were classified as Light (putatively functional characterized; 6,323,804 proteins [88.6%]) using our conservative dark gene annotation approach (see methods for additional details). While more OGs were classified as Dark, they comprised fewer sequences (439,612 [87.56%] of the 502,047 Dark OGs are singletons) than the Light OGs, suggesting that Dark OGs are smaller and likely more linage-restricted than Light OGs. The distribution of sequences in each OG class, based on the number of exons per gene (**Figure S1A**) or protein length (**Figure S1B**), are comparable: i.e., dark OGs have very similar distributions of exon counts and protein lengths compared to light OGs. The only major difference is that light OGs have more proteins with large exon counts and lengths (shown by the increased densities at the x-axis maximum in **Figures S1A**, **S1B**).

**Expression patterns of dark genes across Cnidaria**

Across all three RNA-seq datasets (**Dataset S1**), the proportion of light and dark genes with significant differential expression (FC > 0.5 and adjusted *p*-value < 0.05) in one or multiple treatment conditions, was higher for the former then the latter. This suggests that light genes are more likely to be involved in the transcriptional response of *M. capitata* and *P. acuta* under the analyzed conditions (thermal and pH stress over short [weeks] and long [months] timeframes). The proportion of dark and light genes identified as being significantly differentially expressed between each pair of treatments was roughly congruent: i.e., treatment comparisons with a high proportion of light genes also tended to have a high proportion of dark genes (out of all light and dark genes with significant differential expression). There were however some notable exceptions, particularly in the *M. capitata* 12TP dataset, in which the proportion of light genes was higher than dark (ATAC9vsATHC9) and dark higher than light (ATAC8vsHTAC8).

In the scRNA-seq datasets, the proportion of genes with significant (FC > 2) expression was higher for light than dark genes, with the only exception being *H. vulgaris*. Calicoblast cells (which secrete the calcareous coral exoskeleton) were only present in the *S. pistillata* adult and polyp datasets, but in both cases had higher proportions of dark genes at all levels of cell type granularity (barring one or two cell types at the “metacell” level). In addition, a higher proportion of dark genes was observed across most datasets in cell types (particularly broad cell types) annotated as gastrodermis (except *N. vectensis*), epidermis (except *Xenia* sp.), neuron (although usually only at more granular cell type level). Interestingly, dark genes are also present in a higher proportion in cnidocyte cell types from *N. vectensis* and *H. vulgaris*, with the latter also having the most cnidocyte “cell” and “metacell” types identified across all datasets. In each of the examples described above, there are exceptions at more granular cell type levels. For example, there is a higher proportion of dark genes with significant FC in the *H. vulgaris* cnidocyte “broad” cell type. In the “cell” and “metacell” types some of the identified groups of cells have higher proportions of light genes, with the majority however, having higher proportions of dark genes. Conversely, in *H. vulgaris* at all levels of granularity (i.e., “broad”, “cell”, and “metacell”, excluding one “metacell” type), gastrodermis cells have a higher proportion of dark genes. Similar patterns are observed across different cell types in each of the other datasets. It should be noted that in all cell types, the number of light genes with significant expression in the RNA-seq and scRNA-seq datasets was always much higher than for dark genes. Whereas this is expected given that most proteins required for cellular functions are well conserved across a diverse range of species, it does conflict with the null hypothesis, suggesting that the distribution of dark genes in this analysis shows a bias towards certain conditions or cell types.

**Selection of conserved dark OGs**

Dark OGs that were taxonomically restricted to datasets from Hexacorallia, Scleractinia, or Cnidaria, were selected for downstream analysis if they contained proteins from ≥50% of the datasets from these groups (e.g., a Hexacorallia-specific OG needed to contain proteins from ≥50% of the Hexacorallia datasets used in our analysis; **Tables S2; Figure 1**). Each of these conserved dark OGs were subjected to phylogenetic, protein structure, HMM-based functional, and gene expression analyses to aid their characterization in corals and identify potential hot spots for functional innovation. There were 244 dark OGs which satisfy the above criteria, 30 for Cnidaria, 198 for Hexacorallia, and 16 for Scleractinia. Overall, 138 of the 244 conserved OGs (56.56%) had perfect, or near perfect congruence (agreement) across at least one (if multiple were present) of the predicted gene models in *M. capitata* (KBHIv3) and the aligned RNA-seq data (i.e., the aligned RNA-seq data supported the exon/intron patterns of each gene model), strongly supporting their transcription. In addition, 25 (10.25%) OGs had one or multiple problematic exons (usually located at the 5-prime termini), but otherwise had good congruence across the remaining exons and good conservation across the other genes in the OG. Only 16 (6.56%) OGs were missing a homolog in *M. capitata* and could not have their exon structures validated, and 41 (16.8%) had no aligned reads to support the expression of these genes in the *M. capitata* RNA-seq samples used. Finally, 24 (9.84%) had gene structures that were completely incongruent with the aligned RNA-seq data. In each of these later two cases however, the *M. capitata* proteins had strong sequence similarity with other proteins in the OG, suggesting that, although these genes do not have strong transcriptome support in the dataset used for this analysis, they are conserved across other datasets and likely to be functional. The phylogeny constructed for each of the conserved dark OGs generally shows strong congruence with the STAG species tree. For example, the Cnidarian Dark orthogroup OG000011274 (**Figure 2**) has high sequences conservation and congruence between the major taxonomic lineages, with only minor variation observed for sequences that are incomplete or mis-predicted (e.g., the *Alatina alata* sequence at the bottom of the tree).

**3D protein structure of conserved dark OGs**

AlphaFold2 was used to predict the 3D structure of each conserved OG representative sequence, producing 70 very low (28.69% of 244 total conserved OGs), 141 low (57.79%), 32 confident (13.11%), and 1 very high (0.41%) confidence structures (see methods for the range of pLDDT values that constitute each confidence internal). An example of a high confidence structure is shown in **Figure 3C**. Many of the confident and very high confidence structures have core structures that are well resolved but have long P- or N-terminal or internal loop regions that are less well resolved, lowering the overall score. Examples of this phenomenon are shown in **Dataset S3**, with in all cases, the representative sequence chosen for 3D structure prediction having high sequence conservation with the other members of the OG over most of its length, precluding gene mis-prediction as the cause of the low confidence structures.

Of these conserved dark OGs, 161 (65.98%) had functional domains assigned by EggNOG-mapper or InterProScan, transmembrane regions identified by TMHMM (part of InterProScan), or 3D structural similarity (predicted by Foldseek) to other proteins in PDB or the AlphaFold-UniProt database (**Figure S2**). Of these, 115 (71.43% of 161; **Figure S2**) had TMHMM annotations, with 58 having just TMHMM annotations and 57 having annotations from multiple sources. Only 17 OGs had domains identified only by EggNOG-mapper or InterProScan, and 28 had Foldseek or Foldseek and domain annotations. Therefore, of the 161 OGs with annotations assigned, 115 (71.43%) are putatively membrane associated and 46 (28.57%) are putatively not transmembrane associated, possibly functioning in other cellular processes such as metabolism or substrate binding. It should be noted that the EggNOG-mapper and InterProScan “functional” domains were not partitioned into those that represent enzymatic functions, those that are more structural (e.g., repeat domains), and those that are Domains of Unknown Function (DUF domains).

**Selection of AF2 Structures for Manual Analysis**

Five well resolved AF2 structures were selected for comparison against crystal and cryo-EM structures deposited to the RCSB Protein Data Bank (PDB) (Dataset S2). These five structures were selected from the initial version of our analysis which used 120 datasets available at the time this study was started (January 2023). Since this analysis was first conducted, significantly more datasets have become available (as of November 2025), with the current version of this manuscript using 205 datasets. The five AF2 structures selected were chosen from the conserved dark OGs produced from this initial version of our analysis (using the 120 datasets); they were specifically chosen from all other conserved dark OGs because they had Confident (≥ 70) AF2 structures, were relatively long, and had good sequence conservation between the other proteins in their respective OGs. The structures were derived from the representative sequences of the five conserved dark OGs. In the current version of this manuscript, with the addition of new datasets, the five original OGs are still represented but have new sequences added and different representative sequences (as expected with the additional of new datasets). Of the five OGs identified in the current analysis, three (OG000009717, OG000010602, and OG000011843) were in our set of conserved dark OGs, while two (OG000001644 and OG000008812) were not present in the conserved dark OGs because one and two of the proteins (respectively) from the new datasets had putative annotations that resulted in these OGs being classified as light. However, it should be noted that each of these OGs had 397 and 194 proteins (0.25% and 1%, respectively), and the sequences with annotations were outliers in respect to their lengths (shorter or longer than other sequences in their OG), thus the classification of these OGs as light in our new analysis is due to the highly conservative nature of our annotation approach and represents false negative classifications (see Methods and Study Limitations sections for further discussion). Given the significant insights that this analysis has yielded, and our confidence that these five structures still represent dark gene families of interest in Cnidaria or Hexacorallia, we have chosen to continue using these structures for this analysis in the current version of the manuscript.

**3D protein structure of light genes**

The performance of AlphaFold2 with coral proteins was assessed to ascertain if the large number of poorly resolved conserved dark OGs (**Table S2**) is due to them having novel structures, or because these tools are unable to correctly resolve structures in divergent species. Of the 243 *M. capitata* KBHIv3 proteins identified as being “complete” according to BUSCO analysis using the eukaryota_odb10 lineage dataset, all but 26 (10.7%; **Table S3**) had structures with an average confidence of high (164; 67.49%) or very high (53; 21.81%). The top FoldSeek structural alignment against the PDB database generally, had very high alignment TM-scores, with 91 (37.45%; **Table S3**) proteins with TM-scores > 0.9 and 222 with a TM-score > 0.5 (91.36%). Most of the structural alignments covered nearly the full-length of both the query and target proteins, with only 72 of the proteins with < 70% query coverage and 11 with < 70% target coverage, demonstrating that the TM-scores are representative of the whole protein and not just a conserved subregion.

**Study limitations**

The approach used by this study to identify dark genes is highly conservative and expected to underestimate their total abundance. Considering all genes in an OG to be functionally annotated if a single member has a hit to a protein with a described function in the NCBI nr database, will lead to false positive light OG classifications (see “Selection of AF2 Structures for Manual Analysis” for two examples where this was observed to occur). Proteins in the NCBI nr database with inaccurate annotations, or proteins in our database that have been mis-predicted to erroneously include regions of other (potentially functionally characterized) proteins, will result in OGs being incorrectly assigned as functional. This means that the dark OGs identified in our study represent a conservative high-quality set. There is also a clear bias in the number of datasets available from each of the major taxonomic lineages, with many more datasets available from specific lineages within Scleractinia compared to all other groups. This bias limits our ability to fully assess the role that dark gene families have played in the evolution of the more sparsely sampled lineages.

Annotation heterogeneity, that is, the use of different prediction tools to infer genes in different datasets, may also be a major source of the lineage (or in our case dataset) specific dark genes that we have observed (Chen et al. 2020; Weisman et al. 2022). However, given that our study does not focus on dark genes found in just a single dataset, but rather on well conserved gene families or clusters of proteins with remote homology, we do not expect this issue to significantly impact our results. That is, any erroneous or malformed genes produced by old or inconsistent gene prediction approaches will likely be singletons (with no or weak similarity to other genes in our datasets), thus they will be ignored by our downstream analysis. It has been well documented that gene prediction tools make consistent mistakes (i.e., they will predict the same erroneous genes) in related datasets. While this has the potential to produce erroneous orthogroups, given the diversity of gene prediction approaches used across all the studies that contributed datasets to our analysis, it is unlikely that this would be a pervasive issue and is unlikely to have produced large erroneous orthogroups that might affect our results. While this has the greatest potential to affect our remote homology analysis and may explain some of the low taxonomic level OGs that we observe, it does not explain why the cluster with higher taxonomic OGs with known functions, or why the network has such defined structure; erroneous genes would be expected to have no or little homology with true genes, preventing them from forming part of our network analysis.

The 3D structures produced for the conserved dark OGs require additional validation by approaches such as x-ray crystallography. The accuracy of AlphaFold2 with novel gene families in corals is yet to be validated, however, the wealth of high-quality candidates produced by this study should provide a fruitful pool from which this limitation can be addressed.

Supplemental Figure Titles and Legends

**Figure S1. Comparison of sequence composition between different types of OGs.**

Ridgeline plots comparing the (A) number of exons per gene and (B) protein length distributions of genes in OGs of each designation. Ridgeline plots comparing the (C) number of exons per gene and (D) protein length distributions of genes in conserved vs non-conserved dark OGs. To improve readability, the maximum x-axis value show in each plot (i.e., 20 for A and C, and 1000 for B and D) represents the cumulative density of data greater than that value. (E, F) 2D density plots of conserved OGs using the proportion of datasets outside of the focal group (i.e., Scleractinia, Hexacorallia, or Cnidaria) with homology detection failure (HDF) probabilities < 5% (no evidence of HDF; y-axis) or > 95% (evidence of HDF; x-axis). The plot is show (E) with and (F) without the values of individual OGs overlayed as pink dots. Colors used for the density plots are shown at the bottom of the figure.

**Figure S2. Conserved dark OG annotations.**

Upset plot showing the number of conserved dark OGs with assigned transmembrane (TMHMM) annotations, functional (EggNOG-mapper or InterProScan) annotations, or 3D structure similarity; the latter was assessed using a FoldSeek (*e*-value <0.01) search against structures in the PDB (2023-05-10) and Alphafold-UniProt50 (v4) databases. Sets of OGs in the plot with transmembrane annotations are colored blue, sets of OGs without transmembrane annotations but with FoldSeek hits are colored green, and sets of OGs with just functional annotations are colored orange. The number of OGs in each set is shown above each bar in the plot.

**Figure S3. Predicted homodimer 3D structure of the OG000008812 representative sequence.**

The predicted lDDT and aligned error plots produced by ColabFold are shown below the structure.

Supplementary data Description

**Dataset S1. Accumulation patterns of dark and light genes across stress and single-cell RNA-seq datasets.**

(**Pages 2-4**) Proportion of dark (purple bars) and light (orange bars) genes with significant (adjusted *p*-value <0.05 and an absolute log_2_ fold-change [FC] >0.5) expression at each time point in the BioProjects PRJNA694677 (Williams et al. 2021) (*M. capitata* 3TP) and PRJNA731596 (Stephens et al. 2023) (*M. capitata* 12TP and *P. acuta* 12TP) RNA-seq datasets. The absolute number of significantly expressed genes (between the control and pH or temperature treatment conditions) is displayed above each bar. For each pair of treatment comparisons (the “Expression Results” panel), the y-axis represents the percentage of significant DEGs of each type (dark or light), out of all significantly DEGs across all comparisons. For the “Total” bars, the y-axis represents the percentage of significant DEGs of each type (dark or light), out of all genes in each species. (**Pages 6-11**) Proportion of dark (purple bars) and light (orange bars) genes with significant (fold-change [FC] > 2) expression in each “metacell”, “cell”, and “broadcell” type in the *Stylophora pistillata* (Levy et al. 2021), *Xenia* sp. (Hu et al. 2020), *Nematostella vectensis* (Sebé-Pedrós et al. 2018), and *Hydra vulgaris* (Siebert et al. 2019) single-cell RNA-seq datasets scRNA-seq datasets. Bars are not stacked but overlayed on top of each other, with the smaller bar being placed on top of the larger for clarity. Bars at the “metacell” and “cell” levels are grouped under their “broadcell” types in each figure; the “broadcell” types used to group each set of bars are shown in colored boxes at the top of each figure. The absolute number of significantly expressed genes in each cell type is displayed above each bar. For each cell type, the y-axis represents the percentage of significantly expressed genes of each type (dark or light), out of all significantly expressed genes across all cell types. For the “Total” bars, the y-axis represents the percentage of significantly expressed genes of each type (dark or light), out of all genes in each species.

**Dataset S2. Structural comparison of five conserved OGs against crystal structures from PDB.**

**(Page 1)** Ependymin-related blue carotenoprotein is responsible for the coloration of the marine blue sponge *Haliclona* sp. The X-ray structure of EPD-BCP (PDB ID: 8i34) (Kawasaki et al. 2023) showing the heterodimeric protein composed of two, α and β, subunits (both displayed in gray) with two carotenoids (astaxanthin, AXT and mytiloxanthin, MXT, shown in blue) bound at the inter-subunit interface superposed onto the monomeric AF2 model (OG000001644, shown in orange) composed of two domains connected by a linker (**Panel A**). The AF2 model and the β subunit of EPD-BCP reveal a significant structural homology with Root Mean Square Deviations (RMSDs) ranging from 1 Å to 5 Å across the entire homologous parts of the proteins including the ligand-binding surface in the β subunit (**Panel A**), and 23% sequence identity (**Panel F**). The accuracy of AF2 prediction is reflected by high Predicted Local Distance Difference Test (pLDDT) values (i.e., >90) across the entire model except an inter-domain linker and a short α-helix in the C-terminal domain (**Panel C**). Structural details of EPD-BCP and the AF2 model are displayed (**Panels D and E**, respectively). AXT and MXT (displayed in blue) engage in specific interactions with the residues lining the outer face of the *β*-sheet in the β subunit of EPD-BCP, for example I40, Y142 and F144. The AF2 model displays a similar arrangement of residues (e.g., I168, F275 and Y279, respectively) located in analogous positions. The above structural analysis collectively suggests that the coral protein may also function as a dimer (**Panel E**). Sequence alignment of EPD-BCP and the AF2 model (OG000001644) (**Panel F**). The top row in the figure indicates similarities in the C_α_ alignment. **(Page 2)** Nitrophorin 4 (Np4) is a heme-containing protein that binds nitrate. The X-ray structure of Np4 (PDB ID: 3mvf) (He et al. 2010) revealed a *β*–barrel fold coordinating two ligands i.e., a heme and a molecule of nitric dioxide (NO_2_^-^). The AF2 model (OG000010602) and Np4 display a significant structural homology (**Panel A**), with RMSDs ranging from 1 Å to 5 Å in the region corresponding to the ligand-binding pocket (**Panel B**). However, both proteins display only a moderate sequence identity (~17.1%; **Panel F**). The accuracy of AF2 predictions is reflected by high pLDDT values (i.e., >90) across residues comprising the entire *β*-barrel (**Panel C**). **Panel D** shows structural details of Np4 with a conserved H59 that covalently binds a heme molecule which, in turn, coordinates NO_2_^-^. An analogous region from the AF2 model contains H157, possibly indicating functional similarities (**Panel E**). **(Pages 3 and 4)** Chondroitin B lyase (ChonB) (Michel et al. 2004) is involved in dermatan sulfate metabolism. ChonB catalyzes the enzymatic cleavage of the β(1,4) linkage in dermatan sulfate oligosaccharides yielding 4,5-unsaturated disaccharides. The crystal structure of ChonB bound to several dermatan sulfate oligosaccharides revealed the presence of a calcium ion in its active site coordinated by N213, E243 and E245. The AF2 model (OG000009717) and ChonB (PDB ID: 1ofl) (Michel et al. 2004) display a significant structural homology (**Panel A**) and sequence similarity (**Panel F**) in its C-terminal β-sheet with RMSDs ranging from 1 Å to 5 Å, particularly in the region corresponding to the active site of ChonB (**Panel B**). The accuracy of AF2 predictions is reflected by high pLDDT values (i.e., >90) across the β-sheet (**Panel C**). **Panel D** displays structural details of ChonB. Conserved residues involved in coordination of a calcium ion are highlighted in the inset. Several residues from an analogous region in the AF2 model are highlighted in **Panel E**. **(Page 5)** Superkiller 8 (Ski8) is a regulatory protein that assembles into a complex directing cytoplasmic mRNAs toward the nucleolytic exosome for degradation (Keidel et al. 2023). The cryo-EM structure of Ski238-Ski7-exosome assembly (PDB ID: 8qca) (Keidel et al. 2023) unveiled that Ski8 adopts a circular shape composed of *β*-sheets, and it anchors Ski2 on Ski3. The AF2 model (OG000008812) and Ski8 reveal a significant structural homology (**Panel A**) with RMSDs ranging from 1 Å to 5 Å across most of the structure (**Panel A and Figure S3**), and 22% sequence identity (**Panel F**). The accuracy of AF2 predictions is reflected by high pLDDT values (i.e., >90) across the β-sheet (**Panel C**). Structural details of Ski8 and AF2 model are displayed in **Panel D** and **E**, respectively. **(Page 6)** Ml115 is a ribosomal protein associated with the mitochondrial large ribosomal subunit. The cryo-EM structure of ml115 (PDB ID: 7pkt) (Waltz et al. 2021) revealed that ml115 is predominantly α helical. The AF2 model (OG000011843) and ml115 reveal a significant structural homology (**Panel B**) with RMSDs ranging from 1 Å to 5 Å across a C-terminal part of the molecule (**Panel A**), and ~19.5% sequence identity (**Panel F**). The accuracy of AF2 predictions is reflected by high pLDDT values (i.e., >90) across the entire AF2 model, except for its C-terminal α-helix (**Panel C**). Structural details of EPD-BCP and the AF2 model are displayed in **Panel D** and **E**, respectively.

**Dataset S3. Phylogeny, structure, and expression data for a subset of conserved dark OGs.**

(**Page 1**) Phylogeny, structure, and expression of dark genes in the Hexacorallia Dark orthogroup OG000008521. (A) ML phylogeny of the protein sequences in the OG, with associated alignment shown on the right. Nodes with > 95% bootstrap support are annotated with blue circles, the sequence chosen as representative for the OG is highlighted with red text, sequences from *M. capitata* KBHIv3 with blue text, *P. acuta* KBHIv2 with green text, and *S. pistillata* GAJOv1 in purple text. (B) AF2 structure of the OG reference sequence. Relative expression of the one *S. pistillata* gene in the OG in (C) adult, (D) polyp, and (E) larva “cell” types. The > 2.0 FC cutoff used to signify significant cell type specific expression in this study is represented by a horizontal dashed red line in plots C-E. (F) Differential gene expression results for the one *P. acuta* gene in the OG. Comparisons with significant (FC > 0.5 and adjusted *p*-value < 0.05) differential expression are represented by colored horizontal lines and text describing the FC and adjusted *p*-value. (**Page 2**) Phylogeny, structure, and expression of dark genes in the Hexacorallia Dark orthogroup OG000011849. (A) ML phylogeny of the protein sequences in the OG, with associated alignment shown on the right. Nodes with > 95% bootstrap support are annotated with blue circles, the sequence chosen as representative for the OG is highlighted with red text, sequences from *M. capitata* KBHIv3 with blue text, *P. acuta* KBHIv2 with green text, and *S. pistillata* GAJOv1 in purple text. (B) AF2 structure of the OG reference sequence. Relative expression of the one *S. pistillata* gene in the OG in (C) adult, (D) polyp, and (E) larva “cell” types. (F) Differential gene expression results for the one *P. acuta* gene in the OG. Comparisons with significant (FC > 0.5 and adjusted *p*-value < 0.05) differential expression are represented by colored horizontal lines and text describing the FC and adjusted *p*-value. (G) Relative expression of the one *N. vectensis* gene in the OG across each cell type. The > 2.0 FC cutoff used to signify significant cell type specific expression in this study is represented by a horizontal dashed red line in plots C-E and G. (**Page 3**) Phylogeny, structure, and expression of dark genes in the Hexacorallia Dark orthogroup OG000010126. (A) ML phylogeny of the protein sequences in the OG, with associated alignment shown on the right. Nodes with > 95% bootstrap support are annotated with blue circles, the sequence chosen as representative for the OG is highlighted with red text, sequences from *M. capitata* KBHIv3 with blue text, *P. acuta* KBHIv2 with green text, and *S. pistillata* GAJOv1 in purple text. (B) AF2 structure of the OG reference sequence. Relative expression of the one *S. pistillata* gene in the OG in (C) adult, (D) polyp, and (E) larva cell types. The > 2.0 FC cutoff used to signify significant cell type specific expression in this study is represented by a horizontal dashed red line in plots C-E. (F) Differential gene expression results for the one *P. acuta* gene in the OG. Comparisons with significant (FC > 0.5 and adjusted *p*-value < 0.05) differential expression are represented by colored horizontal lines and text describing the FC and adjusted *p*-value.

**Dataset S4. Orthogroup remote homology network.**

Full orthogroup network (a snapshot of which is shown in Figure 4) with nodes colored based on (A) their designation as either dark (unannotated) or light (annotated), (B) the presence of proteins with significant expression across stress and scRNA-seq datasets, (C) the LCA assigned taxonomic level, and (D) the LCA assigned taxa. If an orthogroup contained proteins that showed significant expression across multiple datasets (B), then multiple colors were used for the node. In each image, the layout was calculated using the SFDP algorithm. A legend describing the color used in each image is included as a separate file.

**References**

Chen Y, González-Pech RA, Stephens TG, Bhattacharya D, Chan CX. 2020. Evidence that inconsistent gene prediction can mislead analysis of dinoflagellate genomes. *J. Phycol.* 56:6–10.

He C, Ogata H, Knipp M. 2010. Formation of the complex of nitrite with the ferriheme b beta-barrel proteins nitrophorin 4 and nitrophorin 7. Biochemistry. 49:5841–5851.

Keidel A et al. 2023. Concerted structural rearrangements enable RNA channeling into the cytoplasmic Ski238-Ski7-exosome assembly. Mol. Cell. 83:4093-4105.e7.

Michel G et al. 2004. The structure of chondroitin B lyase complexed with glycosaminoglycan oligosaccharides unravels a calcium-dependent catalytic machinery. J. Biol. Chem. 279:32882–32896.

Waltz F et al. 2021. How to build a ribosome from RNA fragments in Chlamydomonas mitochondria. Nat. Commun. 12:7176.

Weisman CM, Murray AW, Eddy SR. 2022. Mixing genome annotation methods in a comparative analysis inflates the apparent number of lineage-specific genes. *Curr. Biol.* 32:2632-2639.e2.
