# Supplementary material for: Cosmopolitan Gene Families With Known Functions Are Hotspots for the Evolution of Novel Genes in Stony Corals
Source: Genome Biol Evol. 2026 Mar 24;18(4):evag072. doi: 10.1093/gbe/evag072 (PMC13044578; doi:10.1093/gbe/evag072)
Supplement: evag072_Supplementary_Data [file evag072_supplementary_data.zip › Dataset_S4/Dataset_S4/Figure_S4B.RNA_piechart.legend.pdf]

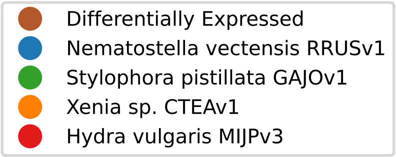
Differentially Expressed
Nematostella vectensis RRUSv1
Stylophora pistillata GAJOv1
Xenia sp. CTEAv1
Hydra vulgaris MIJPv3
