# Supplementary material for: Cosmopolitan Gene Families With Known Functions Are Hotspots for the Evolution of Novel Genes in Stony Corals
Source: Genome Biol Evol. 2026 Mar 24;18(4):evag072. doi: 10.1093/gbe/evag072 (PMC13044578; doi:10.1093/gbe/evag072)
Supplement: evag072_Supplementary_Data [file evag072_supplementary_data.zip › Dataset_S1.pdf]

RNA-seq Datasets

Proportion of dark (purple bars) and light (orange bars) genes with significant (adjusted p-value <0.05 and an absolute log2 fold-change [FC] >0.5) expression at each time point in the Bio-Projects PRJNA694677 (22) (M. capitata 3TP) and PRJNA731596 (23) (M. capitata 12TP and P. acuta 12TP) RNA-seq datasets. For each pair of treatment comparisons (the "Expression Results" panel), the y-axis represents the percentage of significant DEGs of each type (dark or light), out of all significantly DEGs across all comparisons. For the "Total" bars, the y-axis represents the percentage of significant DEGs of each type (dark or light), out of all genes in each species.

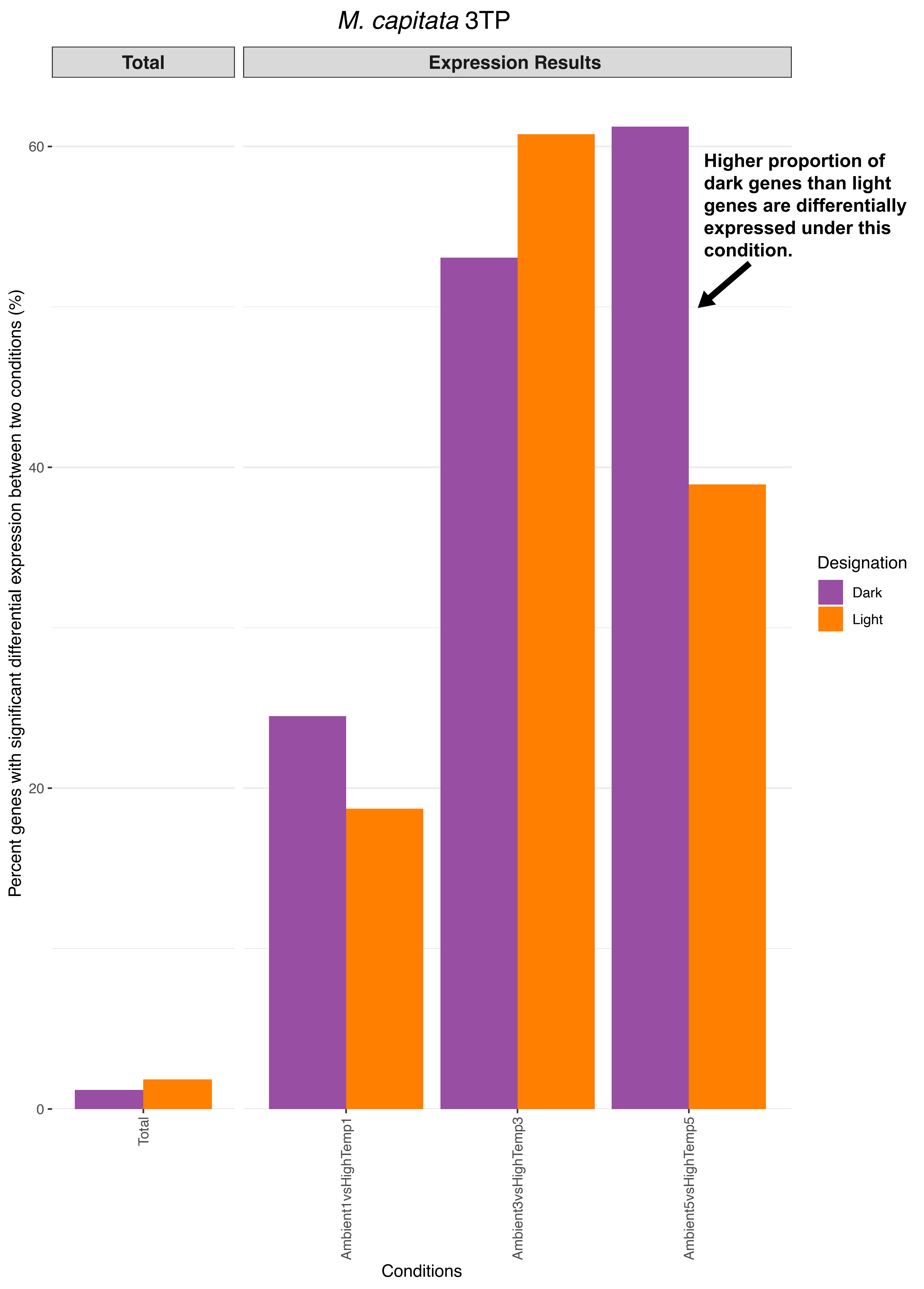

M. capitata 3TP
Total
Expression Results
Higher proportion of dark genes than light genes are differentially expressed under this condition.
Percent genes with significant differential expression between two conditions (%)
60
40
20
0
Total
Ambient1vsHighTemp1
Ambient3vsHighTemp3
Ambient5vsHighTemp5
Conditions
Designation
Dark
Light

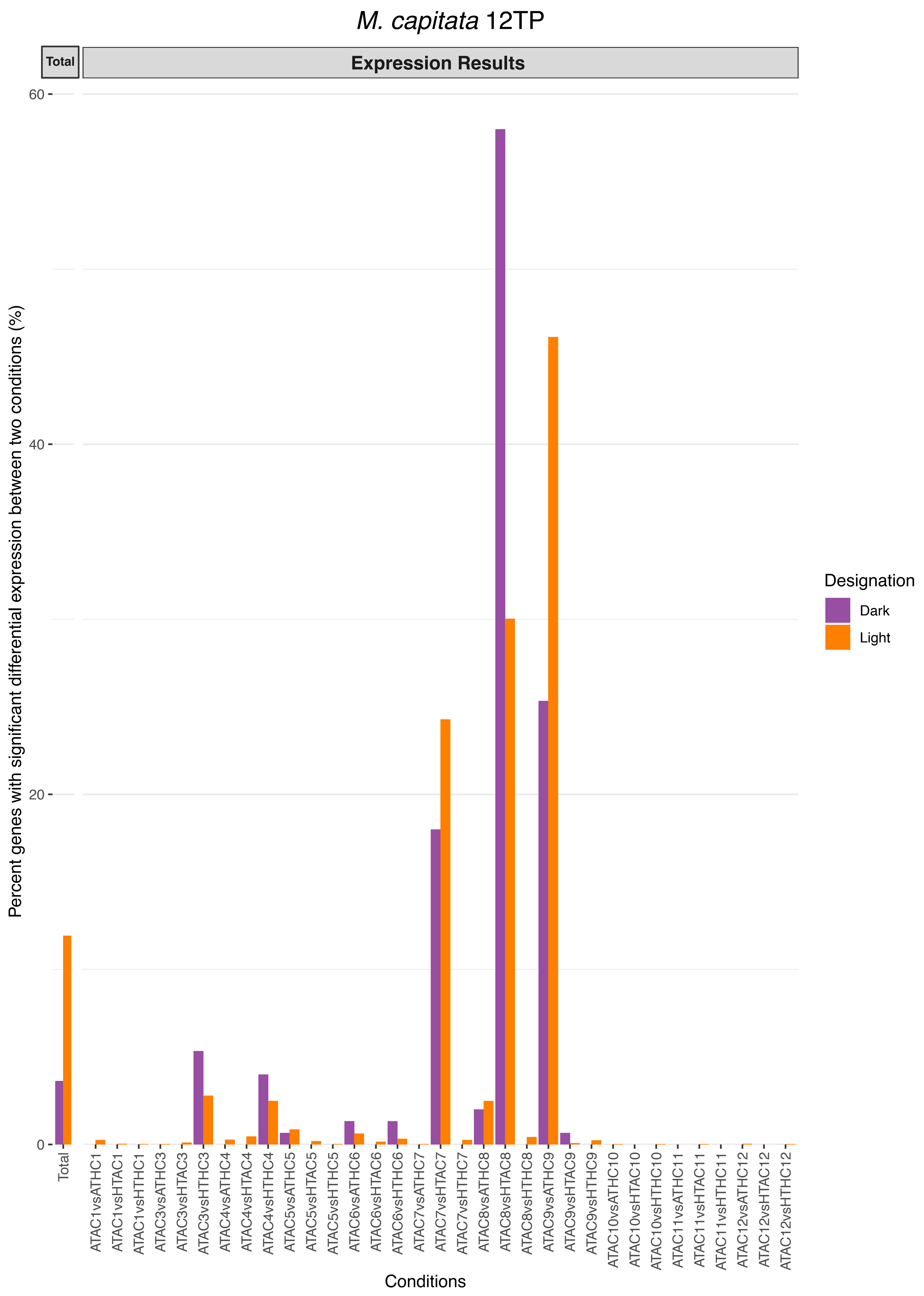
M. capitata 12TP
Total
Expression Results
60
40
20
0
Percent genes with significant differential expression between two conditions (%)
Total
ATAC1vsATHC1
ATAC1vsHTAC1
ATAC1vsHTHC1
ATAC3vsATHC3
ATAC3vsHTAC3
ATAC3vsHTHC3
ATAC4vsATHC4
ATAC4vsHTAC4
ATAC4vsHTHC4
ATAC5vsATHC5
ATAC5vsHTAC5
ATAC5vsHTHC5
ATAC6vsATHC6
ATAC6vsHTAC6
ATAC6vsHTHC6
ATAC7vsATHC7
ATAC7vsHTAC7
ATAC7vsHTHC7
ATAC8vsATHC8
ATAC8vsHTAC8
ATAC8vsHTHC8
ATAC9vsATHC9
ATAC9vsHTAC9
ATAC9vsHTHC9
ATAC10vsATHC10
ATAC10vsHTAC10
ATAC10vsHTHC10
ATAC11vsATHC11
ATAC11vsHTAC11
ATAC11vsHTHC11
ATAC12vsATHC12
ATAC12vsHTAC12
ATAC12vsHTHC12
Conditions
Designation
Dark
Light

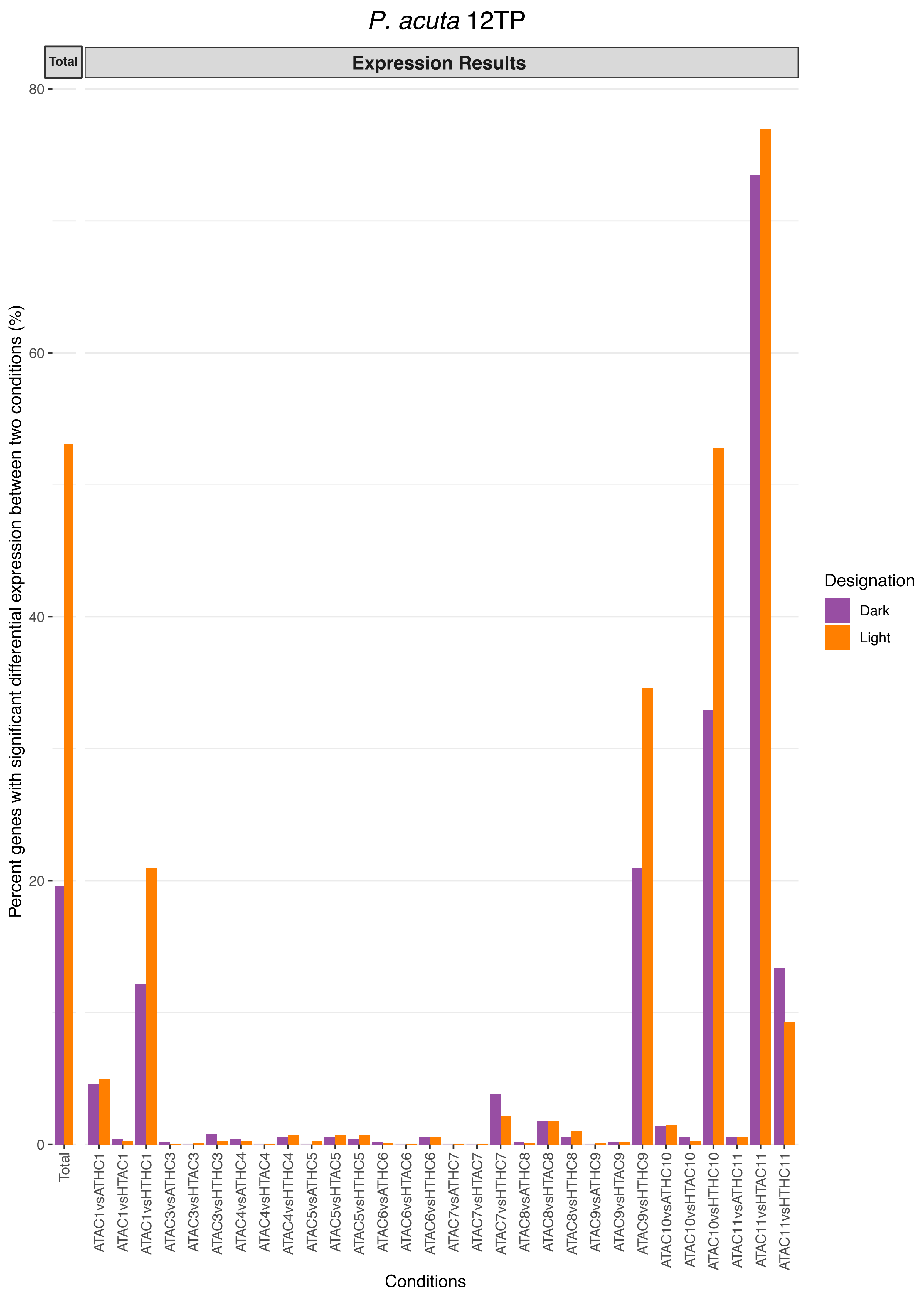
P. acuta 12TP
Total
Expression Results
Percent genes with significant differential expression between two conditions (%)
80
60
40
20
0
Total
ATAC1vsATHC1
ATAC1vsHTAC1
ATAC1vsHTHC1
ATAC3vsATHC3
ATAC3vsHTAC3
ATAC3vsHTHC3
ATAC4vsATHC4
ATAC4vsHTAC4
ATAC4vsHTHC4
ATAC5vsATHC5
ATAC5vsHTAC5
ATAC5vsHTHC5
ATAC6vsATHC6
ATAC6vsHTAC6
ATAC6vsHTHC6
ATAC7vsATHC7
ATAC7vsHTAC7
ATAC7vsHTHC7
ATAC8vsATHC8
ATAC8vsHTAC8
ATAC8vsHTHC8
ATAC9vsATHC9
ATAC9vsHTAC9
ATAC9vsHTHC9
ATAC10vsATHC10
ATAC10vsHTAC10
ATAC10vsHTHC10
ATAC11vsATHC11
ATAC11vsHTAC11
ATAC11vsHTHC11
Conditions
Designation
Dark
Light

scRNA-seq Datasets

Proportion of dark (purple bars) and light (orange bars) genes with significant (fold-change [FC] > 2) expression in each "metacell", "cell", and "broadcell" type in the Stylophora pistillata (4), Xenia sp. (24), Nematostella vectensis (25), and Hydra vulgaris (26) single-cell RNA-seq datasets scRNA-seq datasets. Bars are not stacked but overlayed on top of each other, with the smaller bar being placed on top of the larger for clarity. Bars at the "metacell" and "cell" levels are grouped under their "broadcell" types in each figure; the "broadcell" types used to group each set of bars are shown in colored boxes at the top of each figure. For each cell type, the y-axis represents the percentage of significantly expressed genes of each type (dark or light), out of all significantly expressed genes across all cell types. For the "Total" bars, the y-axis represents the percentage of significantly expressed genes of each type (dark or light), out of all genes in each species.

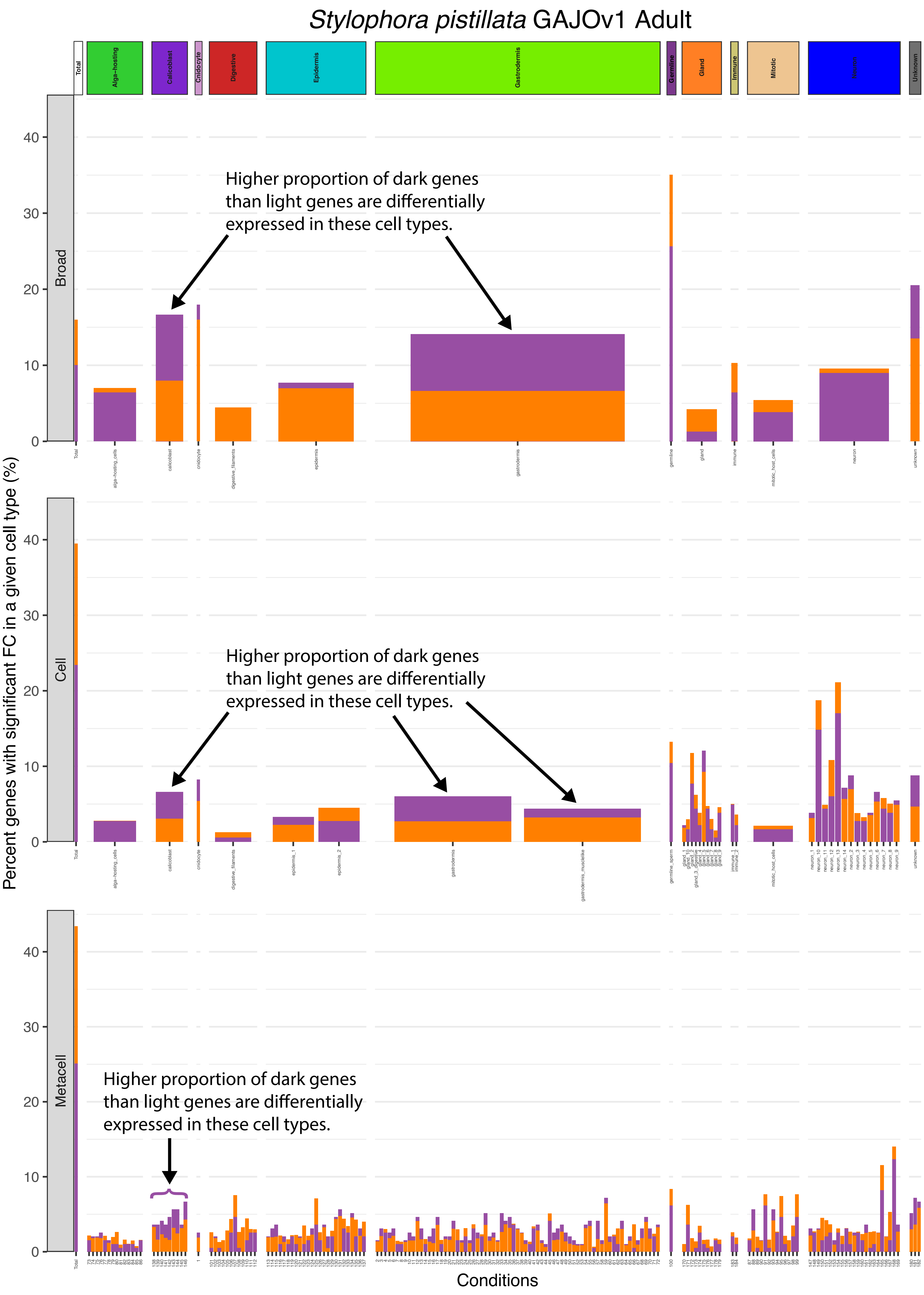
Stylophora pistillata GAJOv1 Adult
Total
Alga-hosting
Calicoblast
Cnidocyte
Digestive
Epidermis
Gastrodermis
Germline
Gland
Immune
Mitotic
Neuron
Unknown
Broad
Cell
Metacell
Higher proportion of dark genes than light genes are differentially expressed in these cell types.
Higher proportion of dark genes than light genes are differentially expressed in these cell types.
Higher proportion of dark genes than light genes are differentially expressed in these cell types.
Percent genes with significant FC in a given cell type (%)
Conditions

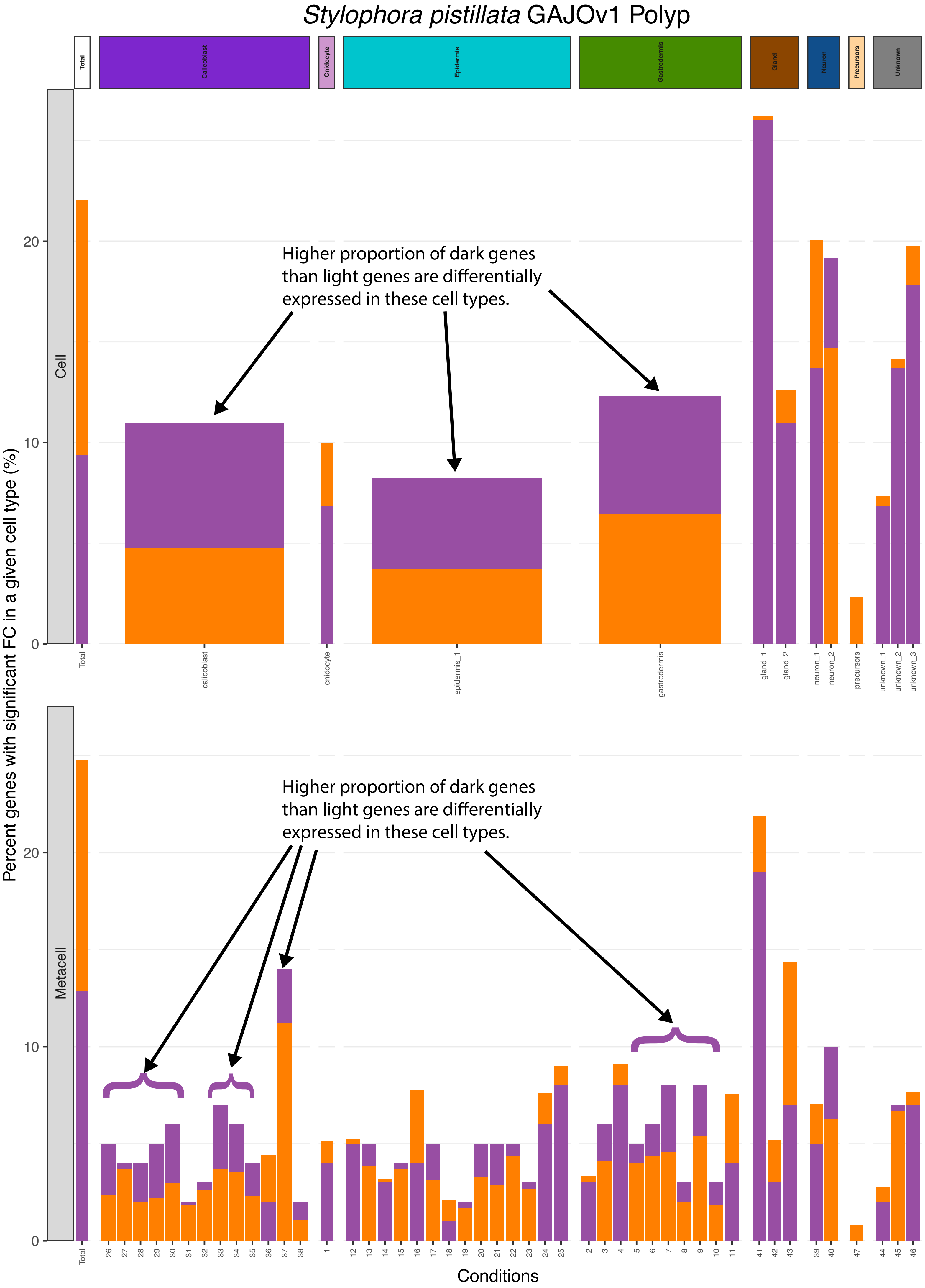

Stylophora pistillata GAJOv1 Polyp
Total
Calicoblast
Cnidocyte
Epidermis
Gastrodermis
Gland
Neuron
Precursors
Unknown
Cell
Metacell
Higher proportion of dark genes than light genes are differentially expressed in these cell types.
Higher proportion of dark genes than light genes are differentially expressed in these cell types.
Percent genes with significant FC in a given cell type (%)
Conditions
0
10
20
Total
calicoblast
cnidocyte
epidermis_1
gastrodermis
gland_1
gland_2
neuron_1
neuron_2
precursors
unknown_1
unknown_2
unknown_3

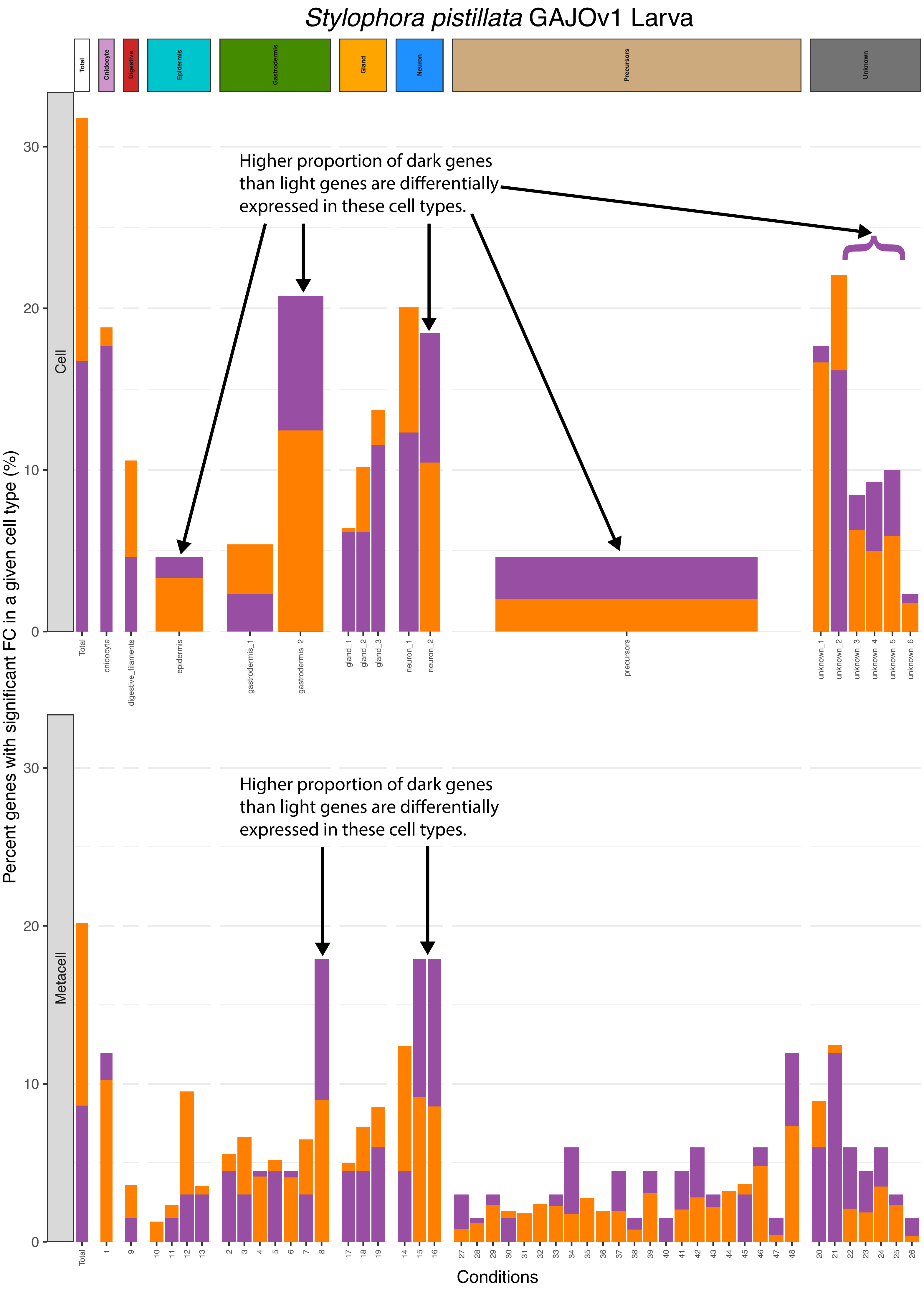

Stylophora pistillata GAJOv1 Larva
Total
Cnidocyte
Digestive
Epidermis
Gastrodermis
Gland
Neuron
Precursors
Unknown
Cell
Metacell
Higher proportion of dark genes than light genes are differentially expressed in these cell types.
Higher proportion of dark genes than light genes are differentially expressed in these cell types.
Percent genes with significant FC in a given cell type (%)
Conditions
cnidocyte
digestive_filaments
epidermis
gastrodermis_1
gastrodermis_2
gland_1
gland_2
gland_3
neuron_1
neuron_2
precursors
unknown_1
unknown_2
unknown_3
unknown_4
unknown_5
unknown_6

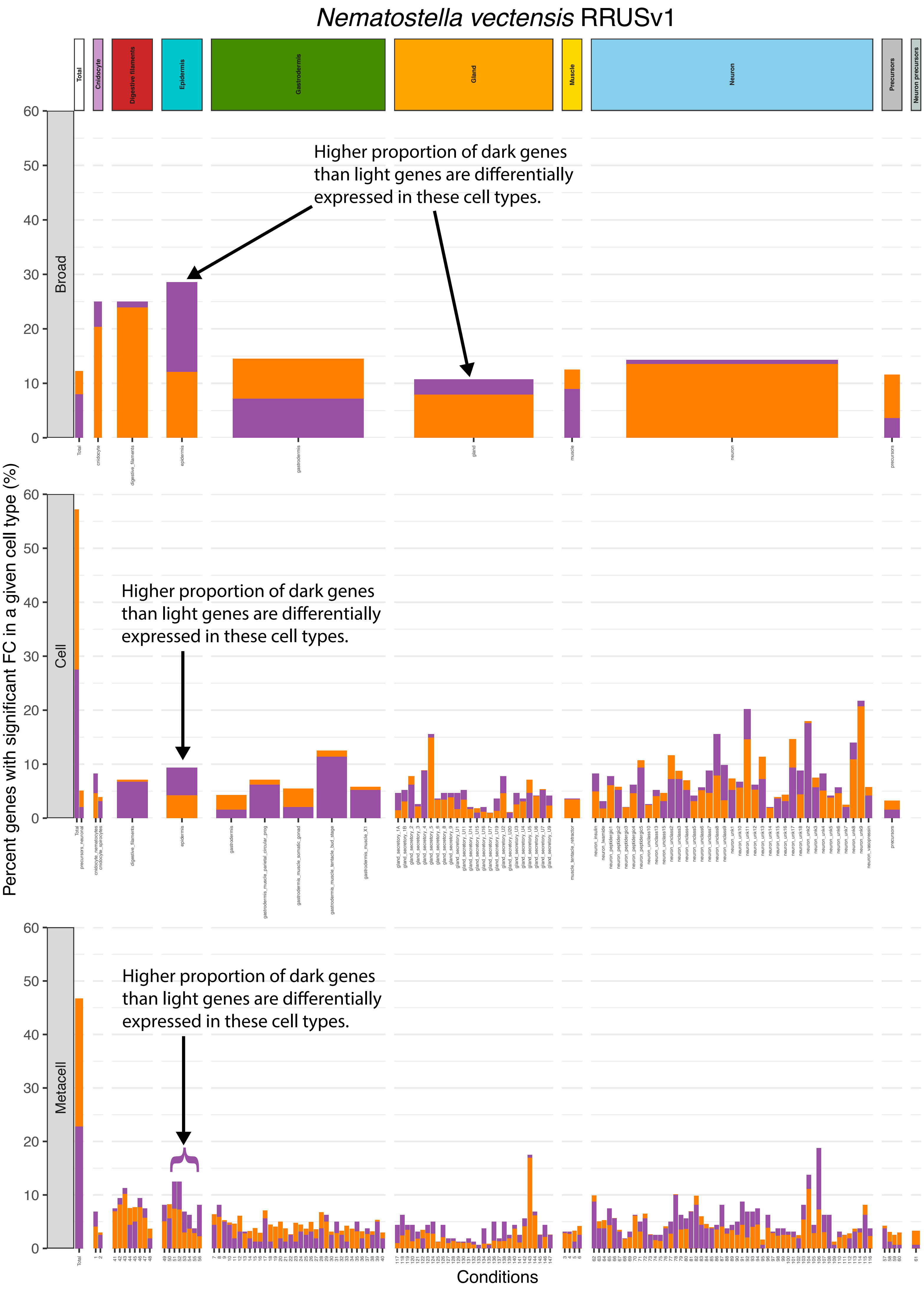

Nematostella vectensis RRUSv1
Total
Cnidocyte
Digestive filaments
Epidermis
Gastrodermis
Gland
Muscle
Neuron
Precursors
Neuron precursors
Broad
Cell
Metacell
Higher proportion of dark genes than light genes are differentially expressed in these cell types.
Higher proportion of dark genes than light genes are differentially expressed in these cell types.
Higher proportion of dark genes than light genes are differentially expressed in these cell types.
Percent genes with significant FC in a given cell type (%)
Conditions

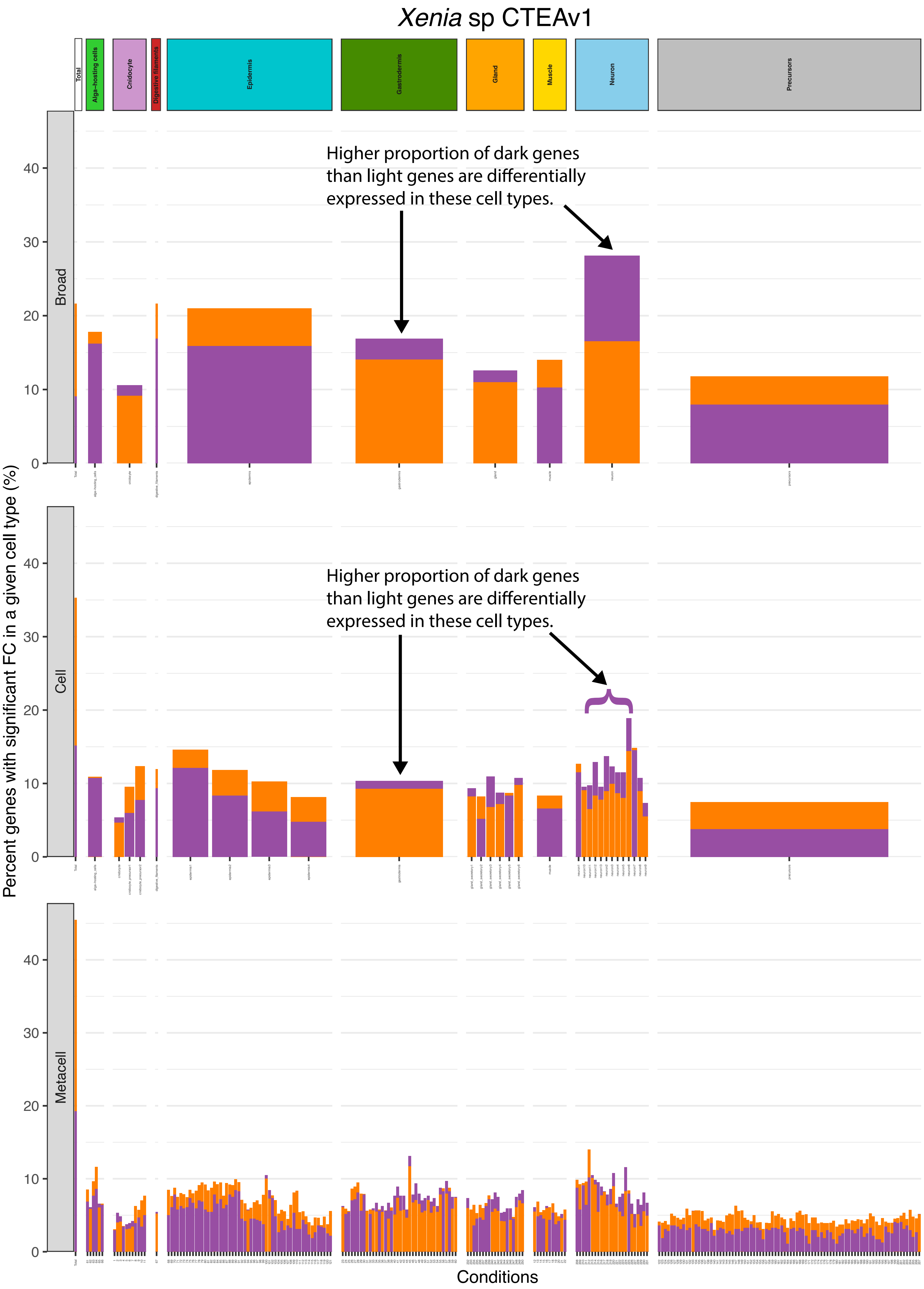

Xenia sp CTEAv1
Total
Alga-hosting cells
Cnidocyte
Digestive filaments
Epidermis
Gastrodermis
Gland
Muscle
Neuron
Precursors
Broad
Cell
Metacell
Higher proportion of dark genes than light genes are differentially expressed in these cell types.
Higher proportion of dark genes than light genes are differentially expressed in these cell types.
Percent genes with significant FC in a given cell type (%)
Conditions
0
10
20
30
40

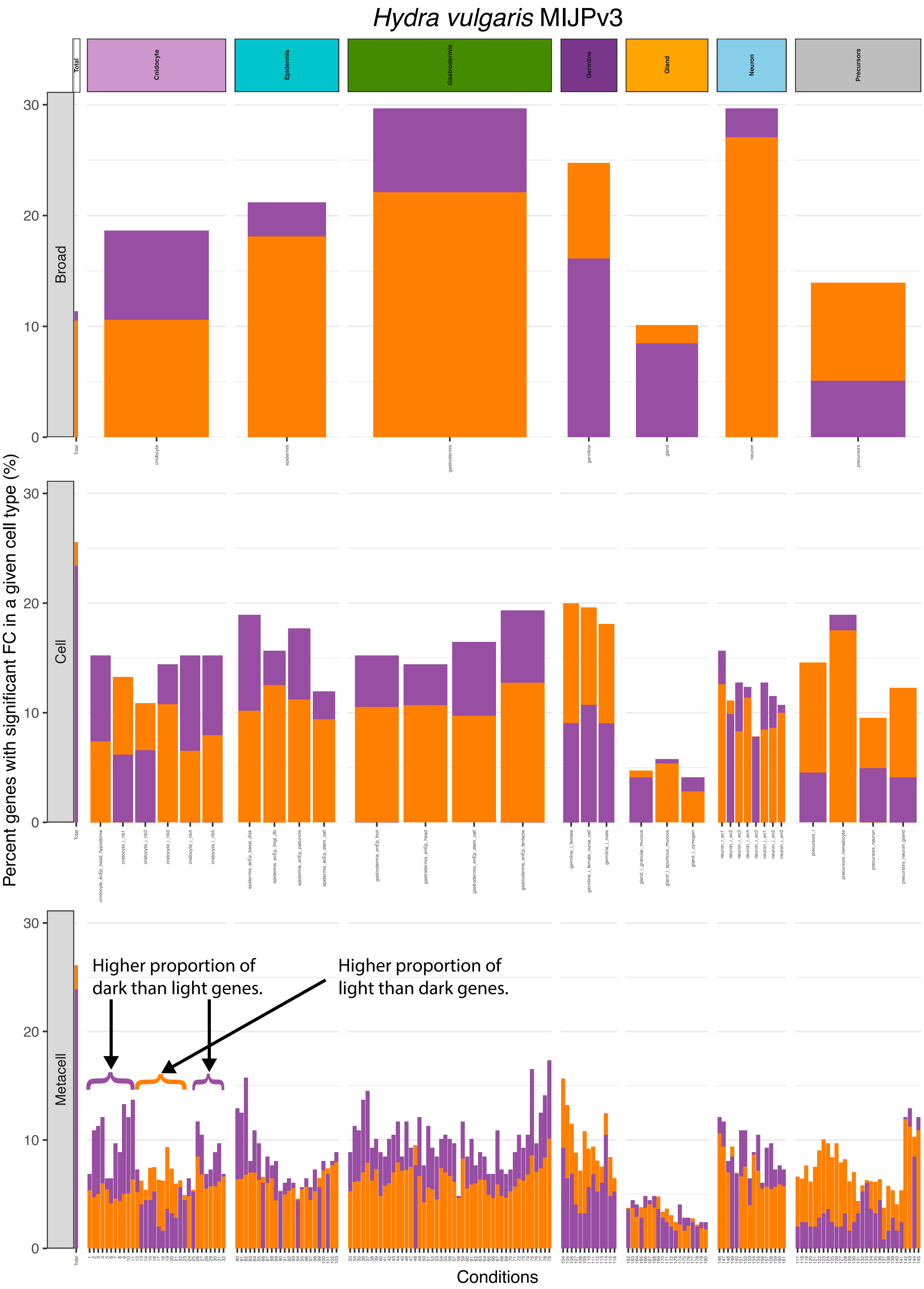

Hydra vulgaris MIJPv3
Total
Cnidocyte
Epidermis
Gastrodermis
Germline
Gland
Neuron
Precursors
Broad
Cell
Metacell
Percent genes with significant FC in a given cell type (%)
0
10
20
30
Higher proportion of dark than light genes.
Higher proportion of light than dark genes.
Conditions
