# Supplementary material for: Cosmopolitan Gene Families With Known Functions Are Hotspots for the Evolution of Novel Genes in Stony Corals
Source: Genome Biol Evol. 2026 Mar 24;18(4):evag072. doi: 10.1093/gbe/evag072 (PMC13044578; doi:10.1093/gbe/evag072)

Figure 1

Ependymin-related blue carotenoprotein (EPD-BCP), (AF2 ID: OG000001644).

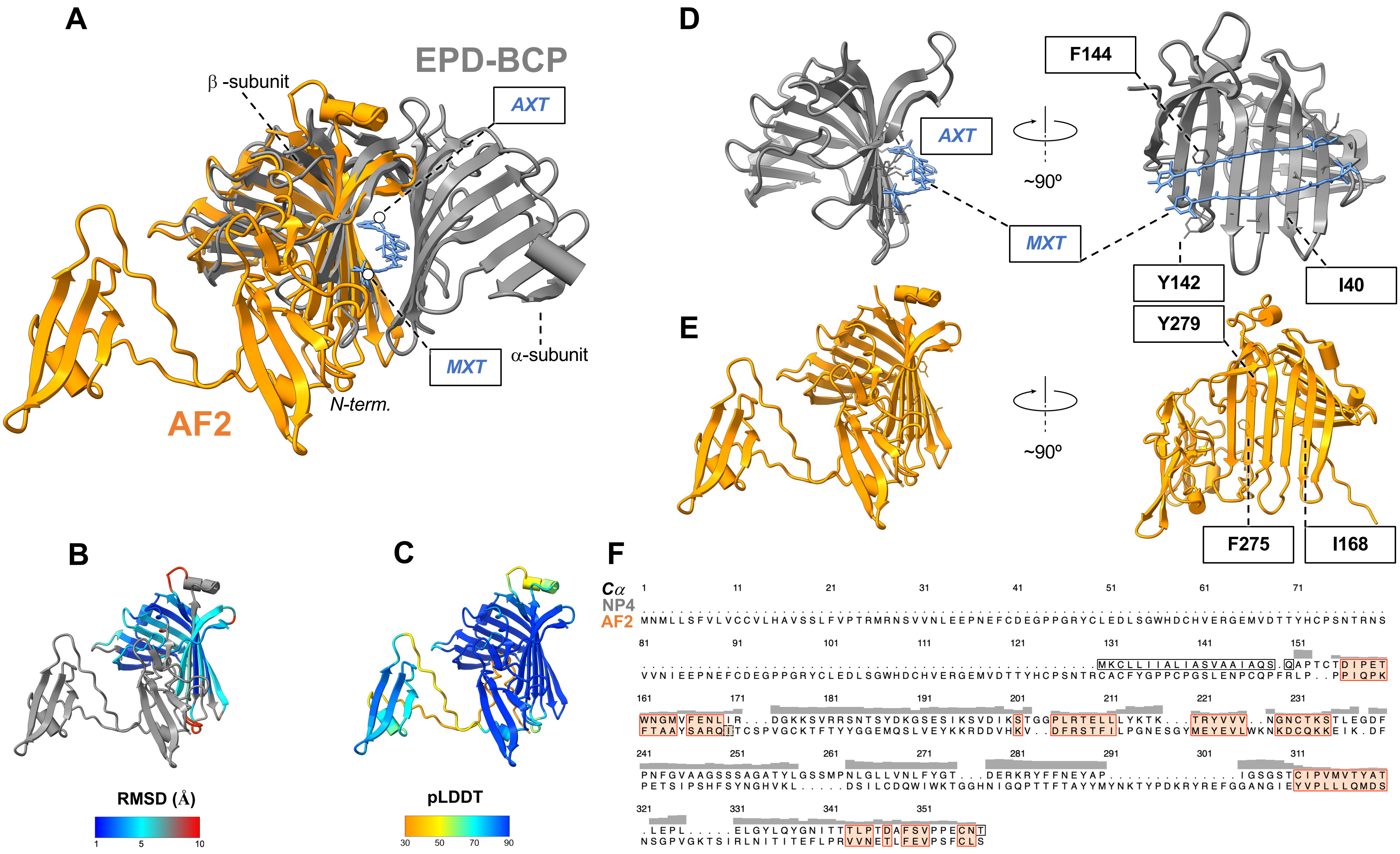

Figure 2

Nitrophorin4-like (Np4) protein (AF2 ID: OG000010602).

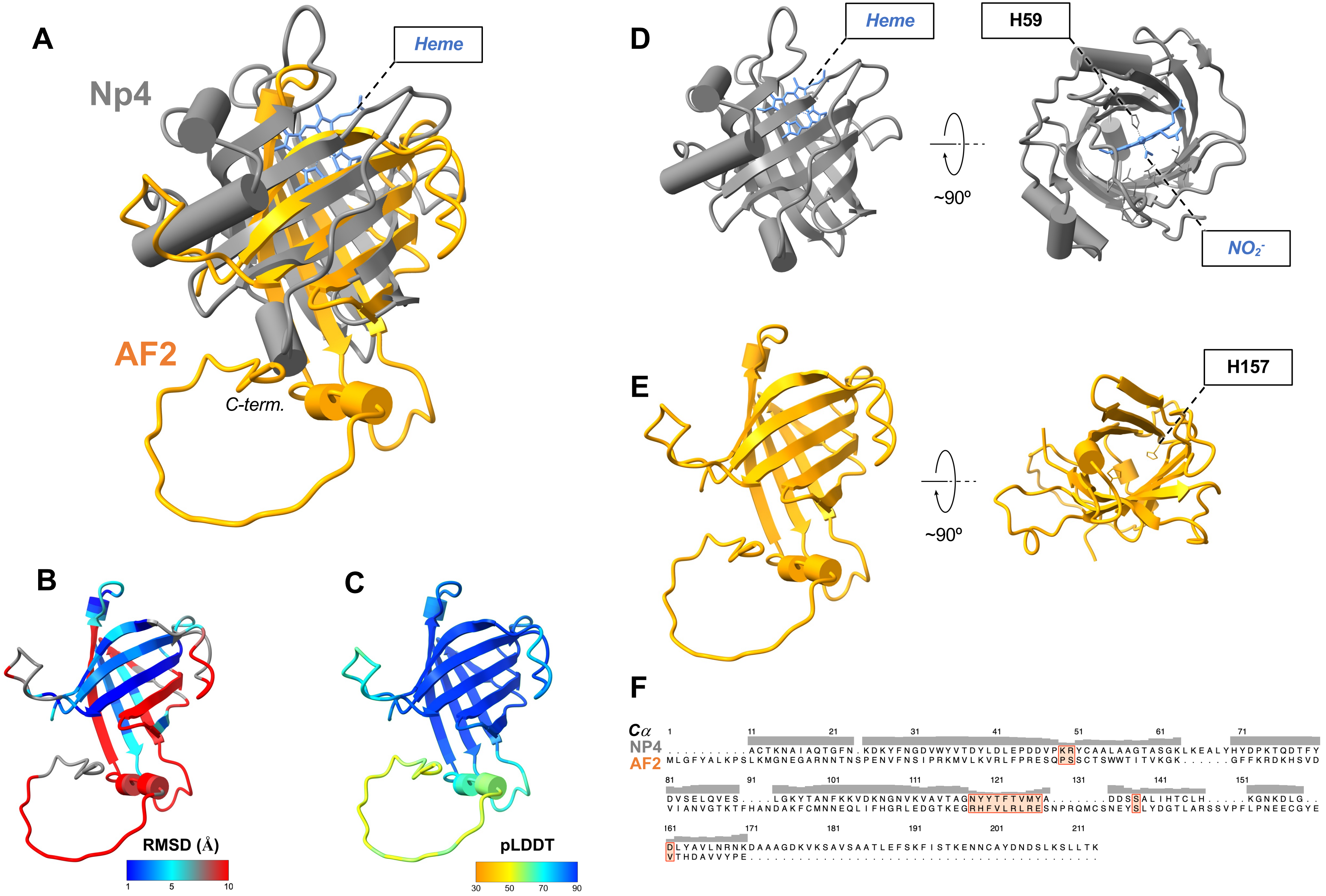

Figure 3

Chondroitin B (ChonB) lyase-like protein (AF2 ID: OG000009717).

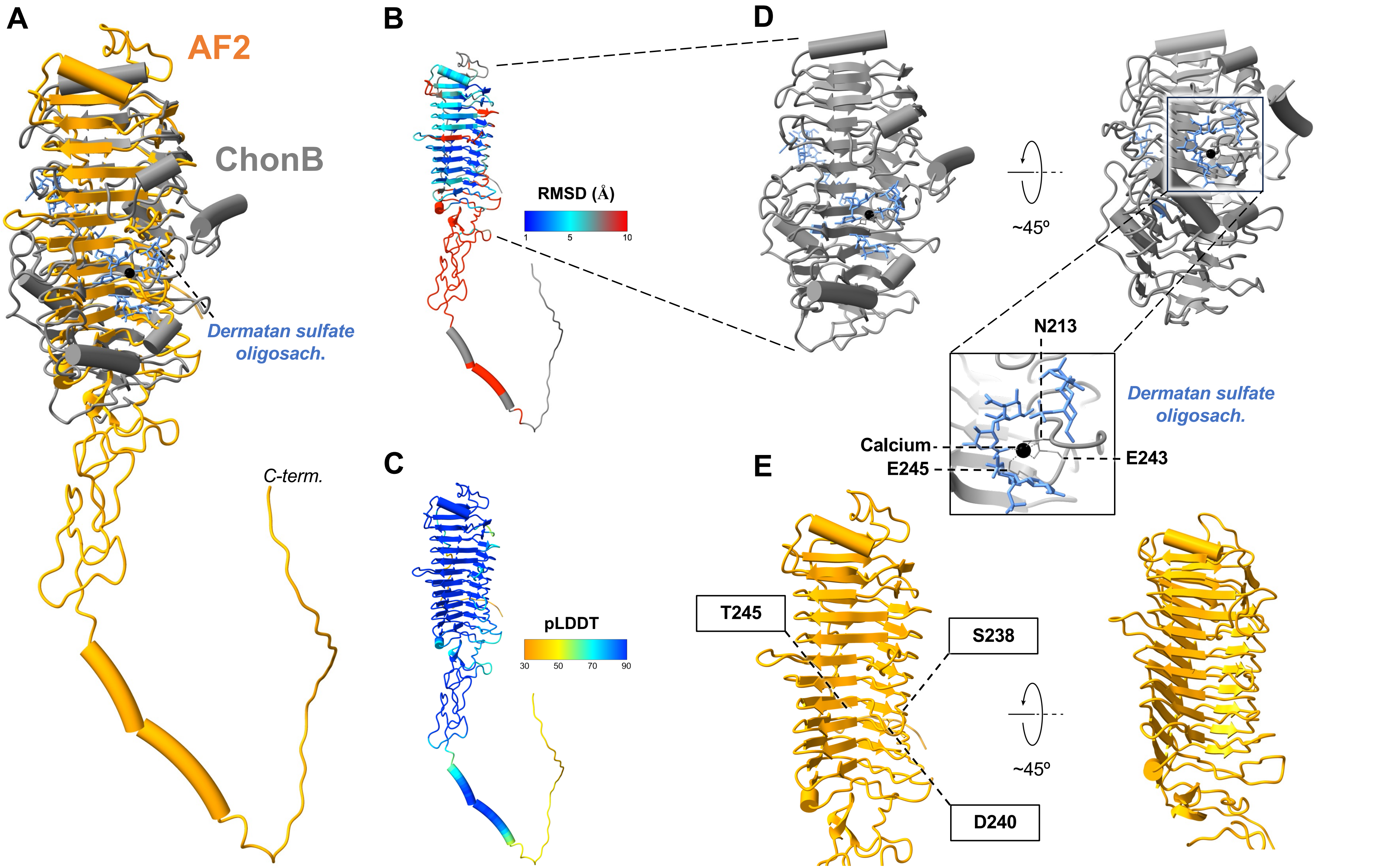

F

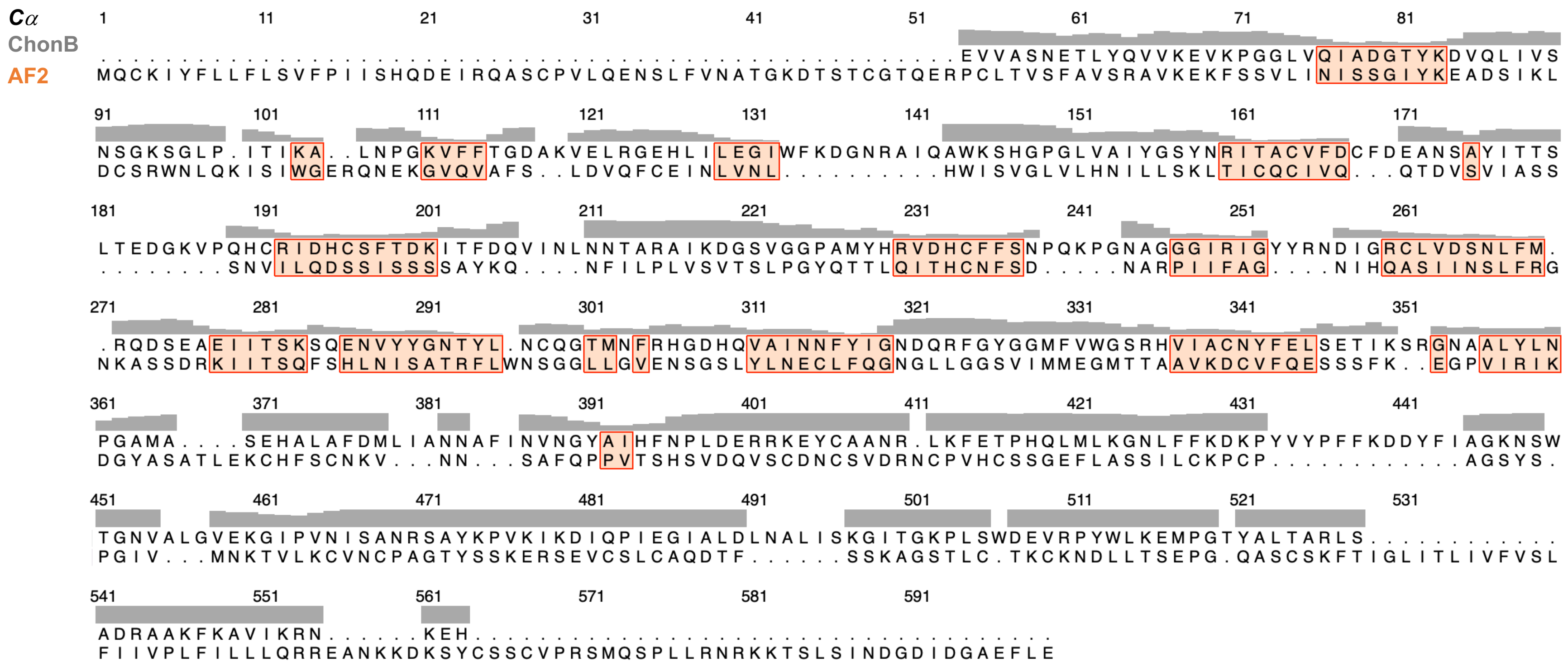

Cα
ChonB
AF2
1 11 21 31 41 51 61 71 81
....EVVASNETLYQVVKEVKPGGLVQIADGTYKDVQLIVS
MQCKIYFLLFLSVFPIISHQDEIRQASCPVLQENSLFVNATGKDTSTCGTQERPCLTVSFAVSRAVKEKFSSVLINISSGIYKEADSIKL
91 101 111 121 131 141 151 161 171
NSGKSGLP.ITIKA..LNPGKVFFTGDAKVELRGEHLILEGIWFKDGNRAIQAWKSHGPGLVAIYGSYNRITACVFDCFDEANSAYITTS
DCSRWNLQKISIWGERQNEKGVQVAFS..LDVQFCEINLVNL..........HWISVGLVLHNILLSKLTICQCIVQ...QTDVSVIASS
181 191 201 211 221 231 241 251 261
LTEDGKVPQHCRIDHCSFTDKITFDQVINLNNTARAIKDGSVGGPAMYHRVDHCFFSNPQKPGNAGGGIRIGYYRNDIGRCLVDSNLFM.
........SNVILQDSSISSSSAYKQ....NFILPLVSVTSLPGYQTTLQITHCNFSD.....NARPIIFAG....NIHQASIINSLFRG
271 281 291 301 311 321 331 341 351
.RQDSEAEIITSKSQENVYYGNTYL.NCQGTMNFRHGDHQVAINNFYIGNDQRFGYGGMFVWGSRHVIACNYFELSETIKSRGNAALYLN
NKASSDRKIITSQFSHLNISATRFLWNSGGLLGVENSGSLYLNECLFQGNGLLGGSVIMMEGMTTAAVKDCVFQESSSFK..EGPVIRIK
361 371 381 391 401 411 421 431 441
PGAMA....SEHALAFDMLIANNAFINVNGYAIHFNPLDERRKEYCAANR.LKFETPHQLMLKGNLFFKDKPYVYPFFKDDYFIAGKNSW
DGYASATLEKCHFSCNKV...NN...SAFQPPVTSHSVDQVSCDNCSVDRNCPVHCSSGEFLASSILCKPCP...........AGSYS.
451 461 471 481 491 501 511 521 531
TGNVALGVEKGIPVNISANRSAYKPVKIKDIQPIEGIALDLNALISKGITGKPLSWDEVRPYWLKEMPGTYALTARLS............
PGIV...MNKTVLKCVNCPAGTYSSKERSEVCSLCAQDTF......SSKAGSTLC.TKCKNDLLTSEPG.QASCSKFTIGLITLIVFVSL
541 551 561 571 581 591
ADRAAKFKAVIKRN......KEH....................................
FIIVPLFILLLQRREANKKDKSYCSSCVPRSMQSPLLRNRKKTSLSINDGDIDGAEFLE

Figure 4

Superkiller 8 (Ski8)-like protein (AF2 ID: OG000008812).

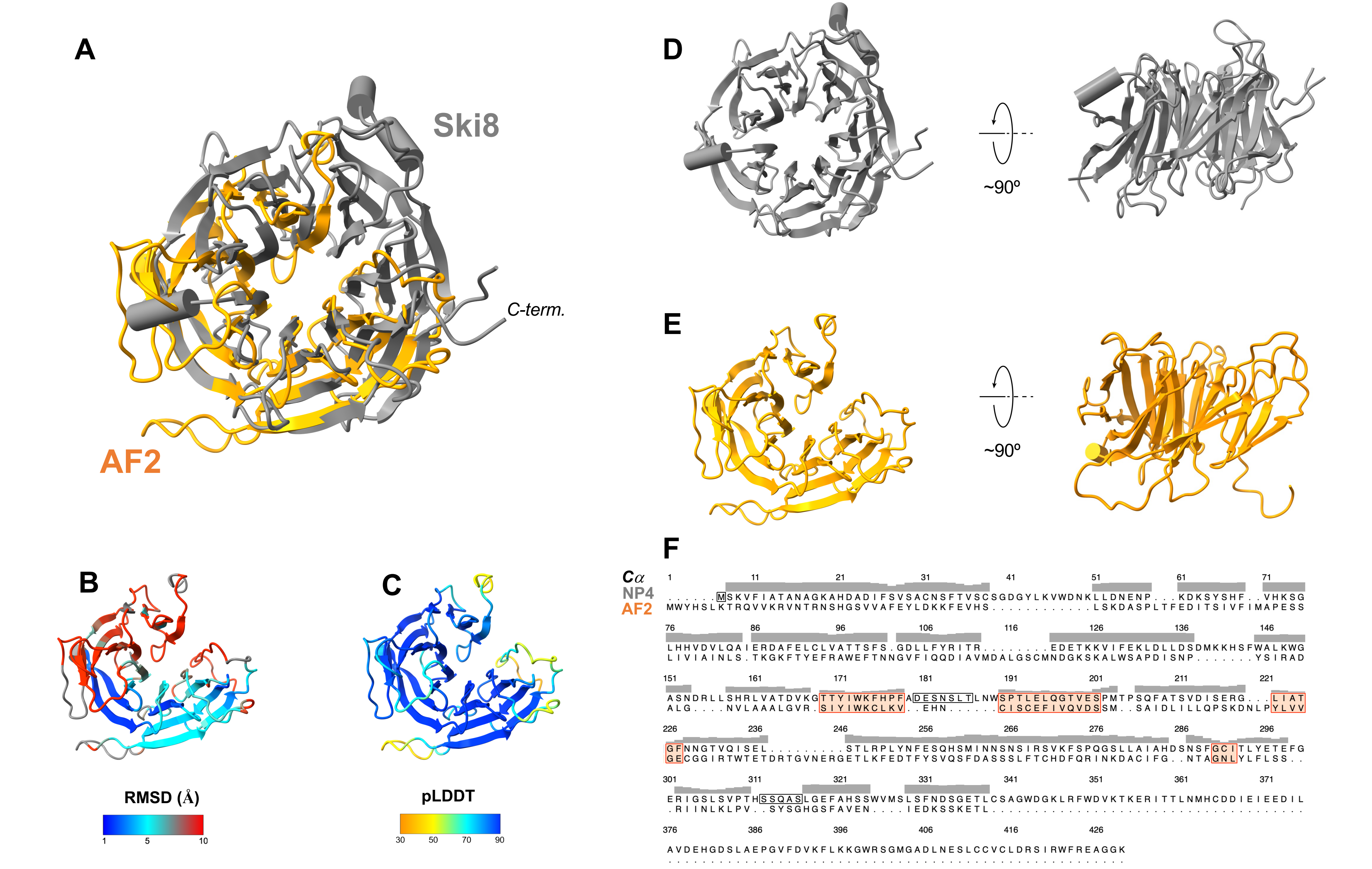

Figure 5

Mitochondrial ribosomal-like protein (ml115), (AF2 ID: OG000011843).

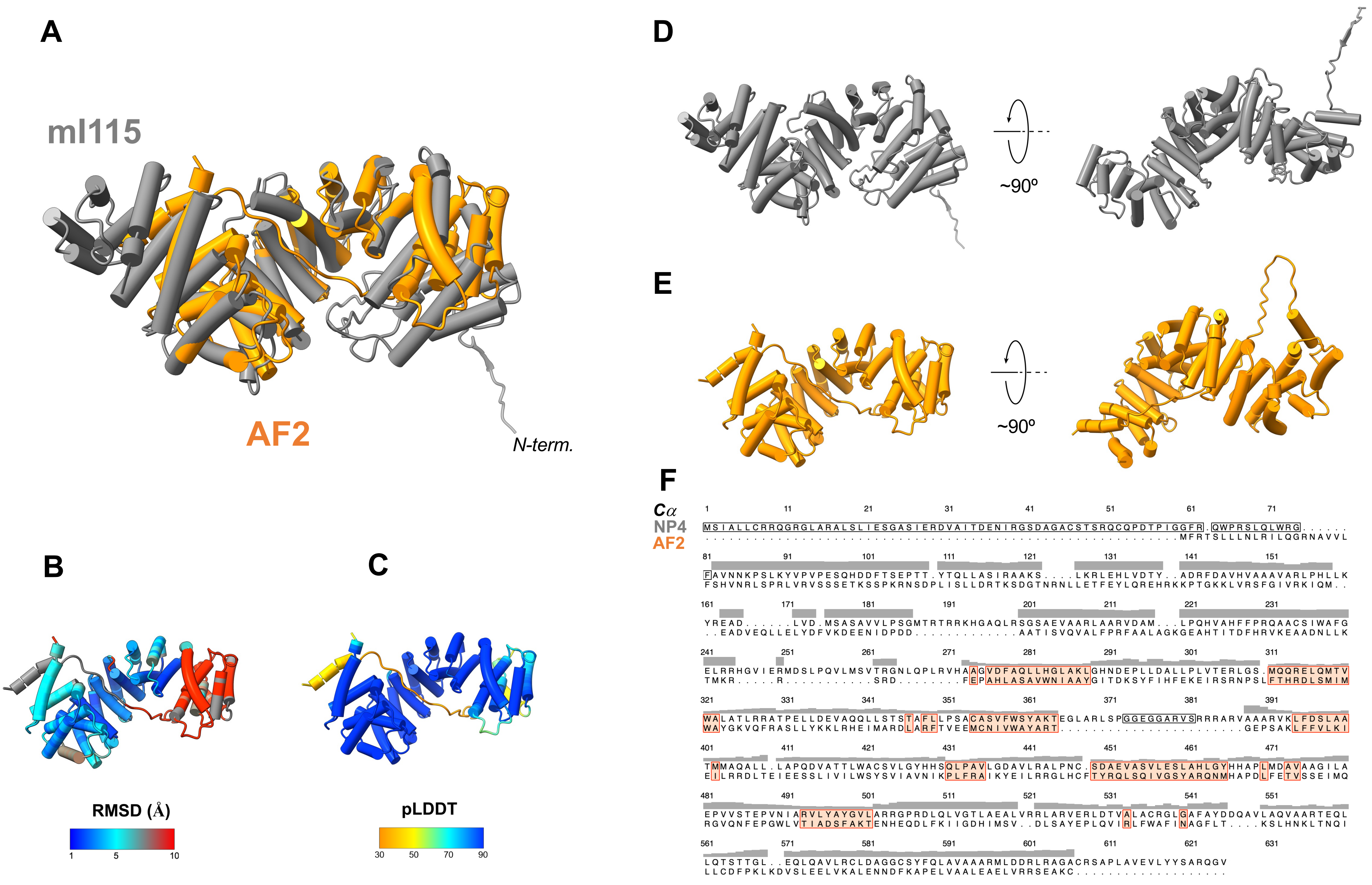

Supplement: evag072_Supplementary_Data [file evag072_supplementary_data.zip › Dataset_S2.pdf]
