# Supplementary figures and images for: Cosmopolitan Gene Families With Known Functions Are Hotspots for the Evolution of Novel Genes in Stony Corals
Source: Genome Biol Evol. 2026 Mar 24;18(4):evag072. doi: 10.1093/gbe/evag072 (PMC13044578; doi:10.1093/gbe/evag072)

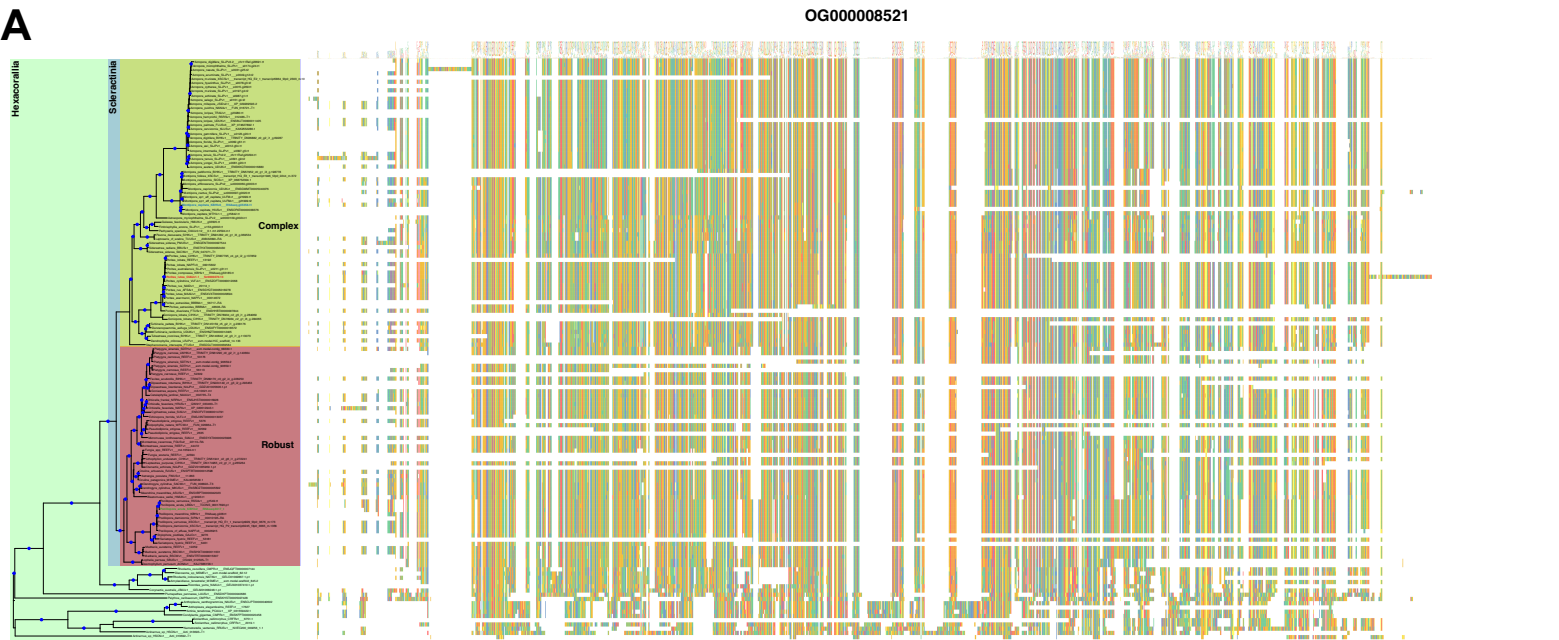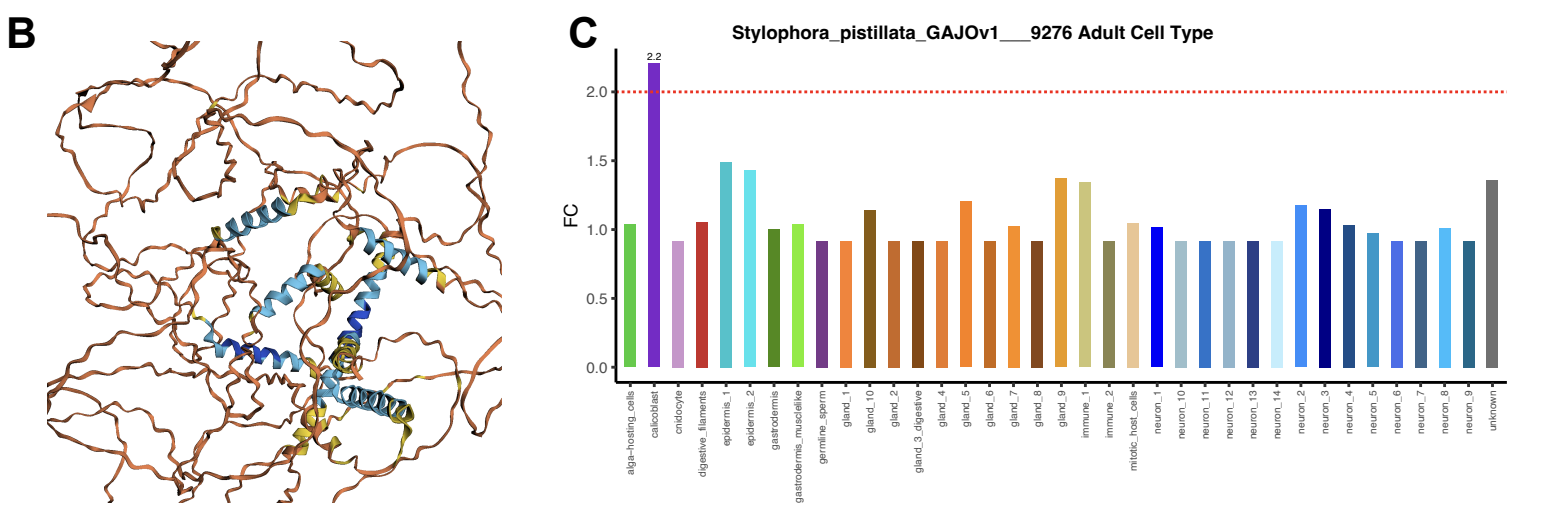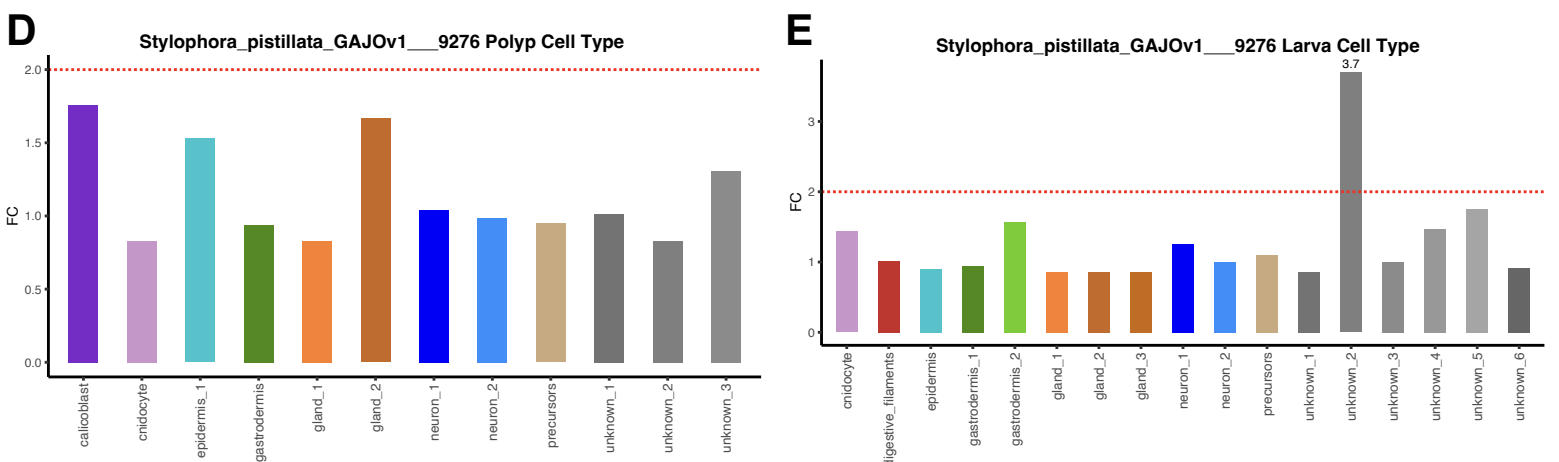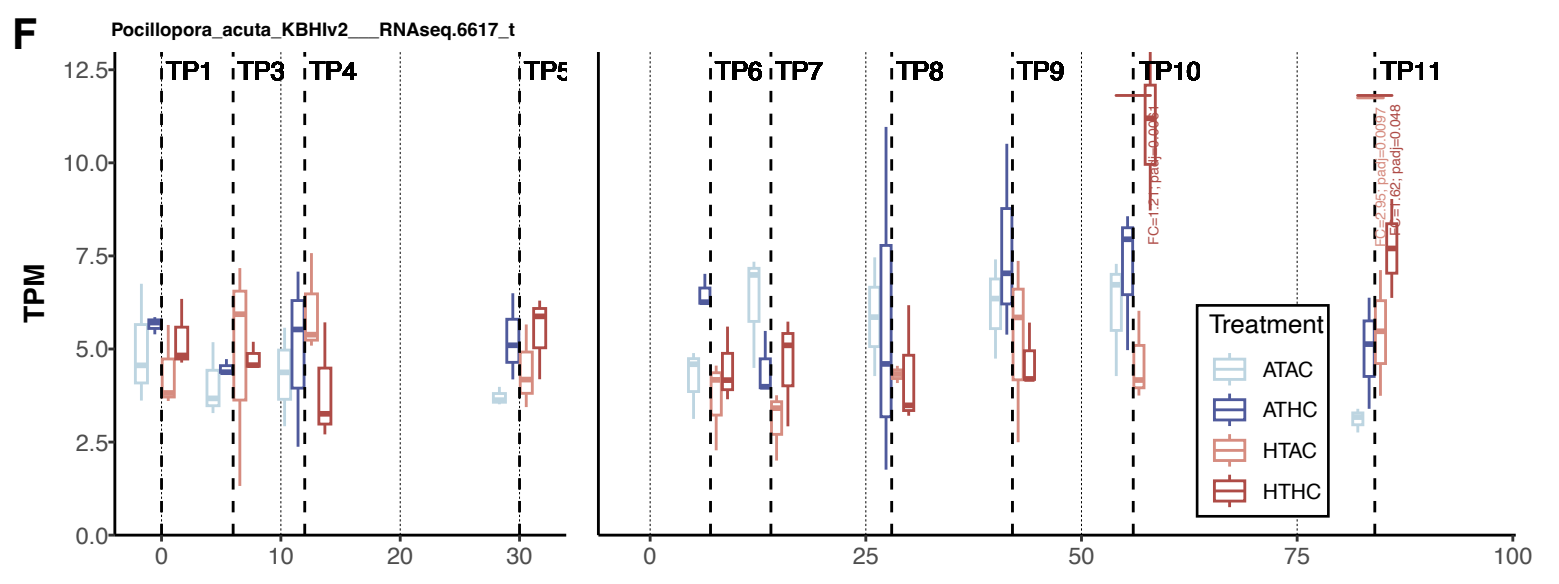

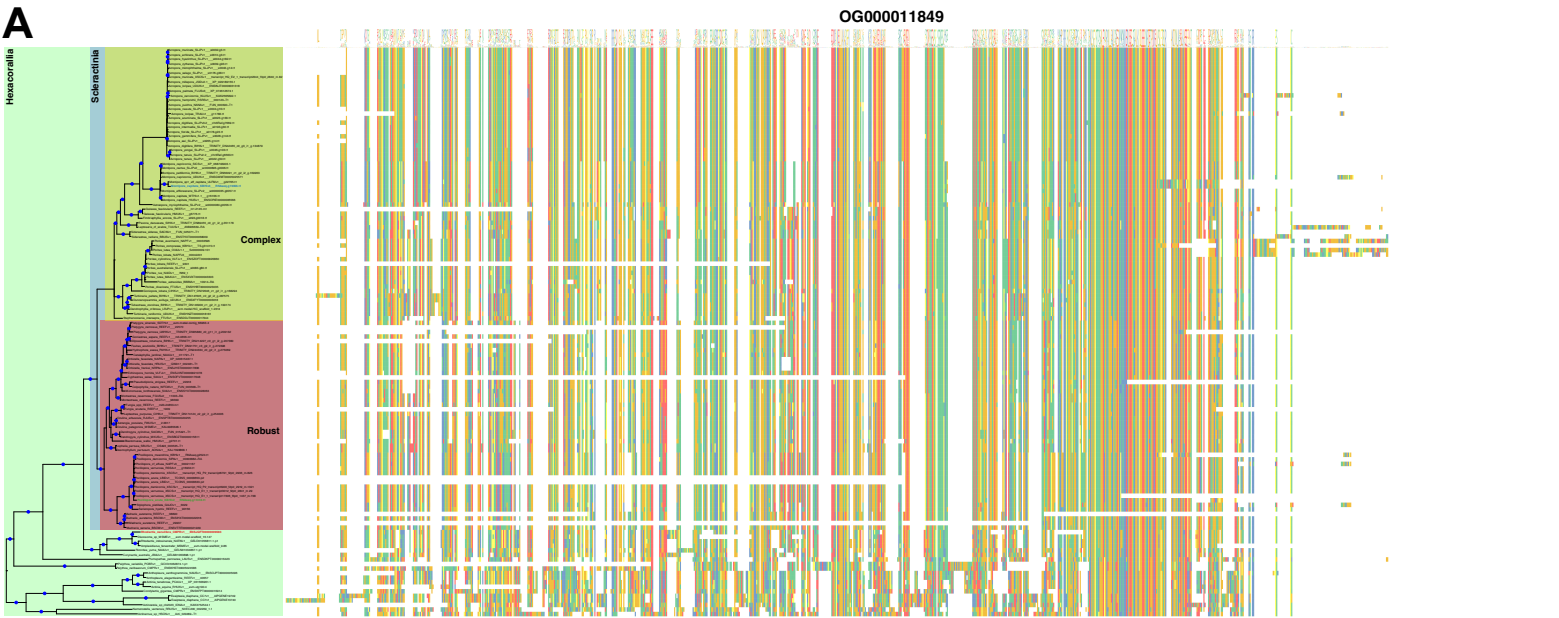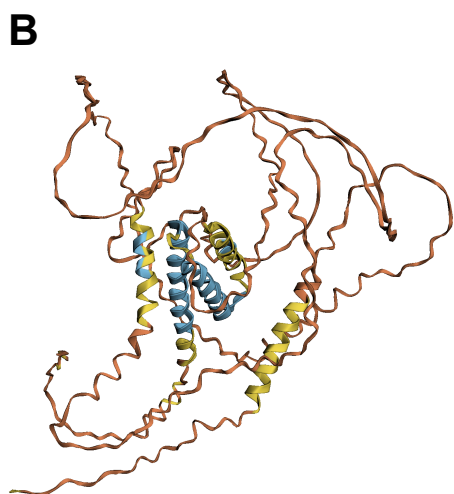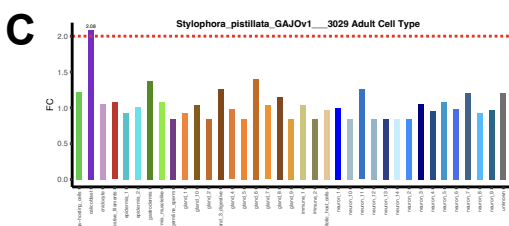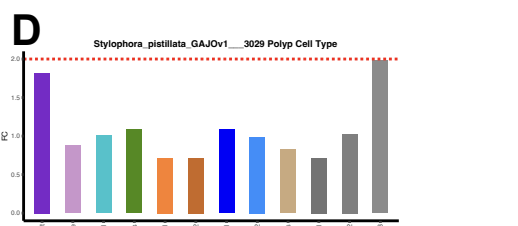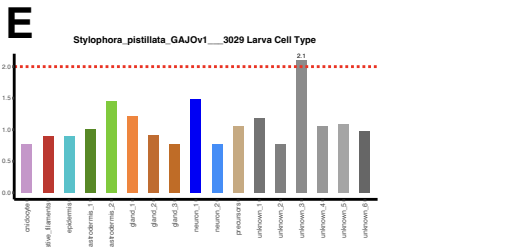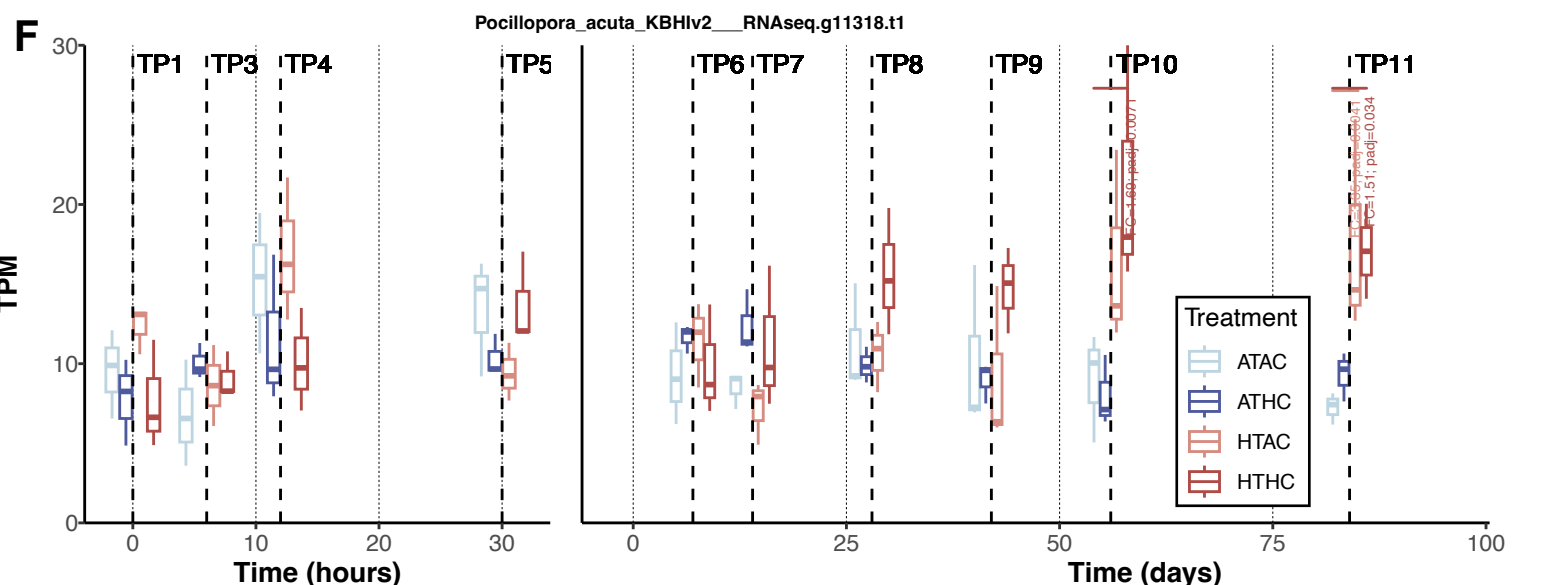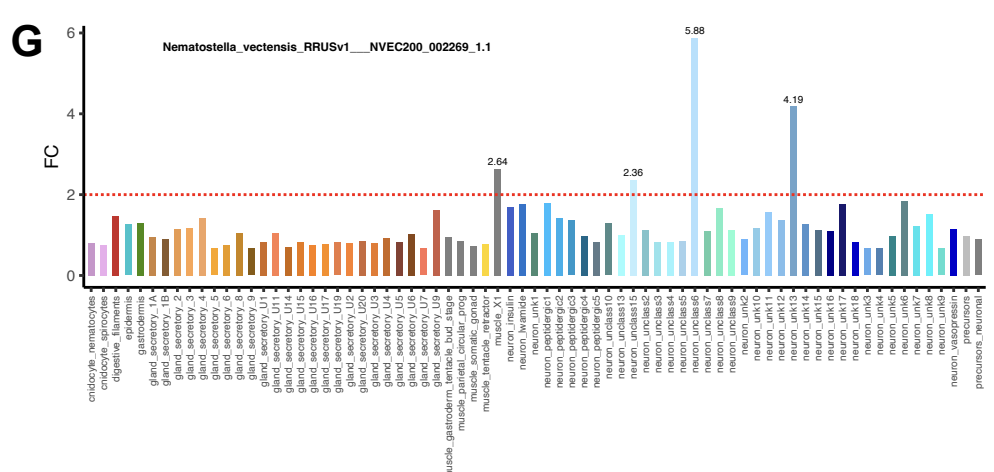

**A**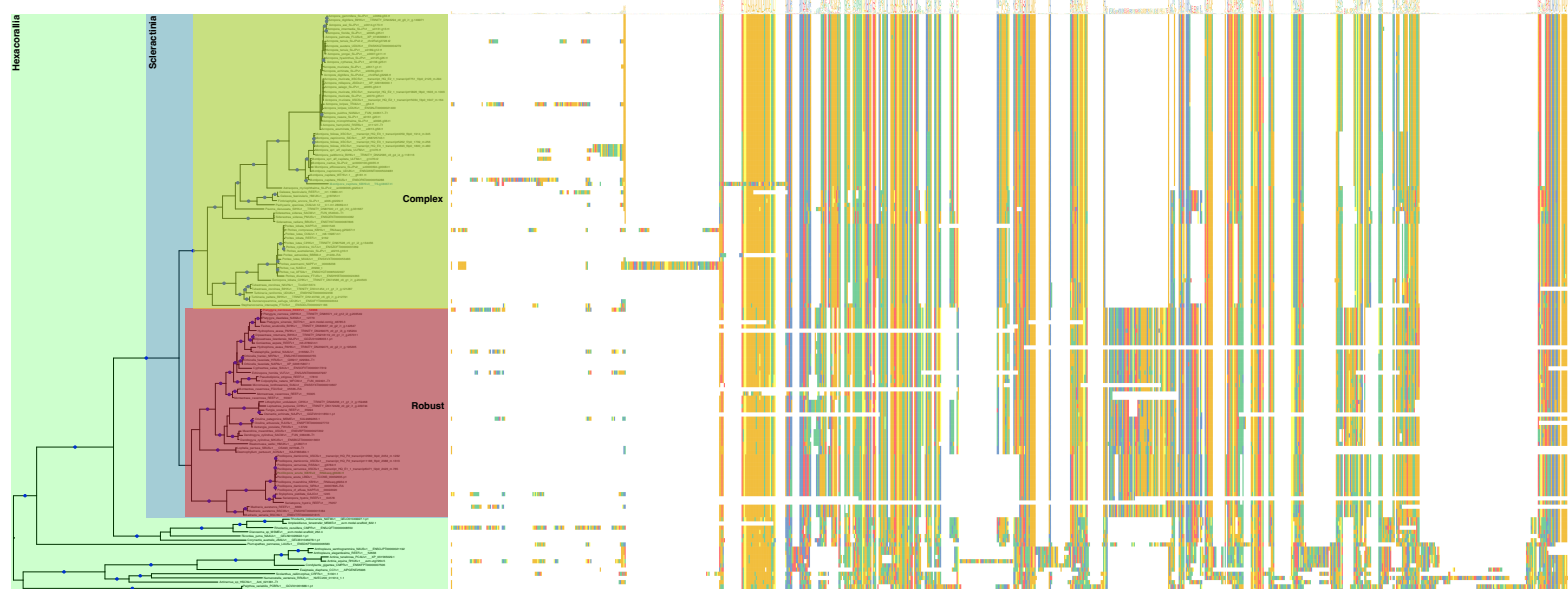**B**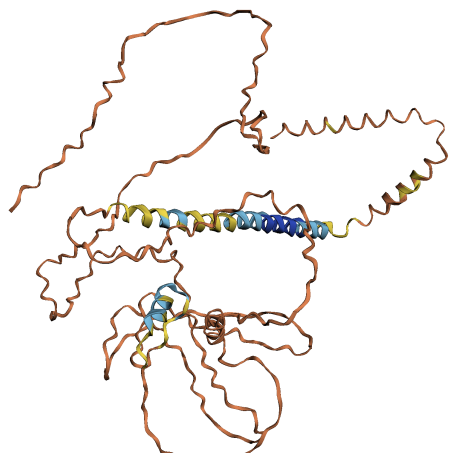**C**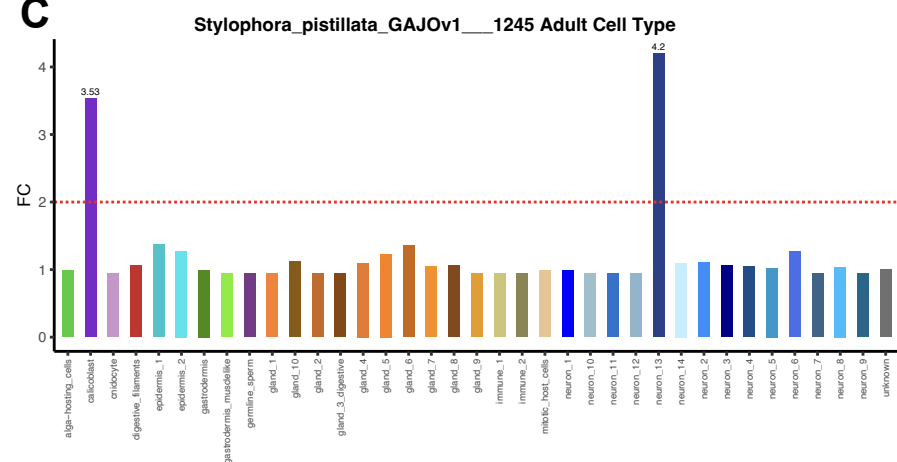**D**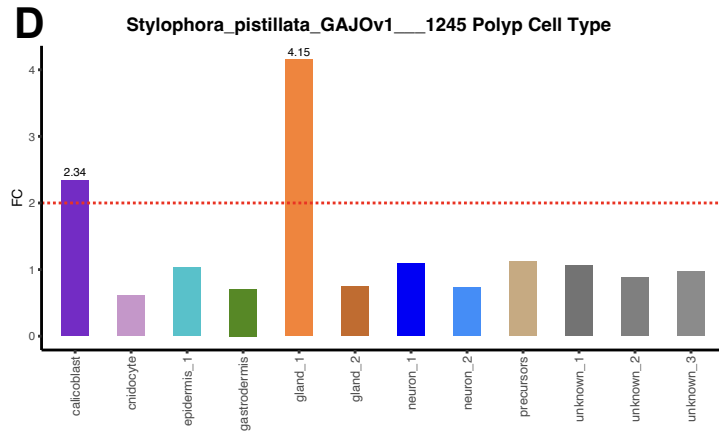**E**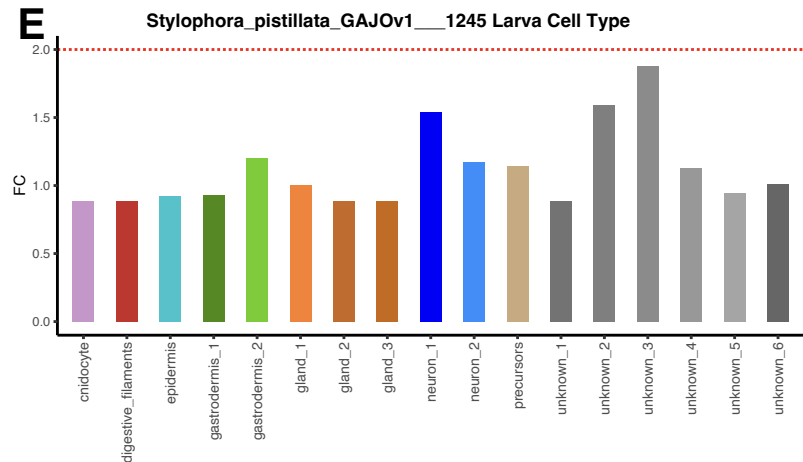**F**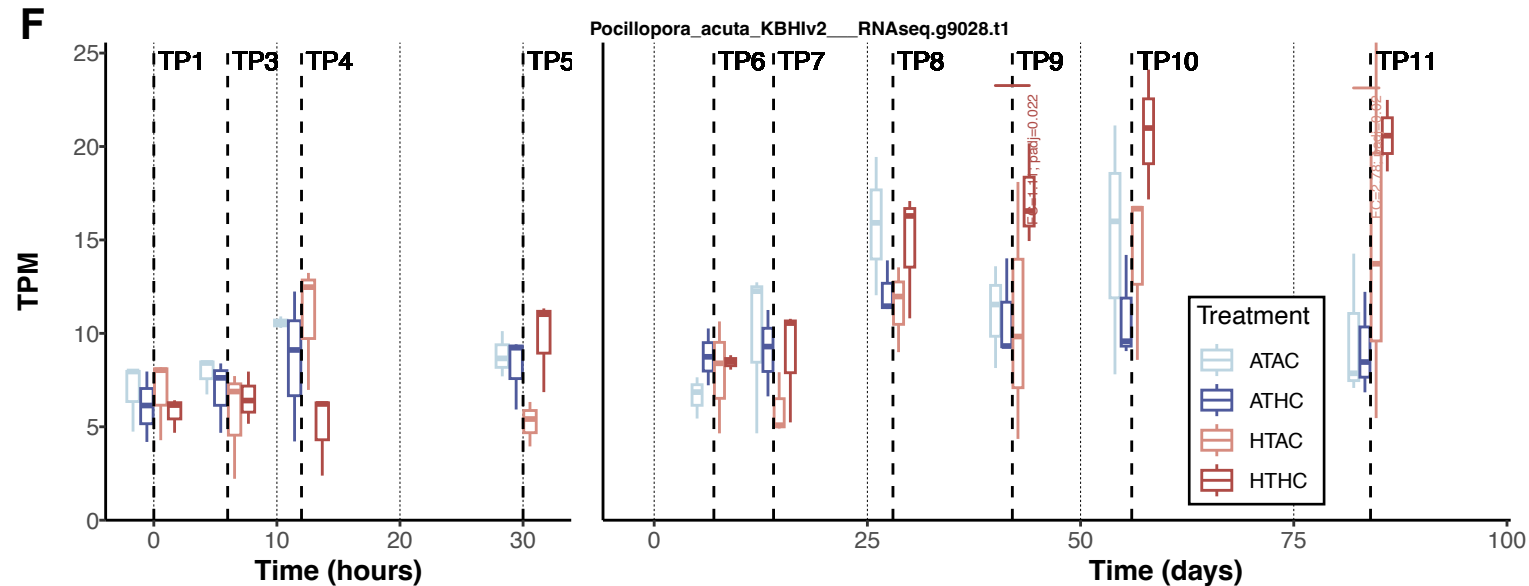

Supplement: evag072_Supplementary_Data [file evag072_supplementary_data.zip › Dataset_S3.pdf]

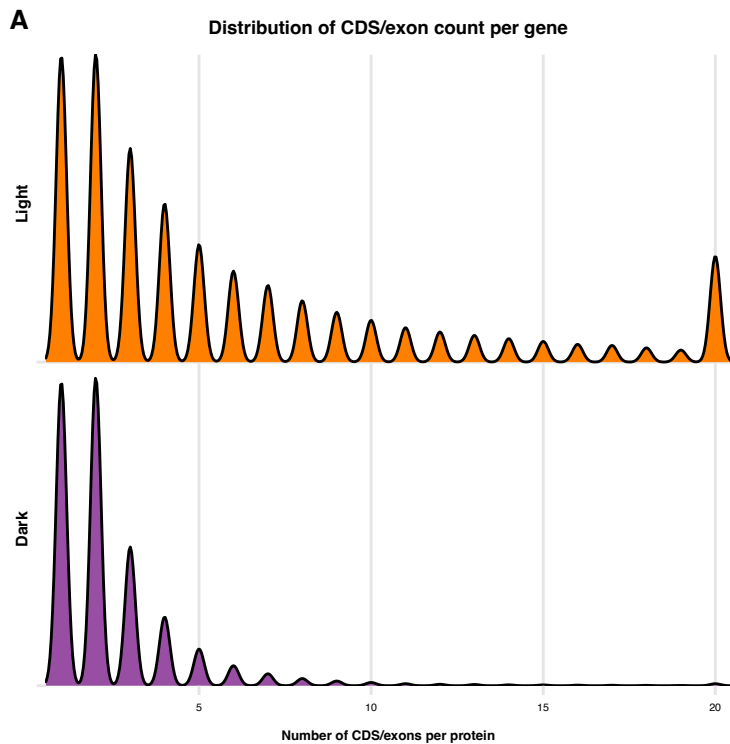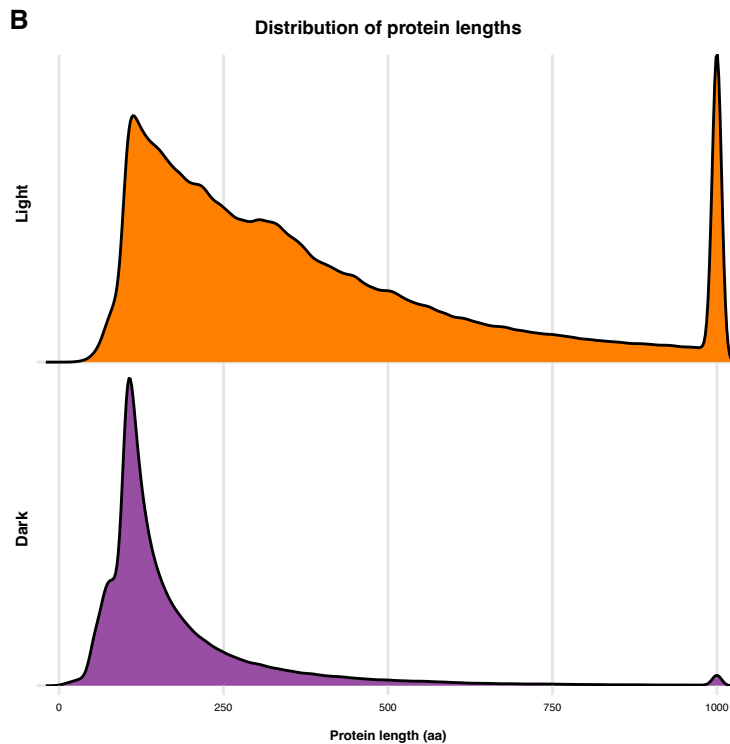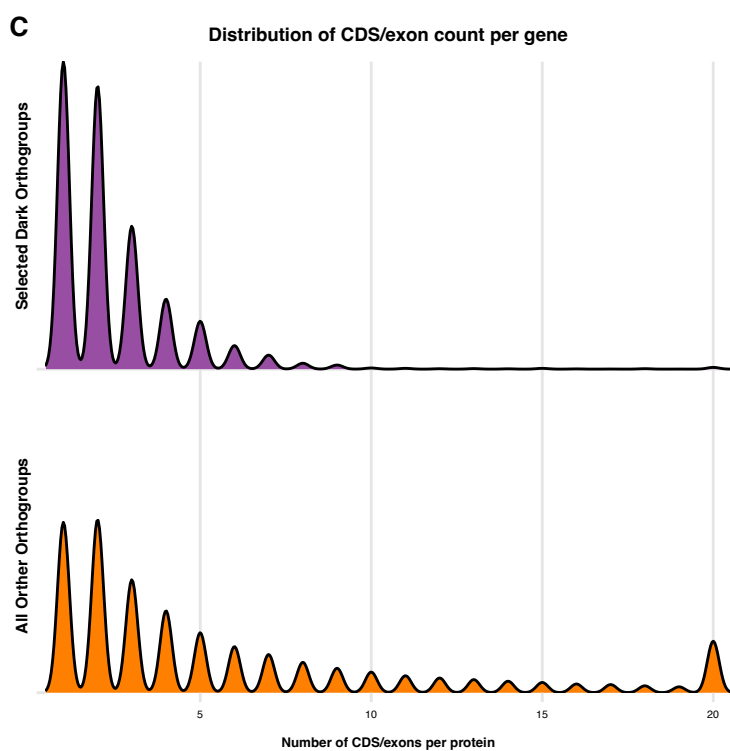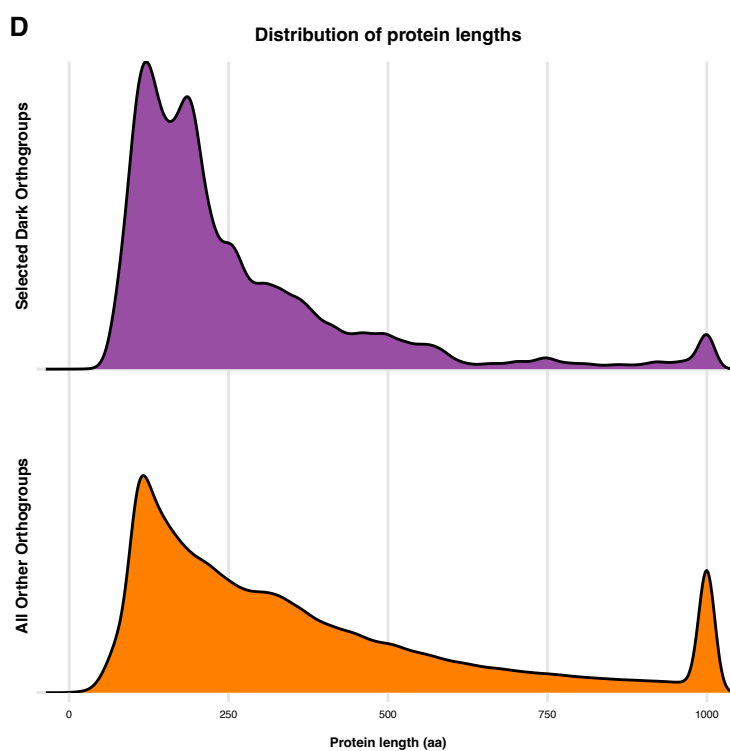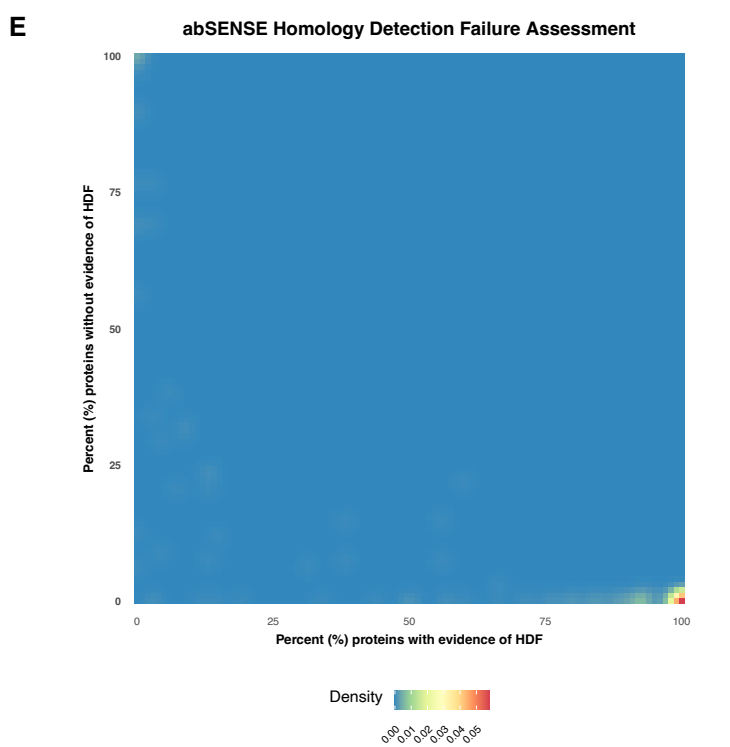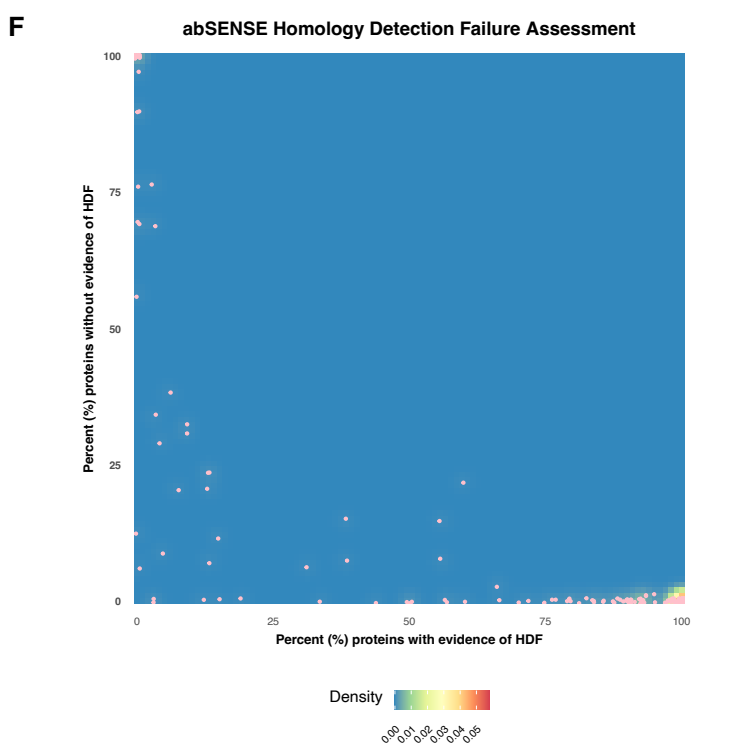



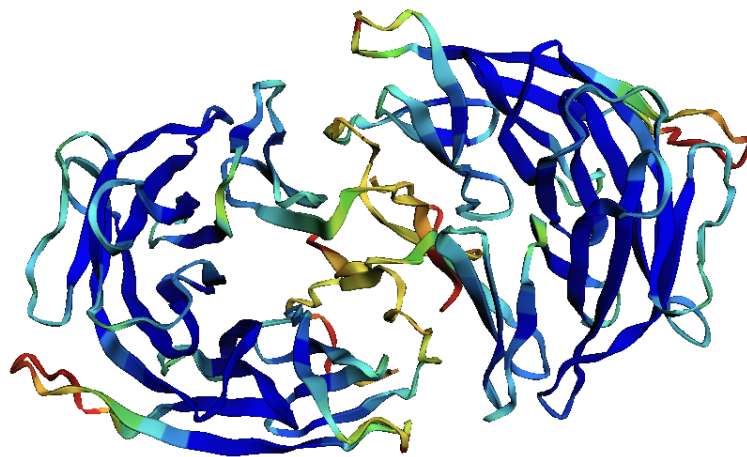

pLDDT: ■ Very low (<50) ■ Low (60) ■ OK (70) ■ Confident (80) ■ Very high (>90)

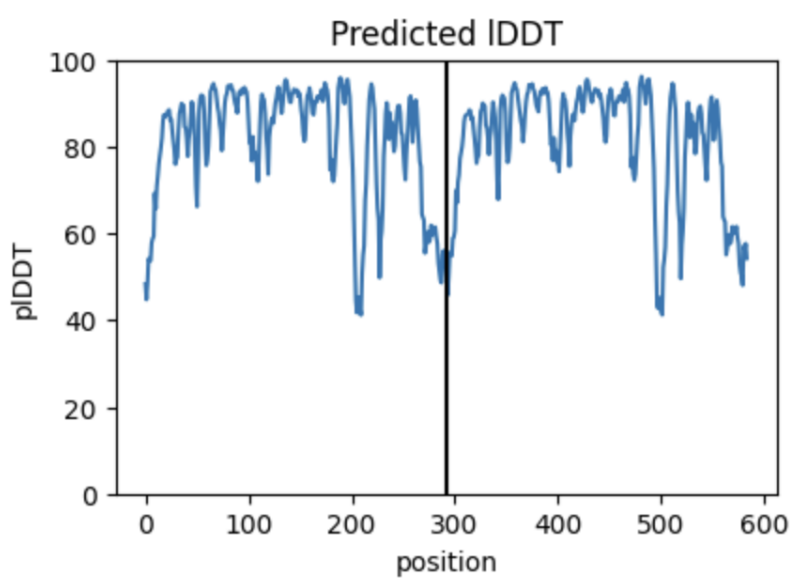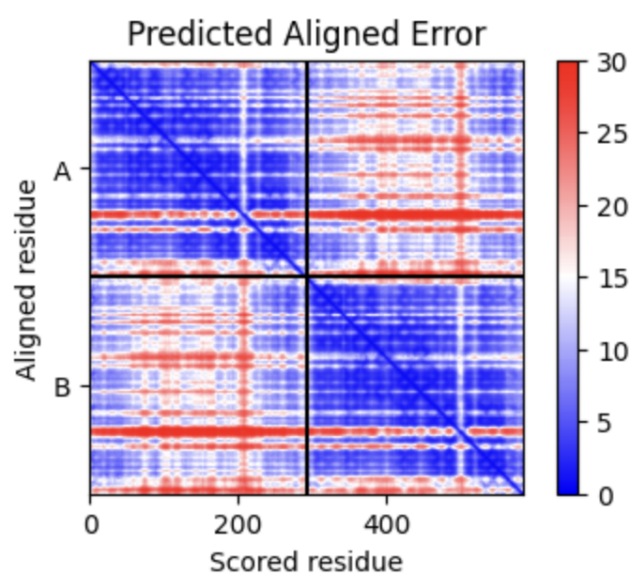

Supplement: evag072_Supplementary_Data [file evag072_supplementary_data.zip › Stephens_etal_2026_Supplemental_Figures_R3.pdf]

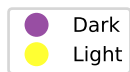

Supplement: evag072_Supplementary_Data [file evag072_supplementary_data.zip › Dataset_S4/Dataset_S4/Figure_S4A.designation.legend.pdf]

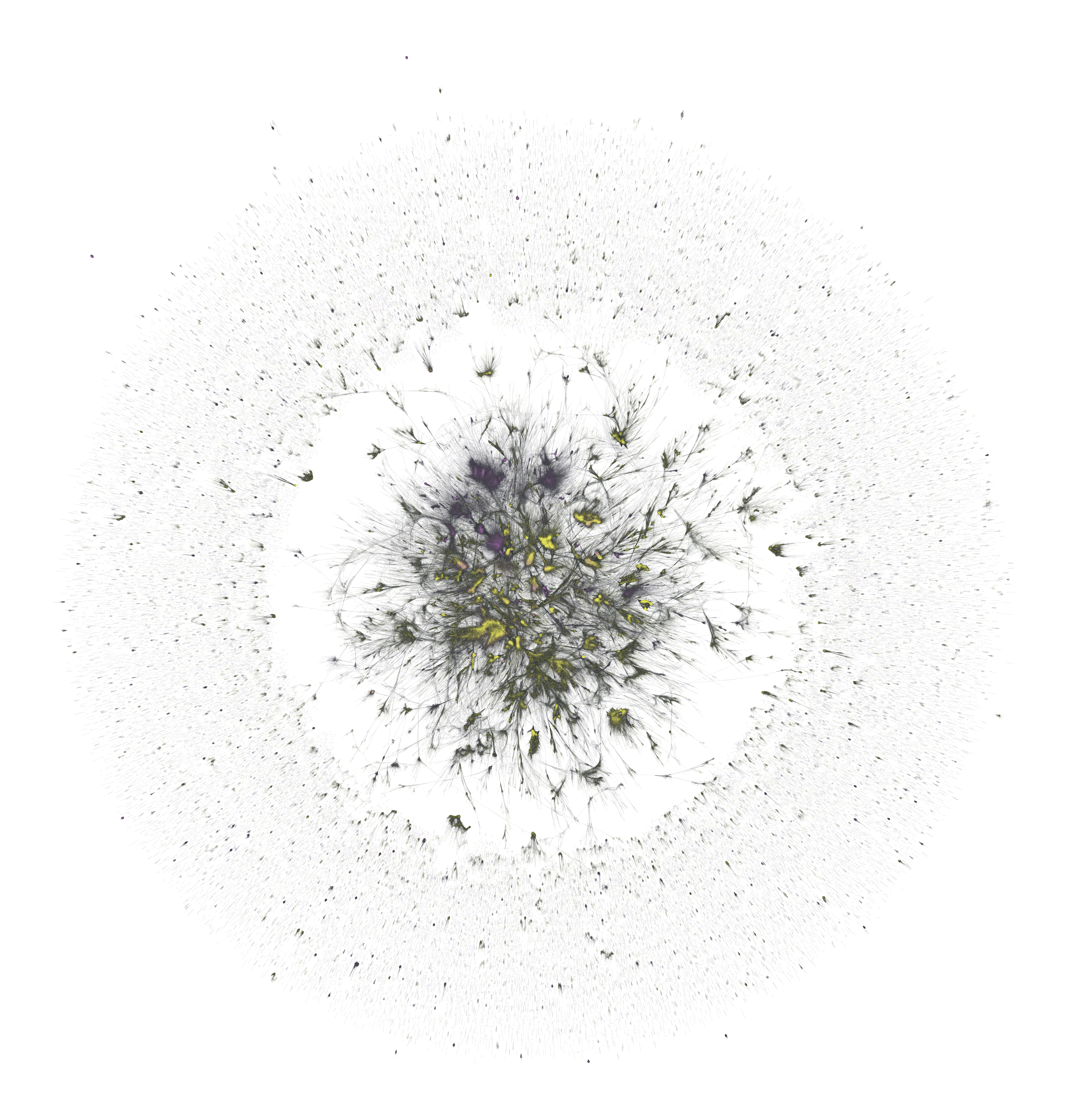

Supplement: evag072_Supplementary_Data [file evag072_supplementary_data.zip › Dataset_S4/Dataset_S4/Figure_S4A.designation.png]

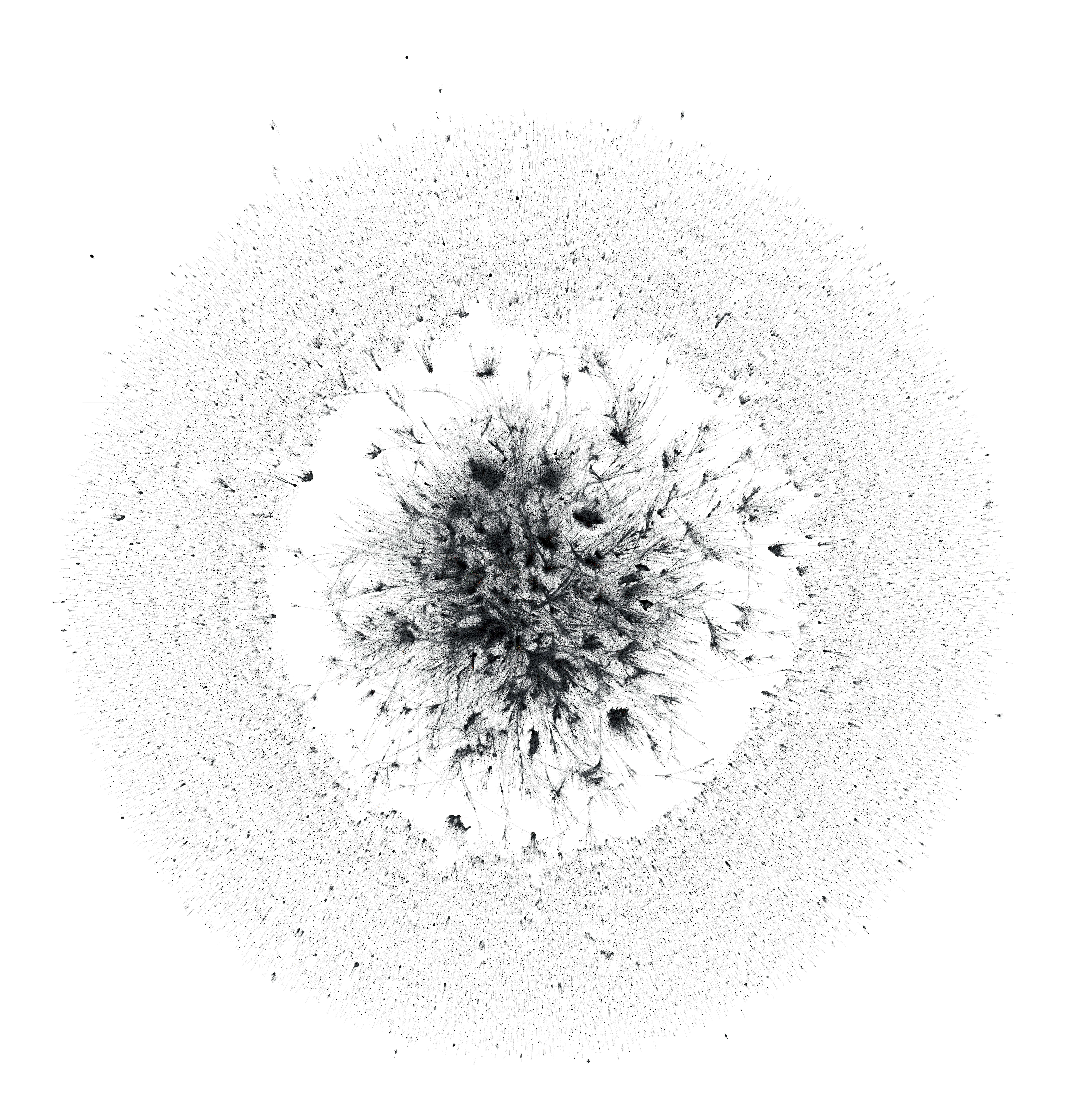

Supplement: evag072_Supplementary_Data [file evag072_supplementary_data.zip › Dataset_S4/Dataset_S4/Figure_S4B.RNA_piechart.png]

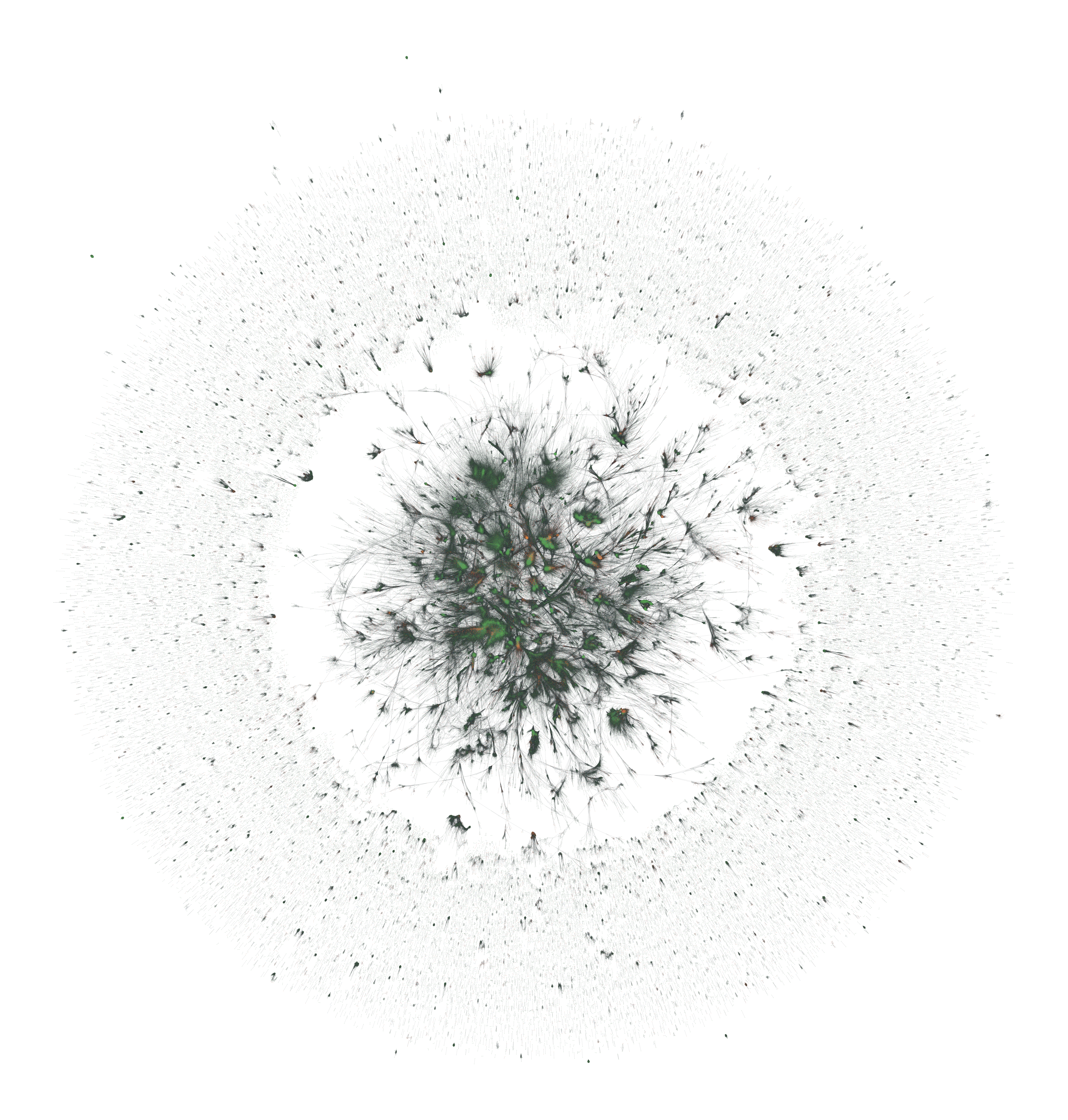

Supplement: evag072_Supplementary_Data [file evag072_supplementary_data.zip › Dataset_S4/Dataset_S4/Figure_S4C.best_strata.png]

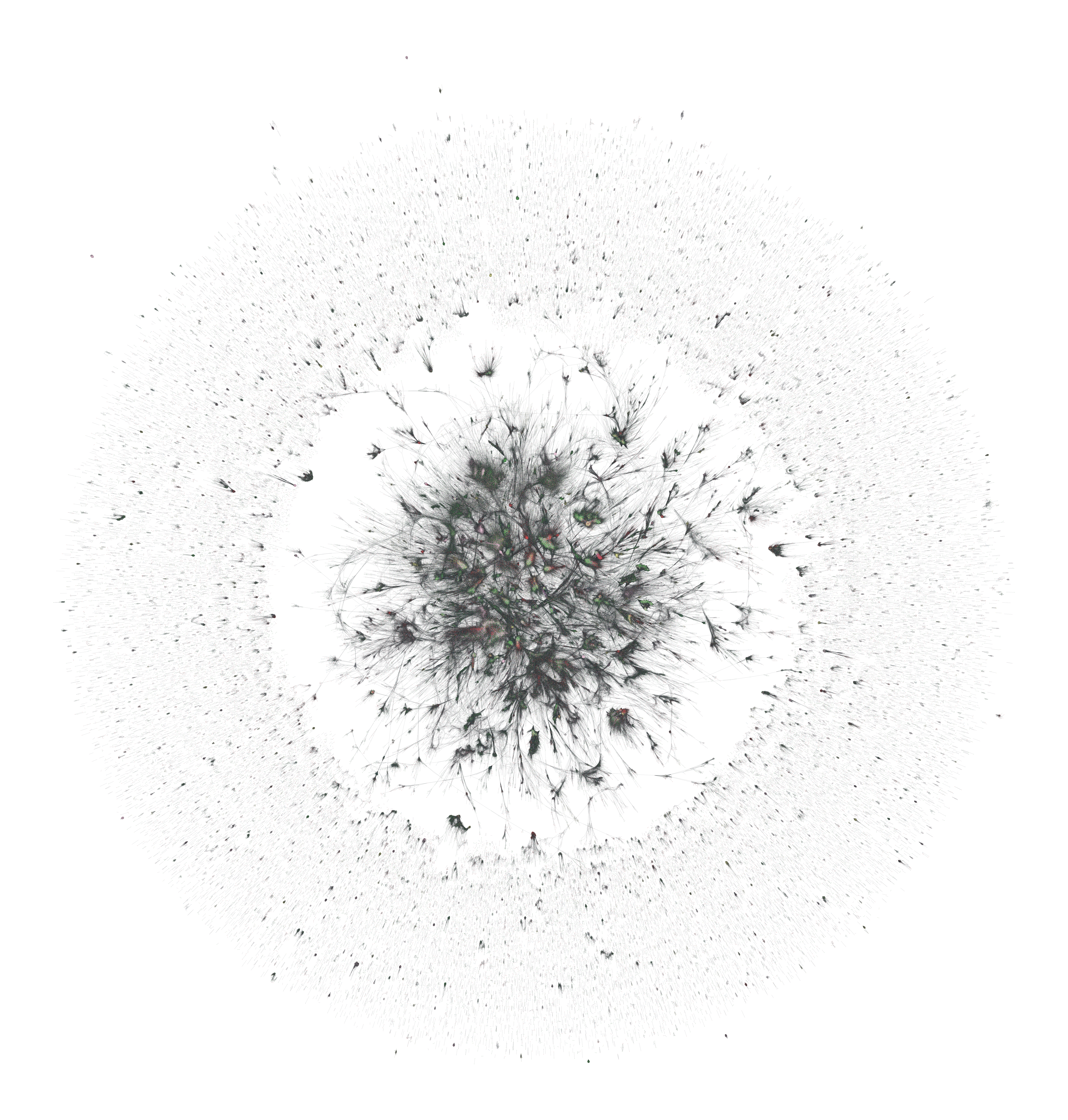

Supplement: evag072_Supplementary_Data [file evag072_supplementary_data.zip › Dataset_S4/Dataset_S4/Figure_S4D.best_strata_taxa.png]
